# Genomic analysis of the tribe Emesidini (Lepidoptera: Riodinidae)

**DOI:** 10.11646/zootaxa.4668.4.2

**Published:** 2019-09-12

**Authors:** JING ZHANG, JINHUI SHEN, QIAN CONG, NICK V. GRISHIN

**Affiliations:** 1 Departments of Biophysics and Biochemistry, University of Texas Southwestern Medical Center, 5323 Harry Hines Blvd, Dallas, TX, USA 75390-9050; 2 present address: Institute for Protein Design and Department of Biochemistry, University of Washington, 1959 NE Pacific Street, HSB J-405, Seattle, WA, USA 98195; 3 Howard Hughes Medical Institute, 5323 Harry Hines Blvd, Dallas, TX, USA 75390-9050

**Keywords:** biodiversity, genomic sequencing, museomics, phylogeny, metalmark butterflies

## Abstract

We obtained and phylogenetically analyzed whole genome shotgun sequences of nearly all species from the tribe Emesidini Seraphim, Freitas & Kaminski, 2018 (Riodinidae) and representatives from other Riodinidae tribes. We see that the recently proposed genera *Neoapodemia* Trujano, 2018 and *Plesioarida* Trujano & García, 2018 are closely allied with *Apodemia* C. & R. Felder, [1865] and are better viewed as its subgenera, **new status**. Overall, *Emesis* Fabricius, 1807 and *Apodemia* (even after inclusion of the two subgenera) are so phylogenetically close that several species have been previously swapped between these two genera. **New combinations** are: *Apodemia (Neoapodemia) zela* (Butler, 1870), *Apodemia (Neoapodemia) ares* (Edwards, 1882), and *Apodemia (Neoapodemia) arnacis* (Stichel, 1928) (not *Emesis*); and *Emesis phyciodoides* (Barnes & Benjamin, 1924) (not *Apodemia*), assigned to each genus by their monophyly in genomic trees with the type species (TS) of the genus. Surprisingly, we find that *Emesis emesia* Hewitson, 1867 is not grouped with *Emesis*, but in addition to *Apodemia* forms a third lineage of similar rank, here named *Curvie* Grishin, **gen. n.** (TS: *Symmachia emesia* Hewitson, 1867). Furthermore, we partition *Emesis* into 6 subgenera (4 new): *Emesis* (TS: *Hesperia ovidius* Fabricius, 1793, a subjective junior synonym of *Papilio cereus* Linnaeus, 1767), *Aphacitis* Hübner, [1819] (TS: *Papilio dyndima* Cramer, [1780], a subjective junior synonym of *Papilio lucinda* Cramer, [1775]), *Poeasia* Grishin, **subgen. n.** (TS: *Emesis poeas* Godman, [1901]), *Mandania* Grishin, **subgen. n.** (TS: *Papilio mandana* Cramer, [1780]), *Brimia* Grishin, **subgen. n.** (TS: *Emesis brimo* Godman & Salvin, 1889), and *Tenedia* Grishin, **subgen. n.** (TS: *Emesis tenedia* C. & R. Felder, 1861). Next, genomic comparison of primary type specimens suggests **new status** for *Emesis vimena* Schaus, 1928 as a subspecies of *Emesis brimo* Godman & Salvin, 1889, *Emesis adelpha* Le Cerf, 1958 with *E. a. vicaria* Le Cerf, 1958 are subspecies of *Emesis heteroclita* Stichel, 1929, and *Emesis tristis* Stichel, 1929 is not a synonym of *E. brimo vimena* but of *Emesis lupina* Godman & Salvin, 1886. A **new status** of a species is given to the following taxa: *Emesis furor* A. Butler & H. Druce, 1872 (not a subspecies of *E. mandana* (Cramer, 1780)), *Emesis melancholica* Stichel, 1916 (not a subspecies of *E. lupina* Godman & Salvin, 1886), *Emesis progne* (Godman, 1903) (not a subspecies of *E. brimo* Godman & Salvin, 1889), and *Emesis opaca* Stichel, 1910 (not a synonym of *E. lucinda* (Cramer, 1775)). *Emesis castigata diringeri* Gallard 2008 is a subjective junior synonym of *E. opaca*, **new status**. Finally, *Xanthosa* Grishin, **gen. n.** (TS: *Charmona xanthosa* Stichel, 1910) is proposed for a sister lineage of *Sertania* Callaghan & Kaminski, 2017 and *Befrostia* Grishin, **gen. n.** (TS: *Emesis elegia* Stichel, 1929) is proposed for a clade without apparent phylogenetic affinities that we place in Befrostiini Grishin, **trib. n**. In conclusion, genomic data reveal a number of errors in the current classification of Emesidini and allow us to confidently reclassify the tribe partitioning it in three genera: *Apodemia*, *Curvie* gen. n. and *Emesis*.

## Introduction

Metalmark butterflies (family Riodinidae) are distributed worldwide, but the majority of species are found in the Neotropics (Callaghan and Lamas 2004). The family was recognized as a group by Bates, although by a different name (Bates 1868). Stichel comprehensively revised the family, and his pioneering works formed the basis for our current knowledge (Stichel 1910–1911; 1928; 1930–1931). Harvey refined the higher classification of Riodinidae applying phylogenetic methods to morphological characters (Harvey 1987). However, metalmarks are particularly diverse in their wing shapes and patterns making them challenging to classify based on morphology. DNA-based phylogenies can be more revealing and two larger-scale studies have been instrumental for our understanding of this family (Espeland *et al*. 2015; Seraphim *et al*. 2018). These studies revealed several unsuspected evolutionary connections and suggested that a number of species have been misclassified.

Metalmarks are most species-rich in tropical regions, and only several phylogenetic lineages extend to the north. One of these clades is the tribe Emesidini Seraphim, Freitas & Kaminski, 2018. This tribe was proposed as Emesini by Stichel (1911) and in addition to *Emesis* Fabricius, 1807 and *Apodemia* C. & R. Felder, 1865, the two genera the tribe is currently composed of, included a number of others, since then transferred to other tribes. Harvey (1987) noted the similarity between *Emesis* and *Apodemia* in the position of the silk girdle in their pupae, but placed these and several other genera as ‘Emesini’ incertae sedis. Regardless, the name Emesini is a junior homonym of Emesini Amyot and Serville, 1843 (Hemiptera) and thus is invalid, so a new name Emesidini was proposed in Seraphim et al. (2018). While this tribe is still most diverse in the Neotropics, its northern offshoot *Apodemia* reaches Canada. This genus has been critically re-evaluated recently and split into 3 genera, based in part on the evidence from DNA sequences (Trujano-Ortega *et al*. 2018).

With the advent of genomic sequencing, it becomes feasible to look at the complete genotypes of organisms and learn about their evolution at the level not previously possible (Li *et al*. 2019). The genomic landscape of a phylogenetic group chosen for the study reveals many unsuspected nuances with implications for its classification. Groups thought to be monophyletic may not be such, and species dissimilar in their appearance may turn out to be close relatives. DNA-based revision also prompts us to think about consistent and more objective criteria to define taxonomic groups and their ranks (Li *et al*. 2019; Talavera *et al*. 2012). These methods seem to produce meaningful results, because genetic differentiation leads to phenotypic divergence that was used previously to outline genera by morphology. Understanding correlation between genomic differences and phenotypic divergence is an emerging field of research (Casanova *et al*. 2018; Costanzo *et al*. 2019).

In this study, we applied the methods of genomics to the tribe Emesidini. We sequenced and analyzed genomic data for nearly all species, including a number of primary type specimens, and placed it in the phylogenetic context of all Riodinidae by sequencing representatives of other tribes and subtribes. The most surprising result was the need for a new genus for *Emesis emesia* (Hewitson, 1867), which became the focus of this study. In addition, we classify *Emesis* into subgenera, find and place some species that do not belong to it in new genera and even define a new tribe. We conclude that genomic approaches bring much needed insights into evolution of Emesidini and allows to improve their classification.

## Materials and Methods

Methods used in this study are essentially identical to those described by us in previous publications (Cong *et al*. 2015; Shen *et al*. 2015; Zhang *et al*. 2019) and are particularly detailed in the SI Appendix to our recently published study (Li et al. 2019). In brief, DNA was extracted from legs of specimens, genomic libraries were constructed and sequenced for 150 bp from both ends targeting 7 Gbp of data on Illumina HiSeq x10 at GENEWIZ. The resulting reads were matched using Diamond (Buchfink *et al*. 2015) to the exons of the reference genome of *Calephelis nemesis* (Cong *et al*. 2017) we have obtained previously. Coding regions of mitochondrial genome were assembled similarly. Exons expected to be from the Z chromosome were predicted assuming similar syntenic arrangement with *Heliconius* (Heliconius Genome Consortium 2012). This assumption is reasonable due to the deep conservation of Z chromosome in Lepidoptera (Fraisse *et al*. 2017). Phylogenetic trees were generated from 3 sets of exons: whole nuclear genome, whole mitochondrial genome and Z-chromosome using RAxML-NG (Kozlov *et al*. 2018) with default parameters (-m GTRGAMMA). The data used in this project were deposited at NCBI Sequence Read Archive with accession PRJNA549759.

We sampled 52 species placed in Emesidini prior to this work, totaling 66 specimens. Most species were represented by one specimen. However, nine specimens from all parts of the range and of different ages were sequenced for *Emesis emesia* (Hewitson, 1867), because we noticed a potential taxonomic problem with this species and wanted to study it more rigorously. We did not have DNA samples for only four species: *Apodemia planeca* R. de la Maza & J. de la Maza, 2017, *Apodemia selvatica* R. de la Maza & J. de la Maza, 2017, *Emesis sinuata* Hewitson, 1877 and *Emesis toltec* Reakirt, 1866, all others were used in our analysis. In addition, 22 Riodinidae were selected as outgroups, representing all tribes and most subtribes of the family. The entire tree was rooted with *Curetis bulis* (Westwood, 1852) (Lycaenidae). Nine names where represented by their primary type specimens. Data about these specimens are summarized in the Table S1 (see supplementary file).

We identified diagnostic DNA characters in nuclear genomic sequences using our recently published procedure (see SI Appendix to Li *et al*. 2019). We found those positions in exons that were most likely to be synapomorphic for the clade being diagnosed. For a clade with several specimens sequenced, positions that are invariant in all species from this clade and have a base pair different from the (mostly invariant) base pair in all other clades were found, and those with the smallest number of species with missing data were selected. If the clade had only one or two specimen sequenced, we detected synapomorphic characters for its sister clade, taking not that base pair as the character state, and added these characters to the synapomorphic characters for the clade that leads to the common ancestor of this single specimen clade and its sister clade. The union of these characters was used to diagnose the taxon. This more sophisticated treatment increases the chances that the character found is not a random non-conserved change or a sequencing error. Moreover, number of sequence reads covering this position was accounted for in choosing the characters, and priority was given to positions with better coverage. The character states are given in diagnoses below as abbreviations. E.g., cne1547.14.1:T789C means position 789 in exon 1 of gene 14 from scaffold 1547 of *Calephelis nemesis* (cne) reference genome (Cong *et al*. 2017) is C, changed from T in the ancestor. When characters were found for the sister clade of the diagnosed taxon, the following statement was used: cne1086.2.12: G82G (not A), which means that position 82 in exon 12 of gene 2 on scaffold 1086 is occupied by the ancestral base pair G, which was changed to A in the sister clade (so it is not A in the diagnosed taxon). 145A, means position 145 is A, but the ancestral state is unclear. The sequences of exons from the reference genome with the positions used as character states highlighted in green are given in the Supplementary file. Distribution of these sequences together with this publication ensures that the numbers given in the diagnoses can be easily associated with actual sequences, which can be found in other genomic-scale datasets, or amplified with specifically developed primers. Furthermore, we provide a list of characters detected in the standard COI barcode region of 658 positions as defined previously (Ratnasingham and Hebert 2007).

## Results

**FIGURE 1.**
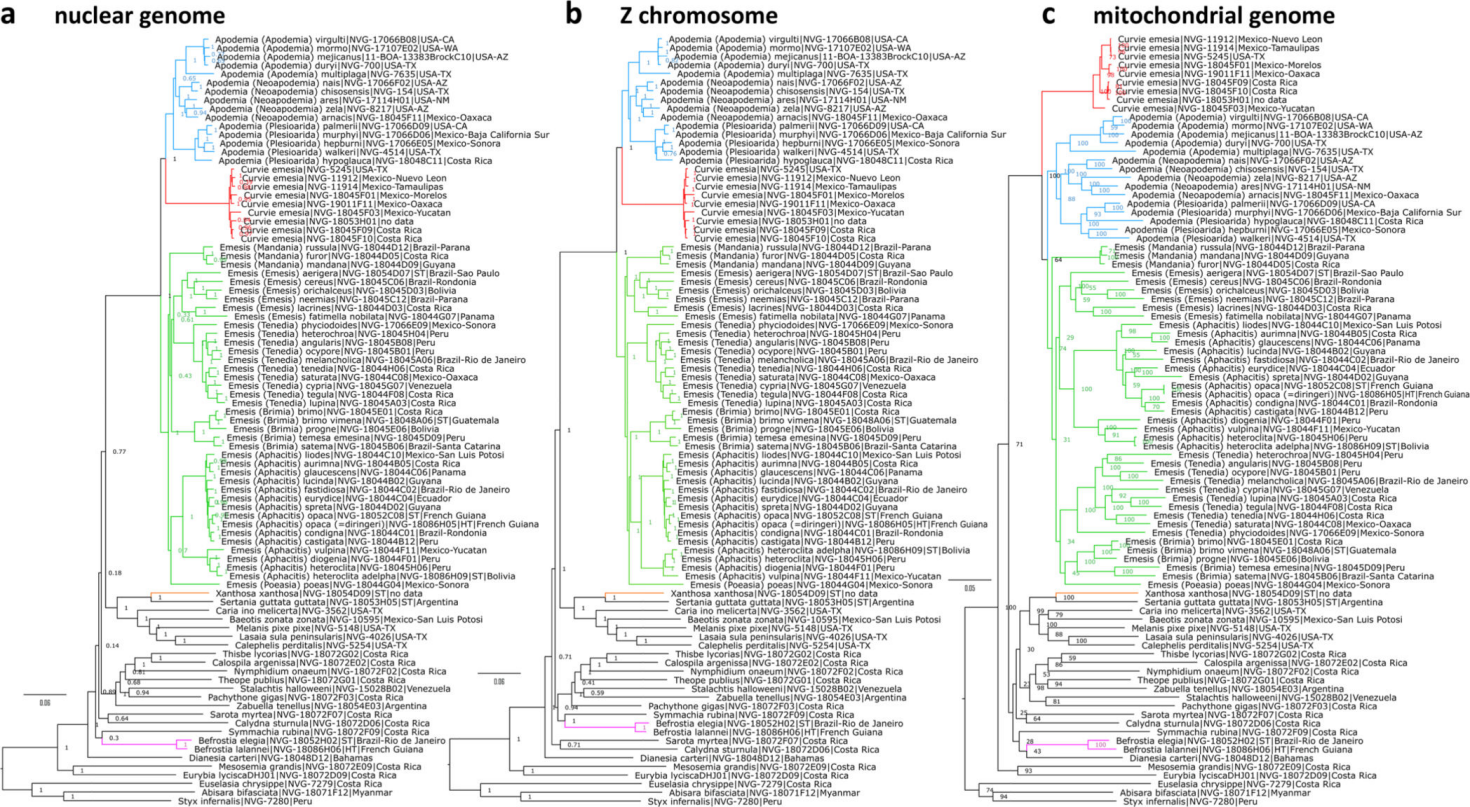
**Phylogenetic trees** constructed from protein-coding regions in **a** nuclear genomes, **b** Z-chromosome, and **c** mitochondrial genomes. The trees are rooted with *Curetis bulis* (Westwood, 1852) (NVG-7266 from Myanmar, see Table S1 for other data), not shown in the trees. Clades discussed in the text are colored.

We assembled protein-coding regions from the whole genome shotgun reads of 90 specimens (see Table S1 in the supplementary file for data). The lengths of resulting genomic regions were: nuclear total 9,958,131 +/−2,567,633; Z-chromosome 377,084 +/−111,574; mitogenomes 10,890 +/−388. Phylogenetic trees were constructed from coding regions of nuclear genome, Z chromosome and mitogenome. All the trees lead to the same major conclusions (Fig. 1). First, we confirm that the tribe Emesidini forms a confident and prominent clade distant from other Riodinidae. Second, we see that recently described genera *Neoapodemia* Trujano, 2018 and *Plesioarida* Trujano & García, 2018 group closely with *Apodemia* C. & R. Felder, [1865] (COI barcode difference about 6%−7%) and in our view are best considered as its subgenera. Third, we find that neither *Emesis* Fabricius, 1807 nor *Apodemia* in a traditional sense are monophyletic. However, their paraphyly is easily restored by (1) transferring three *Emesis* species to *Apodemia* to form new combinations: *Apodemia (Neoapodemia) zela* (Butler, 1870), *Apodemia (Neoapodemia) ares* (Edwards, 1882), and *Apodemia (Neoapodemia) arnacis* (Stichel, 1928), and (2) one *Apodemia* species to *Emesis* to become *Emesis phyciodoides* (Barnes & Benjamin, 1924), and (3) removing *E. emesia* from *Emesis* to place in a new genus named here.

### *Curvie* Grishin, gen. n.

http://zoobank.org/8C58491C-7820–43C0–8ADB-DF7FCA0E21BB

### Type species:

*Symmachia emesia* **Hewitson, 1867.**

#### Diagnosis.

**FIGURE 2.**
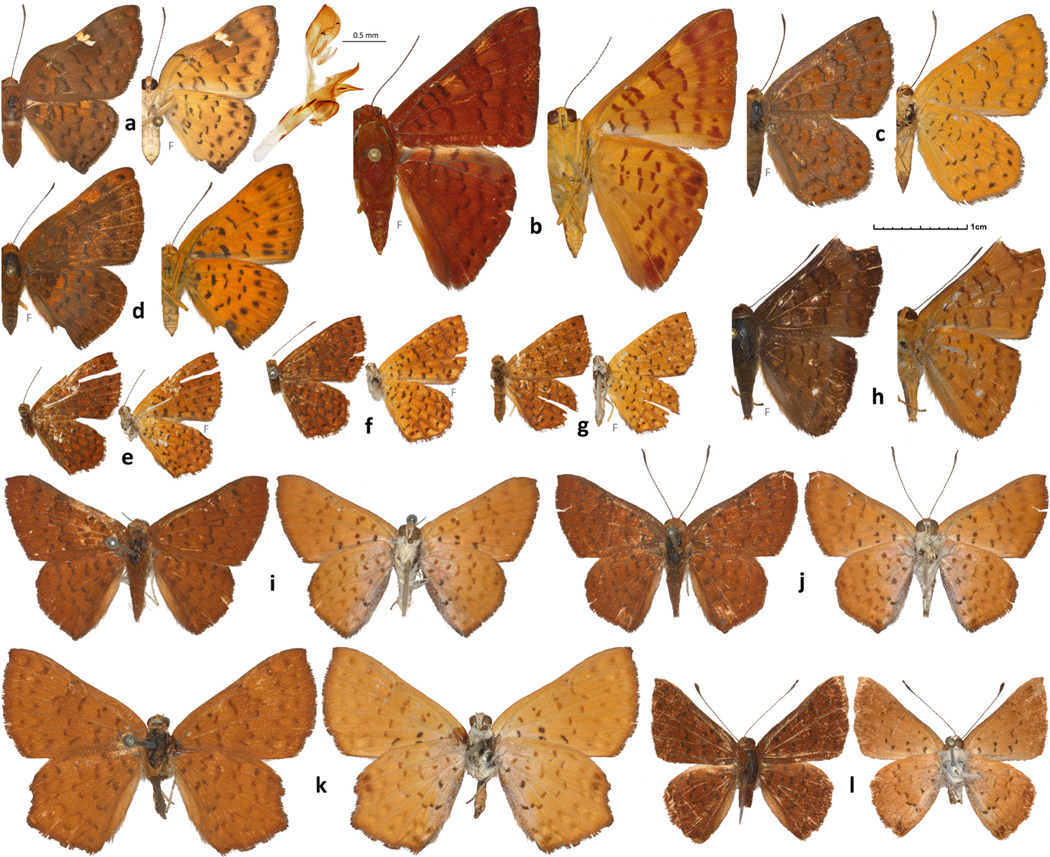
Sequenced specimens of new genera and subgenera proposed in this study. Dorsal and ventral sides are shown on the left and right, respectively, with the image letter in between. Gray letter F indicates that the mirror image (left-right inverted) is shown. DNA sample numbers are indicated for specimens, see Table S1 for other data. **a**. *Curvie emesia*, NVG-18045F09, on the right are male genitalia (with a 0.5 mm scale bar) of the specimen NVG-11913, data as for NVG-11914 (Table S1), but eclosed on 15-Feb-1974; **b**. *Emesis (Mandania) mandana*, NVG-18044D09; **c**. *Emesis (Poeasia) poeas*, NVG-18044G04; **d**. *Emesis (Brimia) brimo*, NVG-18045E01; **e**. *Xanthosa xanthosa* syntype, NVG-18054D08; **f**. *Xanthosa xanthosa* syntype, NVG18054D09; **g**. *Xanthosa xanthosa* syntype, NVG-18054D10; **h**. *Emesis (Tenedia) tenedia*, NVG-18086H06; **i**. *Befrostia elegia* syntype male, NVG-18052H02; **j**. *Befrostia elegia* syntype male, Brazil, coll. H. Stichel, #1089, NVG-18052H04; **k**. *Befrostia elegia* syntype female, no data, NVG-18052H03; **l**. *Befrostia lalannei* holotype, NVG-18086H06. All syntypes shown are from the ZMHB collection.

Distinguished from its relatives by strongly curved forewing costal margin: prominently concave near its middle, marked (distad of broken dark discal line) by a white bar from costa to vein M_2_, but convex near base and near apex, and the lack of metallic markings (Fig. 2a). Similar, but less concave, costal margin in some *Emesis*, but in these species costal white bar absent or bar yellower, from costa to M_1_ vein, closer to apex, and wings with metallic spots. Females with wings rounder and paler colored above than males. In DNA, a combination of the following base pairs is diagnostic: nuclear genome: cne1828.1.1:A1826T, cne3461.1.14:A2393G, cne1547.14.1:T789C, cne3037.1.5:T844C, cne11073.6.5:A770T; COI barcode region: A22T, T97C, A99T, A268T, A470G, T568A.

#### Derivation of the name.

The name is a feminine noun in the nominative singular given for the curved forewing costa.

#### Species included:

Only the type species *Symmachia emesia* Hewitson, 1867 with its subjective junior synonym *Symmachia yucatanensis* Godman & Salvin, [1886].

#### Parent taxon:

Tribe Emesidini Seraphim, Freitas & Kaminski, 2018.

We see that the three trees show differences in topology (Fig. 1). In particular, mitogenome protein-coding regions are not sufficient to resolve a number of clades and support values for these are below 75%. While *Curvie* is sister to *Apodemia* in both nuclear trees, it is sister to the clade formed by *Apodemia* and *Emesis* in the mitogenome tree. However, all three genera (*Apodemia*, *Curvie* and *Emesis*) are monophyletic even in the mitogenome tree indicating closeness within each genus and their prominent separation from all others.

After the monophyly of the three genera in the tribe was assured by transferring species between *Apodemia* and *Emesis* and erecting the genus *Curvie* n. gen., we studied each genus to find meaningful phylogenetic groups to be defined as subgenera. While no additional partitions to those proposed by Trujano-Ortega et al. (2018) can be found in *Apodemia*, *Emesis* splits into 6 clades. These clades are observed in all three trees (Fig. 1). Two of them have names (*Emesis* and *Aphacitis* Hübner, [1819]), and four are described here as new.

### *Poeasia* Grishin, subgen. n.

http://zoobank.org/ACDFFDCC-6C63–49D1-B21E-F1EAE3D39E62

### Type species:

*Emesis poeas* **Godman, [1901].**

#### Diagnosis.

Distinguished from its relatives by more rounded wings, especially in females, absence of metallic dashes and presence of broader, gray and somewhat silvery bands instead (Fig. 2c). In DNA, a combination of the following base pairs is diagnostic: nuclear genome: cne703.2.8:A5082G, cne628.1.1:A2634G, cne8205.5.4:A837G, cne239.4.1:T1275C, cne2803.9.1:A528C; COI barcode region: T13C, T154C, A208T, T533C, and T562C.

#### Derivation of the name.

The name is a feminine noun in the nominative singular. It is formed from the type species name.

#### Species included:

Only the type species.

#### Parent taxon:

Genus *Emesis* Fabricius, 1807.

### *Mandania* Grishin, subgen. n.

http://zoobank.org/F2C571EB-E320–41D6-A4D2–3C5F9E466E12

### Type species:

*Papilio mandana* Cramer, [1780].

#### Diagnosis.

Distinguished from other *Emesis* by thicker, more robust bodies, larger hindwings comparatively to forewings and characteristic rustic color of the wings lacking pale bands (Fig. 2b). In DNA, a combination of the following base pairs is diagnostic: nuclear genome: cne3461.1.14:T2709C, cne1411.6.4:A2699C, cne1086.2.2: A384G, cne123.6.3:T427C, cne3461.1.14:T2338C; COI barcode region: A46T, T55A, T190A, T421C, T592A, and A637T.

#### Derivation of the name.

The name is a feminine noun in the nominative singular. It is formed from the type species name.

#### Species included:

*Papilio mandana* Cramer, [1780], *Emesis furor* Butler & Druce, 1872, and *Emesis russula* Stichel, 1910.

#### Parent taxon:

Genus *Emesis* Fabricius, 1807.

### *Brimia* Grishin, subgen. n.

http://zoobank.org/B13D38D2–529A-4A5A-99D4–0F1B02C89435

### Type species:

*Emesis brimo* Godman & Salvin, 1889.

#### Diagnosis.

Distinguished by more produced forewing apex in males and consequently smaller hindwing comparatively to forewing and a unique tone of brighter orange color of some spots and bands in most species, in particular near costa, distad of its concave middle (Fig. 2d). In species lacking orange colors, the hindwing of males still disproportionally small compared to forewing. In DNA, a combination of the following base pairs is diagnostic: nuclear genome: cne6566.2.1:T111C, cne293.8.1:T183C, cne4269.3.1:C933T, cne22329.2.1:A1358T, cne37625.2.1:G450A; COI barcode region: A67T, T265A, T415A, T499A, C530T, and T532A.

#### Derivation of the name.

The name is a feminine noun in the nominative singular. It is formed from the type species name.

#### Species included:

*Emesis brimo* Godman & Salvin, 1889, *Symmachia temesa* Hewitson, 1870, and *Symmachia satema* Schaus, 1902.

#### Parent taxon:

Genus *Emesis* Fabricius, 1807.

### *Tenedia* Grishin, subgen. n.

http://zoobank.org/44F6F565-F53A-41E4–9296-BF804919F115

### Type species:

*Emesis tenedia* C. & R. Felder, 1861.

#### Diagnosis.

A large subgenus without apparent synapomorphy. Characterized by generally broader wings than other *Emesis* (Fig. 2h). It is best diagnosed by the lack of characters for other subgenera. Differs from the nominotypical subgenus by the lack of metallic spots, from *Aphacitis* by the lack of apical white spots in females, from *Poeasia* (Fig. 2c) by the lack of broad gray semi-metallic areas on wings, from *Mandania* (Fig. 2b) by less bulky body, and from *Brimia* (Fig. 2d) by relatively larger hindwings. In DNA, a combination of the following base pairs is diagnostic: nuclear genome: cne11306.1.2:T60A, cne5833.2.1:A108G, cne12476.6.1:A395G, cne18163.3.1: A186G, cne2337.5.1:A603T; COI barcode region: T281R, T484M, G506A or G38A & T283A, and A520T.

#### Derivation of the name.

The name is a feminine noun in the nominative singular. It is formed from the type species name.

#### pecies included:

*Emesis lupina melancholica* Stichel, 1916 (elevated to species level below), *Polystichtis ocypore* Geyer, 1837, *Emesis angularis* Hewitson, 1870, *Emesis sinuata* Hewitson, 1877, *Emesis heterochroa* Hopffer, 1874, *Emesis tenedia* C. & R. Felder, 1861, *Emesis saturata* Godman & Salvin, [1886], *Emesis cypria* C. & R. Felder, 1861, *Emesis tegula* Godman & Salvin, [1886], *Emesis lupina* Godman & Salvin, [1886], *Emesis toltec* Reakirt, 1866, *Apodemia phyciodoides* Barnes & Benjamin, 1924.

#### Parent taxon:

Genus *Emesis* Fabricius, 1807.

Next, our genomic analysis revealed that some species placed in *Emesis* do not belong in that genus. In agreement with morphological analysis of Hall & Harvey (2002), genomic phylogeny places *Emesis xanthosa* (Stichel, 1919) as a sister of *Sertania guttata* (Stichel, 1910), the type species of a genus described recently (Kaminski *et al*. 2017). The difference between COI barcodes of *E*. *xanthosa* and *Sertania guttata* is about 9%, similar to the difference of 8% between two genera *Lasaia* H. Bates, 1868 and *Calephelis* Grote & Robinson, 1869, but larger that the difference of 6.5% between two subgenera *Neoapodemia* Trujano, 2018 and *Plesioarida* Trujano & García, 2018. Therefore, similarly to Kaminski *et al*. (2017), we do not place *xanthosa* in *Sertania*, but a new genus is erected for it here.

### *Xanthosa* Grishin, gen. n.

http://zoobank.org/64F97B84–97DC-434D-BAD9-B4474BFDAF50

### Type species:

*Charmona xanthosa* Stichel, 1910.

#### Diagnosis.

A sister genus to *Sertania* Callaghan & Kaminski, 2017, differing from it by it by almost evenly convex outer margin of forewing, instead of shallow-W-shaped margin, concave around forewing middle in all *Sertania* species; by more uniform orange-brown wing ground color, both above and below, and similar pattern of spots between ventral hindwing and forewing, instead of 3-toned (darker orange, paler yellowish-orange and brown) wings in *Sertania* with ventral hindwing patterned differently from forewing; and by prominent dark submarginal spots on wings above and below (Fig. 2e-g). In DNA, a combination of the following base pairs is diagnostic: nuclear genome: cne658.3.1:T1835C, cne29742.2.1:T1372C, cne8287.9.1:A925T, cne4342.2.1:C323G, cne29742.2.1: C767G; COI barcode region: A46C, C83C (not T), T121C, T190A, A205C, T278C, A346C, T391A, and T595C.

#### Derivation of the name.

The name is a feminine noun in the nominative singular. Echoes the type species name.

#### Species included:

Only the type species.

#### Parent taxon:

Tribe *Sertaniini* Seraphim, Freitas, & Kaminski, 2018.

Furthermore, and to our surprise, *Emesis elegia* Stichel, 1929, for which we sequenced primary type specimens (Fig. 2i-k), was not allied to *Emesis*. Genomic phylogeny placed this species away from all other Riodininae, right at the point of rapid diversification of the subfamily and not associating it with any tribe. Although *E*. *elegia* has a general appearance of *Emesis*, it differs from it by extensive pale overscaling at the wing bases below not present in any *Emesis* species. Also, hindwing has nearly rectangular shape in females, different from *Emesis*. Instead of being related to *Emesis*, genomics revealed another surprise. Sequencing of the *Lasaia lalannei* Gallard, 2008 holotype (Fig. 2l) revealed its close similarity to *E*. *elegia*, but the lack of association with *Lasaia* H. Bates, 1868 (Fig. 1). As discussed in the original publication (Gallard 2008), genitalia of *L. lalannei* do not agree with the placement of this species in *Lasaia*. To accommodate these differences and similarities, a new genus is proposed for these species here.

### *Befrostia* Grishin, gen. n.

http://zoobank.org/685088A6-A2B4–4381-9B2E-246D2E760256

### Type species:

*Emesis elegia* Stichel, 1929.

#### Diagnosis.

A genus without apparent relatives (Fig. 2i-l). Characterized by body white below and bases of wings overscaled with white, hindwing in particular, also along anal margin. Wings above uniformly orange-brown with black dots and dashes linked into broken lines. Hindwing of females almost square, with small lobule at vein M_3_. Antennae disproportionally long, longer than 3/4 of forewing costal margin (Fig. 2l). Genitalic characters as those given by Gallard (2008: 442 and illustrated on p. 448 as Fig. 1) for *Lasaia lalannei*: aedeagus disproportionally large, with one long cornutus; uncus broad, pointed, not concave in ventral view; valva short and rounded, no transtilla, saccus not shorter than valva. In DNA, a combination of the following base pairs is diagnostic: nuclear genome: cne2576.1.37:A2012G, cne792.13.4:A310G, cne4106.6.1:G277C, cne3195.1.6:T355A, cne2174.2.11:A93G (these 5 characters are for the clade of the two species in this genus), cne306.12.2:G89T, cne3772.18.1:C271A, cne26635.1.11:A509C (these 3 characters are for the clade leading to the ancestor of this genus plus its sister clade in Fig. 1a), cne1086.2.12:G82G (not A), cne23980.1.3:G59G (not A), cne4786.7.1:C158C (not A) (the “not” base pair in these 3 characters is for the sister clade of this genus in Fig. 1a); COI barcode region: G38A, A44T, C83C (not T), T169A, A274T, A322T, C340T, A379T, T400A, T412A, T547A, and A586T.

#### Derivation of the name.

The name is a feminine noun in the nominative singular. For the frosted overscaling below on body and bases of wings: *Be*[low]-*frost*[ed]-*ia*.

#### Species included:

*Emesis elegia* Stichel, 1929 and *Lasaia lalannei* Gallard, 2008.

#### Parent taxon:

The new tribe described right below.

Our phylogenetic analysis (Fig. 1) included representatives of all Riodinidae tribes as they were delineated by Seraphim et al. (2018). Unexpectedly, *Befrostia* did not fall into any of these tribes, but formed a deep phylogenetic lineage of a tribal rank, which is named here.

### *Befrostiini* Grishin, trib. n.

http://zoobank.org/CF47B25D-F1FA-4166-BF72–88E6EE29576F

### **Type genus:** *Befrostia* Grishin.

#### Diagnosis.

A tribe without obvious phylogenetic affinities within subfamily Riodininae. Distinguished from other Riodinidae by body white below and bases of wings overscaled with white, hindwing in particular, also along anal margin. Hindwing of females almost square, with small lobule at vein M_3_. Male genitalia characterized by disproportionally large aedeagus with one long cornutus, uncus broad, pointed, not concave in ventral view, valva short and rounded, no transtilla, saccus not shorter than valva. In DNA, a combination of the following base pairs is diagnostic: nuclear genome: cne2576.1.37:A2012G, cne792.13.4:A310G, cne4106.6.1:G277C, cne3195.1.6:T355A, cne2174.2.11:A93G, cne306.12.2:G89T, cne3772.18.1:C271A, cne26635.1.11:A509C, cne1086.2.12:G82G (not A), cne23980.1.3:G59G (not A), cne4786.7.1:C158C (not A); COI barcode region: G38A, A44T, C83C (not T), T169A, A274T, A322T, C340T, A379T, T400A, T412A, T547A, and A586T.

#### Genera included:

Only the type genus.

#### Parent taxon:

Subfamily Riodininae Grote, 1895.

Selecting specimen for the genomic analysis, we attempted to sequence as many *Emesis* species as we could find. More, some of these were represented by their primary type specimens. Analysis of primary types enables us to put our taxonomic analysis on solid footing. We find that the syntype of *Emesis vimena* Schaus, 1928 from Guatemala is tightly grouped with *Emesis brimo* Godman & Salvin, 1889 (e.g. only 0.6% difference in COI barcodes) and is better viewed as a more northern subspecies of this species. *Emesis tristis* Stichel, 1929 considered a synonym of *E. vimena*, should instead be a synonym of *Emesis lupina* Godman & Salvin, [1886]. Sequencing of primary type specimens of *Emesis adelpha* Le Cerf, 1958 and *Emesis heteroclita* Stichel, 1929 suggests their conspecificity. However, due to wing pattern differences and differences in their distributions, we view *E. adelpha* and its subspecies *E. a. vicaria* Le Cerf, 1958 as subspecies of *E. heteroclita*, rather than its synonyms.

Conversely, we find that some taxa placed as subspecies differ markedly from their nominal subspecies and should be considered distinct as the species level (new status): *Emesis furor* A. Butler & H. Druce, 1872 (not a subspecies of *E. mandana* (Cramer, 1780): not sister taxa, COI barcodes difference of about 2%), *Emesis melancholica* Stichel, 1916 (not a subspecies of *E. lupina* Godman & Salvin, 1886: not in the same clade, COI barcodes 9% different), and *Emesis progne* (Godman, 1903) (not a subspecies of *E. brimo* Godman & Salvin, 1889: COI barcodes 3.8% different). Furthermore, *Emesis opaca* Stichel, 1910 is not a synonym of *E. lucinda* (Cramer, 1775) (COI barcodes difference nearly 5%), but a valid species, new status. This change further reveals that *Emesis castigata diringeri* Gallard 2008 is not a subspecies of *E. castigata* Stichel, 1910 (COI barcodes difference about 3%), and due to genomic (Fig. 2, COI barcodes are 100% identical) and morphological similarities we suggest it to be a subjective junior synonym of *E. opaca*, new status. Both taxa are from French Guiana. We summarize our results as the following taxonomic list.

#### Taxonomic arrangement of the tribe Emesidini.

The list of species arranged into genera and subgenera resulting from our genomic analysis augmented with morphological considerations is given below. Synonymic names are included for genera and subgenera. Names treated as synonyms (genera and names of type species that are considered to be synonyms) are preceded by “=”: not followed by daggers are subjective junior synonyms; † objective junior synonyms; ‡ unavailable names (such as homonyms and nomina nuda); “preocc.” indicates preoccupied, the taxonomic order (insects) of the senior name is shown in brackets. Synonyms are attributed to subgenera. Type species (TS) for genera and subgenera are listed. For type species that are considered to be synonyms, valid names are shown in parenthesis. For valid genera and subgenera (not their synonyms), names of the type species or names which type species are considered to be synonyms of, are underlined in the list. The type of change is explained after the name (new status, new combination, new placement), and the former status or the genus of former placement is listed. Subspecies names are not listed (except those resulting from the status change in this work) pending further studies.

### Tribe Emesidini Seraphim, Freitas & Kaminski, 2018

***Apodemia*** C. & R. Felder, 1865; TS: *mormo* C. & R. Felder

Subgenus *Apodemia* C. & R. Felder, 1865; TS: *mormo* C. & R. Felder

=*Chrysobia* Boisduval, 1869; TS: =*mormonia* Boisduval, 1869 (*mormo* C. & R. Felder)

***Apodemia*** ***mormo*** (C. & R. Felder, 1859)

***Apodemia virgulti*** (Behr, 1865)

***Apodemia mejicanus*** (Behr, 1865)

***Apodemia duryi*** (W. H. Edwards, 1882)

***Apodemia multiplaga*** Schaus, 1902

Subgenus *Plesioarida* Trujano-Ortega & García-Vázquez, 2018; **new status**; TS: *walkeri* Godman & Salvin

***Apodemia palmerii*** (W. H. Edwards, 1870)

***Apodemia murphyi*** Austin, [1989]

***Apodemia hepburni*** Godman & Salvin, 1886

***Apodemia planeca*** R. de la Maza & J. de la Maza, 2017

***Apodemia*** ***walkeri*** Godman & Salvin, 1886

***Apodemia selvatica*** R. de la Maza & J. de la Maza, 2017

***Apodemia hypoglauca*** (Godman & Salvin, 1878)

Subgenus *Neoapodemia* Trujano-Ortega,2018; **new status**, was a genus; TS: *nais* W. H. Edwards

=‡*Polystigma* Godman & Salvin, 1886 (preocc. *Polystigma* Kraatz, 1880 [Coleoptera]); TS: *nais* W. H. Edwards

***Apodemia* *nais*** (W. H. Edwards, 1877)

***Apodemia chisosensis*** H. Freeman, 1964

***Apodemia ares*** (W. H. Edwards, 1882); **new combination**, was in *Emesis*

***Apodemia zela*** (A. Butler, 1870); **new combination**, was in *Emesis*

***Apodemia arnacis*** (Stichel, 1928); **new combination**, was in *Emesis*

***Curvie*** Grishin, **new genus**; TS: *emesia* Hewitson

***Curvie* *emesia*** (Hewitson, 1867); **new combination**, was in *Emesis*

***Emesis*** Fabricius, 1807; TS: =*ovidius* Fabricius, 1793 (*cereus* Linnaeus)

Subgenus *Emesis* Fabricius, 1807; TS: =*ovidius* Fabricius, 1793 (*cereus* Linnaeus)

=*Polystichtis* Hübner, [1819]; TS: *cereus* Linnaeus, 1767

=†*Tapina* Billberg, 1820; TS: =*ovidius* Fabricius, 1793 (*cereus* Linnaeus)

=‡*Polystichthis* Agassiz, 1847; TS: *cereus* Linnaeus

=*Nelone* Boisduval, 1870; TS: =*fatima* Cramer, 1780 (*cereus* Linnaeus)

***Emesis* *cereus*** (Linnaeus, 1767)

***Emesis neemias*** Hewitson, 1872

***Emesis orichalceus*** Stichel, 1916

***Emesis aerigera*** (Stichel, 1910)

***Emesis lacrines*** Hewitson, 1870

***Emesis fatimella*** Westwood, 1851

Subgenus *Mandania* Grishin, **new subgenus**; TS: *mandana* Cramer

***Emesis furor*** A. Butler & H. Druce, 1872 **new status**, was a subspecies of *E. mandana*

***Emesis* *mandana*** (Cramer, 1780)

***Emesis russula*** Stichel, 1910

Subgenus *Tenedia* Grishin, **new subgenus**; TS: *tenedia* C. & R. Felder

***Emesis melancholica*** Stichel, 1916; **new status**, was a subspecies of *E. lupina*

***Emesis ocypore*** (Geyer, 1837)

***Emesis angularis*** Hewitson, 1870

***Emesis sinuata*** Hewitson, 1877

***Emesis heterochroa*** Hopffer, 1874

***Emesis* *tenedia*** C. & R. Felder, 1861

***Emesis toltec*** Reakirt, 1866

***Emesis saturata*** Godman & Salvin, 1886

***Emesis cypria*** C. Felder & R. Felder, 1861

***Emesis tegula*** Godman & Salvin, 1886

***Emesis lupina*** Godman & Salvin, 1886

=*tristis* Stichel, 1929; new placement, was a synonym of *E. vimena*

***Emesis phyciodoides*** (W. Barnes & Benjamin, 1924); **new combination**, was in *Apodemia*

Subgenus *Poeasia* Grishin, **new subgenus**; TS: *poeas* Godman

***Emesis* *poeas*** Godman, 1901

Subgenus *Brimia* Grishin, **new subgenus**; TS: *brimo* Godman & Salvin

***Emesis* *brimo*** Godman & Salvin, 1889

*Emesis brimo vimena* Schaus, 1928; **new status**, was a species

*Emesis brimo brimo* Godman & Salvin, 1889

***Emesis progne*** (Godman, 1903); **new status**, was a subspecies of *E. brimo*

***Emesis temesa*** (Hewitson, 1870)

***Emesis satema*** (Schaus, 1902)

Subgenus *Aphacitis* Hübner, [1819]; **new status**; TS: =‡*dyndima* Cramer, 1780 (*lucinda* Cramer)

=*Nimula* Blanchard, 1840; TS: *lucinda* Cramer

***Emesis liodes*** Godman & Salvin, 1886

***Emesis aurimna*** (Boisduval, 1870)

***Emesis glaucescens*** Talbot, 1929

***Emesis* *lucinda*** (Cramer, 1775)

***Emesis fastidiosa*** Ménétriés, 1855

***Emesis condigna*** Stichel, 1925

***Emesis castigata*** Stichel, 1910

***Emesis opaca*** Stichel, 1910; **new status**, was a synonym of *E. lucinda*

=*diringeri* Gallard 2008; new placement, was a subspecies of *E. castigata*

***Emesis eurydice*** Godman, 1903

***Emesis spreta*** H. Bates, 1868

***Emesis vulpina*** Godman & Salvin, 1886

***Emesis diogenia*** Prittwitz, 1865

***Emesis heteroclita*** Stichel, 1929

*Emesis heteroclita heteroclita* Stichel, 1929

*Emesis heteroclita adelpha* Le Cerf, 1958; **new status**, was a species

*Emesis heteroclita vicaria* Le Cerf, 1958; **new combination**, was a subspecies of *E. adelpha*

## Discussion

In the absence of DNA sequences, it is not readily apparent that *Emesis emesia* is not monophyletic with *Emesis*. In particular, the similarity in wing shapes apparent between *E. emesia* and the type species of *Emesis*, *E. cereus*, and reinforced by similar wing patterns, does not raise any suspicions. Here, genomic analysis is critical. In the absence of vast DNA sequence information, grouping of *E. emesia* with *Apodemia* rather than with *Emesis* would seem spurious. Even with our large datasets we were cautious to accept the paraphyly of *Emesis*. To avoid the effects of poor sample quality, we sequenced 9 *E. emesia* specimens from its entire range from southern US to Costa Rica. To avoid negative effects possible due to poor taxon sampling, we sequenced nearly all species from the tribe Emesidini, including type species of all available genus-group names. Confirming our preliminary analyses on smaller datasets, *E. emesia* was not in the same clade with *Emesis* with very high statistical support. Even if this phylogeny is incorrect, *E. emesia* is at a larger phylogenetic distance from *Emesis* species, than they are from each other suggesting that it does not belong to *Emesis*. Therefore, proposing a new genus-group name is justified by our analysis.

A contentious issue is a rank assigned to a genus-group name. Currently, there are no objective criteria to select a clade that is a genus or a clade that is a subgenus. Several reasonable considerations include the age of the clade, its prominence (relative branch length of that clade compared to others should be larger) and agreement with the currently used classification. Intuitively, genus should correspond to major groupings above species but below tribe. For Emesidini, the first split of the nuclear trees separates well-known genera *Apodemia* and *Emesis*. So, in principle, we can divide the tribe into 2 genera. However, inclusion of *E. emesis* into *Apodemia* makes it a less prominent genus, because the branch of this clade is short. More, *E. emesia* is not phenotypically similar to *Apodemia* because it was placed in *Emesis* before. Considering its large evolutionary distance from *Apodemia*, and non-monophyly with *Emesis*, we took the next major level (3 groups) to be a genus.

It is possible to move the genus boundary closer to the leaves of the tree. Indeed, recently *Apodemia* was split into 3 genera, two of which were proposed as new: *Neoapodemia* Trujano, 2018 and *Plesioarida* Trujano & García, 2018. If these are treated as genera, to be consistent with their age, *Emesis* would need to be split into several genera as well. Would such action be desirable? The resulting small genera will contain species that are very close to each other and these units do not have clear further partitions into smaller groups. However, there are two genus-group ranks: genus and subgenus. Thus, if the genus rank is assigned to a group that cannot be meaningfully split any further, the rank of subgenus cannot be used. Number of meaningful levels in a phylogenetic tree exceeds the number of ranks in classification. Thus, it seems undesirable to further reduce the number of ranks by making subgenus level impossible due to genus level clades placed too close to the leaves. From historical perspective, it is equally undesirable to introduce many additional names within clearly defined and prominent monophyletic groups that already have the names (*Apodemia* and *Emesis*) widely in use for a century. For these reasons, we treat most prominent clades at the level below *Emesis* and *Apodemia* (and thus *Curvie*) as subgenera. We confirm recently published results (Trujano-Ortega *et al*. 2018) that the three groupings within *Apodemia* are phylogenetically meaningful, but use them at the subgenus rank. Furthermore, we propose similar level subgroups within *Emesis* that we also assign the subgenus rank.

As discussed recently (Grishin 2019), if family- or genus-group taxa are discovered using phylogenetic trees constructed from DNA sequences and their monophyly is ensured with these trees, the most direct diagnoses of these taxa would include DNA characters. The diagnosis cannot simply refer to the phylogenetic tree, because statements like “diagnosed based on DNA similarities and position in the phylogenetic tree” are not sufficient according to the ICZN Code (ICZN 1999). Article 13.1.1. of the Code requires that the diagnostic characters are explicitly stated in words. Positions in DNA sequences can be used as characters, and base pairs in these positions would be character states. An ideal DNA character would be an invariant and unique synapomorphy, i.e., a base pair that is invariantly the same in all individuals of the diagnosed taxon, and is different from all individuals in all other taxa. Such characters are challenging to find, especially when only a small number of individuals are sequenced. To maximize the probability that the characters are indeed meaningful, we developed a sophisticated method that finds several highly conserved positions in genomic regions that are most accurately sequenced (see Materials and Methods). High conservation of the position outside the clade being diagnosed increases the probability that the base pair change occurred in the last common ancestor of the diagnosed clade and thus indeed is a likely synapomorphy. Several of such characters are found for each taxon and are listed in the diagnosis section in addition to morphological characters. We believe that DNA-based diagnoses may be more meaningful and may be more robust to extrapolation than morphological characters, when additional (yet unknown or not included in the analysis) species that belong to the taxon and discovered and/or sequenced. At the very least, they complement morphological diagnoses with orthogonal evidence.

## Supplementary Material

supplemental materials
