# Supplementary material for: Genomic analysis of the tribe Emesidini (Lepidoptera: Riodinidae)
Source: Zootaxa. Author manuscript; Available in PMC 2022 Mar 28. (PMC8958898; doi:10.11646/zootaxa.4668.4.2)
Supplement: supplemental materials [file NIHMS1766733-supplement-supplemental_materials.pdf]

Table S1. Data for 89 sequenced Riodinidae specimens and an outgroup

#	DNA Voucher	Taxon name	Type	Sex	Locality, date, collectors	Collection	Collection No.
1	NVG-17107E02	*Apodemia (Apodemia) mormo*		M	USA: WA, Kittitas Co., 25-Aug-1990	BMUW	
2	NVG-17066B08	*Apodemia (Apodemia) virgulti*		M	USA: CA, Los Angeles Co., Lake Hughes Rd., E Lake Castak, 7-Oct-2007, Ken Davenport	CSUC	CSU-CPG-LEP001149
3	11-BOA-13383BrockC10	*Apodemia (Apodemia) mejicanus*		F	USA: AZ, Santa Cruz Co., Peck Canyon, 22-Jul-1990, Jim P. Brock	JimPBrock	
4	NVG-700	*Apodemia (Apodemia) duryi*		M	USA: TX, Brewster Co., Big Bend National Park, Persimmon Gap, 10-Oct-2009, Nick V. Grishin		
5	NVG-7635	*Apodemia (Apodemia) multiplaga*		F	USA: TX, Cameron Co., Brownsville, USH281 at railroad crossing, 24-Oct-1974, W. W. & N. McGuire	TAMU	
6	NVG-17066D09	*Apodemia (Plesioarida) palmerii*			USA: CA, San Bernardino Co., Needles, 2-May-1995, Ken Davenport	CSUC	
7	NVG-17066E05	*Apodemia (Plesioarida) hepburni*			Mexico: Sonora, Rio Sonora Route, vic. Babuiquira, 10-Mar-2003, P. A. Opler	CSUC	
8	NVG-17066D06	*Apodemia (Plesioarida) murphyi*		M	Mexico: Baja California Sur, Bahia Concepcion, Playa Santispac, 26-Mar-7-Apr-2004, Erik B. Runquist	CSUC	CSU-CPG-LEP001455
9	NVG-4514	*Apodemia (Plesioarida) walkeri*		M	USA: TX, Hidalgo Co, 1.5 air mi SE of Relampago, 26.0682, -97.8912, 16-Aug-2015, Qian Cong & Nick V. Grishin		
10	NVG-18048C11	*Apodemia (Plesioarida) hypoglauca*		F	Costa Rica: Guanacaste Prov., 8 km NW Bagaces, 100 m, 28-May-1973, Paul A. Opler	USNM	
11	NVG-17066F02	*Apodemia (Neoapodemia) nais*		M	USA: AZ, Yavapai Co., Prescott, 9-Jul-1993, Ken Davenport	CSUC	
12	NVG-154	*Apodemia (Neoapodemia) chisosensis*		F	USA: TX, Brewster Co., Big Bend National Park, Chisos Basin, 2-May-2004, Nick V. Grishin		
13	NVG-17114H01	*Apodemia (Neoapodemia) ares*		M	USA: NM, Hidalgo Co., Animas Mountains, 4776', 31.7184, -108.8193, 21-Jul-1991, R.W. Holland	CSUC	
14	NVG-8217	*Apodemia (Neoapodemia) zela*		F	USA: AZ, Santa Cruz Co., Coronado N.F., Santa Rita Mts., 25-Mar-2017, Jing Zhang, Qian Cong & Nick V. Grishin		
15	NVG-18045F11	*Apodemia (Neoapodemia) arnacis*		M	Mexico: Oaxaca, 28-29-Jul-1990	USNM	
16	NVG-5245	*Curvie emesia*		F	USA: TX, Hidalgo Co, Los Ebanos, 26.2426, -98.5612, 25-Nov-2015, Nick V. Grishin		
17	NVG-11912	*Curvie emesia*		F	Mexico: Nuevo Leon, Cola de Caballo, 23-Oct-1979, Roy O. Kendall & C. A. Kendall	TAMU	
18	NVG-11914	*Curvie emesia*		F	Mexico: Tamaulipas, nr. El Abra, Paso del Abra, 16-Feb-1974, Roy O. Kendall & C. A. Kendall	TAMU	
19	NVG-18045F01	*Curvie emesia*			Mexico: Morelos, Aug-1979	USNM	
20	NVG-18045F03	*Curvie emesia*			Mexico: Yucatan, 15-Aug-1960	USNM	
21	NVG-18053H01	*Curvie emesia*		M	no data, around 1900, Coll. H. Stichel	ZMHB	1734
22	NVG-18045F09	*Curvie emesia*		F	Costa Rica: ACG, Sector Santa Rosa, 270 m, 10.8170, -85.6437, ecl. 11-Aug-2004	USNM	04-SRNP-13558
23	NVG-18045F10	*Curvie emesia*		M	Costa Rica: ACG, Sector Santa Rosa, 270 m, 10.8170, -85.6437, ecl. 20-Aug-2004	USNM	04-SRNP-13566
24	NVG-19011F11	*Curvie emesia*			Mexico: Oaxaca, around 1990, J. Kemner	TMMC	Kemner #1939
25	NVG-18045C06	*Emesis (Emesis) cereus*			Brazil: Rondonia, 14-25-Nov-1993	USNM	
26	NVG-18045C12	*Emesis (Emesis) neemias*			Brazil: Parana, 19-Mar-1991	USNM	
27	NVG-18045D03	*Emesis (Emesis) orichalceus*			Bolivia, 12-19-Apr-2003	USNM	
28	NVG-18054D07	*Emesis (Emesis) aerigera*	ST	M	Brazil: Sao Paulo, Casa Branca, 1890, Garbe	ZMHB	
29	NVG-18044D03	*Emesis (Emesis) lacrines*			Costa Rica: ACG, Sector Pitilla, 660 m, 10.9928, -85.4294, ecl. 02-Sep-2011	USNM	11-SRNP-32138
30	NVG-18044G07	*Emesis (Emesis) fatimella nobilata*			Panama, 31-Jan-1984	USNM	
31	NVG-18044D05	*Emesis (Mandania) furor*			Costa Rica: ACG, Sector Mundo Nuevo, 570 m, 10.7586, -85.3727, ecl. 03-May-2008	USNM	08-SRNP-55836
32	NVG-18044D09	*Emesis (Mandania) mandana*			Guyana, 21-Feb-10-Mar-1999	USNM	
33	NVG-18044D12	*Emesis (Mandania) russula*			Brazil: Parana, 19-Mar-1991	USNM	
34	NVG-18045A06	*Emesis (Tenedia) melancholica*			Brazil: Rio de Janeiro, 17-Dec-1996	USNM	
35	NVG-18045B01	*Emesis (Tenedia) ocypore*			Peru, 26-Oct-2013	USNM	
36	NVG-18045B08	*Emesis (Tenedia) angularis*			Peru, 10-Nov-2008	USNM	
37	NVG-18045H04	*Emesis (Tenedia) heterochroa*			Peru, 9-10-Nov-2008	USNM	
38	NVG-18044H06	*Emesis (Tenedia) tenedia*			Costa Rica: ACG, Sector Cacao, 1150 m, 10.9592, -85.4682, ecl. 06-Oct-2010	USNM	10-SRNP-36120
39	NVG-18044C08	*Emesis (Tenedia) saturata*			Mexico: Oaxaca, 20-Jul-1990	USNM	
40	NVG-18045G07	*Emesis (Tenedia) cypria*			Venezuela: , 13-Jun-1985	USNM	
41	NVG-18044F08	*Emesis (Tenedia) tegula*			Costa Rica: ACG, Sector El Hacha, 290 m, 11.0322, -85.5278, ecl. 09-Feb-2012	USNM	12-SRNP-20247
42	NVG-18045A03	*Emesis (Tenedia) lupina*		F	Costa Rica, 17-Aug-1977	USNM	
43	NVG-17066E09	*Emesis (Tenedia) phyciodoides*		M	Mexico: Sonora, Pilares, Route 16, km. 301, 15-Sep-2010, Mark Walker	CSUC	
44	NVG-18044G04	*Emesis (Poeasia) poeas*		F	Mexico: Sonora, 23-Sep-1987	USNM	
45	NVG-18045E01	*Emesis (Brimia) brimo*		F	Costa Rica: ACG, Sector San Cristobal, 527 m, 10.9305, -85.3722, ecl. 16-Sep-2008	USNM	08-SRNP-4944
46	NVG-18048A06	*Emesis (Brimia) brimo vimena*	ST	F	Guatemala, prior to 1928	USNM	
47	NVG-18045E06	*Emesis (Brimia) progne*			Bolivia, 12-19-Apr-2003	USNM	
48	NVG-18045D09	*Emesis (Brimia) temesa emesina*			Peru, 7-Nov-2012	USNM	
49	NVG-18045B06	*Emesis (Brimia) satema*			Brazil: Santa Catarina, 28-Oct-1967	USNM	
50	NVG-18044C10	*Emesis (Aphacitis) liodes*			Mexico: San Luis Potosi, 27-Jun-1983	USNM	
51	NVG-18044B05	*Emesis (Aphacitis) aurimna*			Costa Rica: ACG, Sector Cacao, 1220 m, 10.9292, -85.4643, ecl. 01-Jun-2005	USNM	05-SRNP-35299
52	NVG-18044C06	*Emesis (Aphacitis) glaucescens*			Panama, 4-Sep-1982	USNM	
53	NVG-18044B02	*Emesis (Aphacitis) lucinda*			Guyana, 31-Oct-10-Nov-2000	USNM	
54	NVG-18044C02	*Emesis (Aphacitis) fastidiosa*			Brazil: Rio de Janeiro, 27-Oct-1995	USNM	
55	NVG-18044C01	*Emesis (Aphacitis) condigna*			Brazil: Rondonia, 16-Oct-1991	USNM	
56	NVG-18044B12	*Emesis (Aphacitis) castigata*			Peru, 29-Apr-2015	USNM	
57	NVG-18052C08	*Emesis (Aphacitis) opaca*	ST	M	French Guiana: Arouany, 22-Aug-1884	ZMHB	3254
58	NVG-18086H05	*Emesis (Aphacitis) opaca (=diringeri)*	HT	M	French Guiana: Saul, 3-Feb-2003	MNHP	EL63244
59	NVG-18044C04	*Emesis (Aphacitis) eurydice*			Ecuador, 7-Sep-1999	USNM	
60	NVG-18044D02	*Emesis (Aphacitis) spreta*			Guyana, 2-25-Apr-1999	USNM	
61	NVG-18044F11	*Emesis (Aphacitis) vulpina*			Mexico: Yucatan, 15-Aug-1960	USNM	
62	NVG-18044F01	*Emesis (Aphacitis) diogenia*			Peru, 30-Oct-2016	USNM	
63	NVG-18045H06	*Emesis (Aphacitis) heteroclita*			Peru, 10-Oct-1991	USNM	
64	NVG-18086H09	*Emesis (Aphacitis) heteroclita adelpha*	ST	M	Bolivia: Rio Songo, 750 m, prior to 1924, Coll. Fassl	MNHP	EL63248
65	NVG-18054D09	*Xanthosa xanthosa*	ST	F	no data, prior to 1910, Coll. Staudinger	ZMHB	
66	NVG-18053H05	*Sertania guttata guttata*	ST	F	Argentina: Mendosa, prior to 1910, Coll. H. Stichel	ZMHB	3561
67	NVG-5254	*Calephelis perditalis*		F	USA: TX, Cameron Co, E of Brownsville, Palmetto Hill Rd, 27-Nov-2015, Qian Cong & Nick V. Grishin		
68	NVG-4026	*Lasaia sula peninsularis*		M	USA: TX, Cameron Co, Encantada-Ranchito El Calaboz, 26.0211, -97.6167, 12-Jul-2015, Nick V. Grishin		
69	NVG-5148	*Melanis pixe pixe*			USA: TX, Hidalgo Co, Penitas, 26.2262, -98.4361, 15-Nov-2015, Qian Cong & Nick V. Grishin		
70	NVG-10595	*Baeotis zonata zonata*		F	Mexico: San Luis Potosi, 5-Feb-1980	TAMU	
71	NVG-3562	*Caria ino melicerta*		F	USA: TX, Cameron Co, Longoria WMA, 26.3212, -97.8229, 13-Jun-2015, Qian Cong & Nick V. Grishin		
72	NVG-18072D06	*Calydna sturnula*			Costa Rica: ACG, Sector El Hacha, 195 m, 11.0501, -85.5866, ecl. 22-Jan-2012	USNM	12-SRNP-20114
73	NVG-18072F07	*Sarota myrtea*			Costa Rica: ACG, Brasilia, 340 m, 11.0162, -85.3590, ecl. 09-Nov-2013	USNM	13-SRNP-65644
74	NVG-18072F09	*Symmachia rubina*			Costa Rica: ACG, Brasilia, 320 m, 11.0123, -85.3493, ecl. 10-Sep-2012	USNM	12-SRNP-65567
75	NVG-18072F03	*Pachythone gigas*			Costa Rica: ACG, Sector El Hacha, 290 m, 11.0323, -85.5278, ecl. 24-Feb-2014	USNM	14-SRNP-20157
76	NVG-15028B02	*Stalachtis halloweeni*		M	Venezuela: Bolivar, Ptari Tepui, Punto Phelps, 1750 m, 30-Jan-2011, M. Costa	AndrewNeild	
77	NVG-18054E03	*Zabuella tenellus*		M	Argentina: Cosquin, around 1900, Coll. H. Stichel	ZMHB	940
78	NVG-18072G02	*Thisbe lycorias*			Costa Rica: Area de Conservacion Guanacaste (ACG), 2006	USNM	06-SRNP-103224
79	NVG-18072E02	*Calospila argenissa*			Costa Rica: Area de Conservacion Guanacaste (ACG), 2006	USNM	06-SRNP-100631
80	NVG-18072F02	*Nymphidium onaeum*			Costa Rica: ACG, Sector Pitilla, Medrano, 380 m, 11.0160, -85.3805, ecl. 12-Sep-2012	USNM	12-SRNP-72062
81	NVG-18072G01	*Theope publius*			Costa Rica: ACG, Sector Mundo Nuevo, 325 m, 10.7674, -85.4331, ecl. 24-Oct-2013	USNM	13-SRNP-56683
82	NVG-18086H06	*Befrostia lalannei*	HT	M	French Guiana: Route de Kaw, 10-Jan-2004	MNHP	EL63245
83	NVG-18052H02	*Befrostia elegia*	ST	M	Brazil: Rio de Janeiro, prior to 1929	ZMHB	3898
84	NVG-18072E09	*Mesosemia grandis*			Costa Rica: ACG, Sector Cacao, 1185 m, 10.9271, -85.4668, ecl. 22-Mar-2012	USNM	12-SRNP-35041
85	NVG-18048D12	*Dianesia carteri*			Bahamas: N. Andros Island, Stafford Creek, D. J. Harvey, 28-May-1978	USNM	USNMENT 01466534
86	NVG-18072D09	*Eurybia lycisca* DHJ01			Costa Rica: ACG, Sector Rincon Rain Forest, 461 m, 10.9408, -85.3177, ecl. 23-Nov-2013	USNM	13-SRNP-80502
87	NVG-7279	*Euselasia chrysippe*			Costa Rica: ACG, Sector Pitilla, 475 m, 10.9910, -85.3954, ecl. 17-Sep-2011	USNM	11-SRNP-71970
88	NVG-7280	*Styx infernalis*		M	Peru: Cuzco, San Pedro Lodge, Cosnipata Road, 1450 m, 11-Feb-2011, S. Kinyon	USNM	
89	NVG-18071F12	*Abisara bifasciata*			Myanmar, 18-Sep-2001	USNM	
90	NVG-7266	*Curetis bulis*		M	Myanmar: S. Shan State, Min Taing Bin, Ywangan, 31-May-2002, S. Kinyon	USNM	

Diagnostic nucleotide characters mapped to the reference genome of *Calephelis nemesis*

Sequences of nuclear exons with diagnostic characters for the new genera and subgenera are given. The position used as a character state is highlighted in green. Base pair in this position is the one present in the *C. nemesis* reference genome, and may not correspond to the actual base pair in genera and subgenera being diagnosed. A reference sequence for the COI barcode region is given at the end. Many positions of the barcode are used as characters, positions are numbered according to this sequence.

Curvie:
>cne1828.1.1:A1826T | Cadherin-related tumor suppressor
ATGGGGCGGTGGCACGTGGTGCTAGTCGCGCTGGCGCTGTTTACCGGCGCGGGCGCTGGCGCCGCCGCCGAACAGAT
GCAGTCTCGTGCTGTGGACACAGGCGTTGACCTCCGAATACCTGAAGCCCAACCTATTGGAACTGTTGTCGGACGGA
TTCCTATCAAACCTGGTTATACATATCGTTTCAATGAACCACCGAAGGAATTTGTACTTGATCCCGTGTCTGGGGAA
ATCAAGACAAATATTGTACTAGATCGGGAGAACGTGGATAGATATGCATTTGTCGTACTTTCAAGTCAACCGACTTA
TCCAATAGAAGTACGTATACGTGTGATGGACGTTAATGATAATTATCCTGAATTCCCAGAACCAAGCATAGCGGTAC
CTTTTTCTGAAAGTGCGGCATCTGGGACTAAGCTATTGTTAGATGCGGCTACTGATAAAGATCTGGGAGAAAATGGT
ATCACTAACGATTATCGAATAGTGGATGGGGATGATGAAGGTAAATTTCGCTTAAATGTTACTGTGAATCCAAGCGG
TCAAACTTCCTATTTATATTTAGAAACTACAGGTAAACTAGATAGGGAAACTAATGACTTTTACGTTTTAAATATAT
CTGCACGTGATGGTGGAAATCCACCAAAATATGGATATTTGCAAGTTAATGTGTCAATATTAGATGTGAACGATAAT
CCTCCAATATTTGATCAGAGTGATTTCTCAGTTTCTTTAAATGAGAGTGTACCTCCTGGAACTACAGTTCTAAAAGT
AACTGCAACTGACAGTGATTTAGGTGAGAATAGCAAAATTACATACGAGGTTACTGATACTGAAAAACAGTTTGCAG
TGGACCCAGAATCAGGTGTTATTACTACAATAAAAAAATTGAATTGCCCAAAATACTGTAGTAATACAACGTGTAAT
ATGACTTGTGTTTTGACAATTATTGCAAAAGATCATGGTGTACCAAGACAAGATGCTAGAACATATGTAACCGTTAA
TCTAATAGATGCAAATGATCACGATCCCATAATAACATTTACTTACGTACCTCCAACAGCCAATTTTGCGACAGTTG
ATGAAAATGCCAAAAATGGCTCATTAGTTGCAGCTATAACTGTAACGGACCTGGATGCTGGACTCAATGGCATCACT
AGTGTTAGTATAATTGCTGGGAATGAAAACAACCACTTTCGATTGGAAAATTCAAGTAGCGTCTATATCGTACACGT
CAACGGTATTTTAGATAGAGAAGAAATCAGCAAATATAACTTGACTGTTGTAGCTACAGATAGGGGAATACCACCTC
GTACGTCTACTGCATTTTTAGTGATACACGTCAATGATGTTAATGACCATGAACCTGTTTTTGAAAAATCTGAATAT
TCTACTATTTTAAGTGAACTCGCACCATCCGGCACATACGTTGCTGGTATAACTGCTACAGACGAAGACACTGGTGT
TAATGCCGAAATATTTTATGATTTTCATGACGGTAATCAGCAACAGTGGTTTATGATTGATCACATTACTGGATTGG
TAACAACAAAATCTGTTTTGGATCGTGAAGTTCAAGGAACAGTTGAATTAAATGTTTCAGCACGTGACGGCGGCCCC
AATCCTAAATGGGCTTACACTCGCCTAAAAATAACTATTTTAGATGAGAACGACGAAGCACCTTCCTTTCCGCAAAC
ACAAATAAATGCAACGATTTACGAAAATATCAAACCAGTAAAAGAAATCTTAATATTAACAGCATCTGATTATGACC
AAGGTACCAATGGGTCTGTATCTTACTATTTACAACCAAGTACCGAAAGGAGATACCCAAATACATTCTTATTAGAT
GCTATAACTGGTCAATTAAGTACTGTTATAGAATTAGATCGAGAAAGAATACCTTTTTATGAAATATCAGTACTTGC
TAAAGATCAAGGCTTCCCCCCTCAAACTTCTTCTGCTACAGTATTCCTTAAAGTTTTGGATGTCAATGATAATGACC
CAGTTTTTTATCCTCAGCGATACATAGAAAATATCCCTGAAGATTTAGCTCCTGGATCTAAAATAATACAAATCACG
GCATTTGATTATGATGACGAAGACAATGCAAAACTCATTTACAAACTTGAAAGTGGTGGTGATGGCTATTTTGAAGT
AGAACCATGGTCTGGTGAAATATTGTTGCAGAAAGACTTCCGAAAAGCTCCAAAATCATCATATATTTTACGGATAT
CTTGCAAAGATAGAGGCAATAGAAGAGCAATAGAAGATGCAGTTGTTGAAATTTTAAAGAAAACCAGAAGTAGTAGT
TTAGAATTTGAAAGTTACAATGGTTACACTTTTAAAATTGTTGAAGACGATGGTACATCTAAAACTCACAACGGTAG
ATTTGTTGGTAAAGTGATGTCTCGTATGATAAGTGACTCTATTTCTTATTATATTTTAGAAGGTGATCAAAAACATG
CTTTCAAAATTGATGAAAAAAGTGGCATTATTACTACTGAATCTAATATTGATCGAGAAGAGAAAATGAGTTACCAT
TTGAAAGTAATGGCTAAGACAGGATTAGCGTTCGGATTTACAACAGTTAATATATCTGTGCTGGATATAAATGATAA
CTATCCAATTTTTATTGAAGGCAAAGAAGAAATACACATACCTGAAAATATGGCTGTTGGACAAGAAGTATATTTTG
CCAGAGCTACAGATCGTGATTCAGGCTCAAATAGAACAGTTTCTTATGCGTTAACATATAATCCAGATAACAGTTTT
CGTATATCTGCTACAACTGGAGTGATTTATTTGAATAAAGCCATTACAAATGAACCGGGTTCCATTTTATCTTTAGA
GGTTACCGCCACAGATCATGGTAAACCACCACTTGCAACAAAACAGACTATAACAGCAATAGTCGATGATGTCAATG
ATCACACTCCTGTTTTTGATCACACATCTTATGAAACGTCACTATTAGAATCTACTTTAGTAAATACACGCTTTTTT
GCTATTGCTGCAAGCGATGCAGACTTAGGGTCTAATGGCCGAATTAATTATTCAATAATTGAAGGAAATGCTGACGG
AAAATTTGGAATATTTCCTGATGGTTTTCTGTATGTTAAAAGTCCTCTTGATAGAGAAGAAAAAGATTACTATTCAT
TGACAATTACAGCGTCTGACTATGGCAAACCATCAAGATCTTCTCAAGTGCCTGTAGTAATCCATGTTTTAGATGAA
AATGATAATAGTCCACAGTTTACGAACACAACATTCGTTTTTAAAATAAAGGAAAACGAACCTCCTGATACGTTTGT
AGGCAAACTTACTGCAACAGATAAAGATATTGGTCGTAACGCTGAATTGACTTTTACTTTACCAATAATTCAAAACG

ATTTTAGAATTGATTCAAGAAACGGTTTTATTAGAACCACAAAATCTTTTGACAGAGAGAGTTTAGCTCAGAGCACT
GGGCAAAACTACATTACCTTGACAGTTACTGTAAGCGATAACGGTAAAGTTAGACTATCAGACTCCGTAAGAGTAAC
AATTTATATAACCGATGTTAACGACAATGCTCCAATCTTTATACGAACCCCGTATAACGTTGAAGTATCCGAAGGTG
CAGCCGTAGGTGCATCTATAATGAGAGTATATGCAACAGATGCTGATGAAGGTCTGAATGGTGATATTTATTATAAA
TTAATTGGAGGCGATGACACGGAAAAGTTTATGCTCGATGAAGCTACTGGTCAGTTACTGATTAATAAGCCCTTAGA
TCGAGAAACTATGGATCGATATCACCTTACTATAATGGCACATGATTCAGCACAGGCAGTTCGACTTTCATCTACTA
CGACTATAGCTATAGATGTGTTAGATGAAAATGACAATGCCCCGATATTTACACAAACTCAGATGAATATCTCTGTA
TTAGAGACAGAACCGATTAATAAAAAACTGATTCAATTCCATGCGAGTGATTCTGATTTGGGTATTAATAACGAGAT
AACATTTTCTATTTCGTCTGGAAATCGCAAAGAAACATTTTTCATAGACAGTTATTCAGGAGAACTTTTTTTGCATA
AACATCTGGACTACGAAGATTTAACGTCATACATTTTGAATATCACAGCAACTGATAATGGAAATCCTAACTTATCT
TCAAGTATAACATTTATTGTTAATGTCATTGATGCTAATGATAACGCTCCAGTATTCACAAATACAGCGATCGTTAG
ACAAATAAGAGAAGGAATACCAACGCATACTCCAATAGTAACAGTAACTGCGGAAGATCCAGATTCAGGAATAAATG
GCAAAGTATTTTACTCAATAACTCATCAAGATCCTGAAGATAATAGGCGTCATTTTGCCATAAACAACGTAACCGGA
GTTATTCATACCTTACTACCGATTGATAGAGAGAGTATCGACACTTTTAGAATCACTGTAGTTGCATATGATAGAGC
TGAACCGCCTTCTTCGAGACTATCAGCAGAAAAACTTGTAACGGTTATTGTTGAAGACATCAATGATAATGCACCAG
TTTTTGTTTCCATGAATGCTGCAGTAGTGGGCTCAAATAGATTAGGAAGGAGTGCTGGAAGTGGAATATTTATTATG
AATGTGTTAGCCCGGGATCTAGATTCAGGAACCAATGGACTTGTCACATACAAGTTAATACACGGTGGCAACGATAT
GTTTGATTTACACAGAAGTAACGGTGCTTTAAGTTTGCGATATCCACCTGCCATGCCGAATGCTCGATGGAATCTTG
TTATTAAGGCTACAGATGAAGCAGTTTTAAGTGAGCAAAAGAGCACGGAAACTTACTTGACTGTGATAATGGGTGGA
ACTTTATTGGAGGGTATCACATGGACGAACGTAGAATCCGTTTCGGTGCCTGAGAATGAACCAGCCGGGACAGCAGT
GTTAAATATGACTAATAATTATAAATCTGGTTTAGAGTATTATATTGTGAATGTGACTGGTGATCATAAACAAGTGG
ACAGACTATTTGATATAGATTCCTCGTTAGGAATTTTATCTACAGCAGTGCCATTGGATAGAGAAGCTGGAGTTGAA
AAGTATGAAGTTGAAATTTGTGCCGTCTCTTCAAGATCTCCTCTGCAGTCAACTACAACAAAG

>cne3461.1.14:A2393G | Nesprin-1
ATTTGCCGGGATGCCGACCGAAAGCGTGAAGACATGAAATGGTTGGTTCAAACGTTAGACATGCTTTCCAACCACTG
TTCGGACACAGAAGCCCAAGAAGAACAAGTTAAATTAGAAAATCTTATTACAAGATATAAAAATCTCATTCCGACTA
TAGAAATAACAATGATTAAAACAGAAACATATACGAGATGTTACACGTATCGACGAGAAGTACATGAAGTTATATGT
ATTTTAGAAAATGCTAAGCAATATGCACAAGTTAAGCCTGATCCGCAGTCGCTTCAACATGTAGAACAACTTGTCTT
AGAACAACAAGCTGCAGTTCAGAAACTAGATCGTCAACGTCCGTCTGTAATGTCAATGCTTCAGCGAGGCAAGGAAC
TAATAAAAGATGCAAATGCTCCCGCTTTTGTACGTGAAGATGTACGGAATCTTGAAACTGGATGGTCTGCTGCGTAT
GACACCAATACGGAACGACTACACAAACTTCATGACACTCAAAAAGTGTGGTCAAATTACGAATATCAAAAAGAAGA
ACTGTTATCAGATATCAATAAGTTAGAAAGCTATGTTGCTCACCCTGGCTTGGAGCTGGGTGTAACTAACATTGGCC
GTGAGTTACAGGAAACAAAGGCTATTAATATAGATTTAGAAAAAGCCAAATCCGAAAGACTGCCACAGTTACAAAAG
GCCTATGCTGAACTTCAGTCTCTAACTTCAAATAAACCTAAACCAGTTATTGAAAAAGACTTAGAGACGATTGAACG
TAAAATAGGCAGAGTTCATGAAGAGGTTCAGTCTAAAGTTAATCATCTGGAGAAGTTCAATACGGAATGGGTAAAAA
TTGAGCGTAAGTTAGAAGTTGTTCGTGAATGGATTAAAAAAGAAAGCCCTGTATTAATATCACAAATACAAGCAGAA
AACATTACCCCAGAAGAGCGTGTTGAAAAATCCCAGGAACTTCAAAAAGTTATTTCAGAAAAACTAGAAATTATTAA
GAATGTTGCAGACCAAGGGACAAAATTGGCTGTAGAGCACCGCGTACAAGATGCTAACAGACTCAAAGGGGAGGTAG
CTTTATTAGAAAAGATGATGGCTGATTTGCAACGTCATACTGAACATCAGACCAAAGTCGTTGAACACGATTTAGTT
AGTTGGCAGAAGTATCAGAAAGGAGTAGCAGAAATCAAACCATGGATTGAAGAAGCTGAGATTAAGTTAGGTAGTGT
TCCAAAACCAACTACCTTACCTGAAGCACAGCAACTTCAGCAGCAGTCGAGGGCTTTAGTTTCAGATTGTGACAAAC
AACTGACGAACTTACAGATTCTATCGAGTGTTAGCCACCAGTTATCAGGCAAAACTAGTGCTCCCGATGAAGTAGAT
GCTATTCATTCCAGATGGGCTATAGTACATGACTTAGCTGACCAATGGAACAACAAACTTGAAAAACTTGTAACTAA
TTGGGAAAACTTTGAAAGAGATGCTACGAAATTAGAGACTTGGGTCAATGAAGGTGAAAAACTCATAAGTAGTCGAA
GCATAAACTTAGAGACACCGCAATTAGAAAAACTGGATCGGGAGTTAAATAAACTCAAGTCTTTTGGTAATGAAATT
TCTCAACAACAAGCGAAAATTATCAGTCTATCACAAATTTGTGATTCCATATGTCACAACATTCAACCAGAGGGAGC
AGCTGTTTTAAAAAATAGAGTCACCGATATTAAGGACAAAACAAATATGCTTGCTGAATCTGTCAGGTCCAAAGTTA
ATGAATTTTCAGACAAAATTATTGTACGACAAGAGTTTCTTGCTAAATTACATAATTTCGATAATTGGATAAACGAT
TTCAAGAGGAAAACTGAAAGTTACGATCAGATCAGCTCCGACAAAGTGGAACCAACCTTGCAGTCTATGCATGTCCT
ATTACAAGAACATGCTGGAAAAACACCAGAATTCGCTGATATCTACAATGAAGTAAAAAATATGACTTTATCAGCAC
ACCCAGAAGAATCAAAATCTTTGAGTGACACTTACTCAAACATTGCTGAAAATTATCAAGTTATAGAAACAAACATT
CAAAAAAATAAAGGCATACTCCAGAAATGGTCGGATTTGTTAAATTGGTTCGAAGACAACAAACAGCAACTTAACCA
TATTCAGTACCAAATAGAGGGCCCCAAATTGACTCCTGATAGCTATGAAGCACTTCGCCAAGAACTTGTGAATATTT
TGGATAAAATACCTGAATGGAAAAGTAATGTTGTGCTTTTAGACGGTCCTTCAAAAATTAAAGTAACTGATCATAAT
ACGGGTCGAATATTATCACCAACAAGTCTAGTAAAAGAAATAGAAATAAAAGCTGACAATCTCTTAAATTTAGTAAC
TTCAAAAAGGATCTTGTTCAAAAAGTAGGTGCGAGATGGGATAAATTCAATTTACAACACAATGCATTAACTCAAG

TATTGCATCATGTTCAATCGTCACTTAGTGAGATGGCCCAGAAACCAGTACCTTTAACTGAGGGAACCATACAGGAA
ATGTCCGATAAGTTAGACAGCATTGATGCAGAATTAAAGGAAAAACAATCAGTCCGCAAGGAGCTAAGAGAAGAGGG
ACTGCACTTGATGAGGGAAGATCAACCTAATATGGGAACTATACAAAGTGCACTTACTGCTGCTGACAACAGTTGGG
ACCAAGTTGTAAATGTTGTTAGAGATACTAAAAATAAGTATATTCTACTTTCATCTACTTTGCAAGAGTTGAAAAAT
TTTACTCTTGCTTTTGAAAAGGAAATGAGCCGAGCGGAACAATTATATAATGATAGTAAAGAACCACCAAATGACTA
TGTACAGACAGGCCAATTTTTGGATAAATCGAAGAAATGTTATGAAATTTTAAAGCGTTCTAAAGGGTTATTGGACC
AAATGGATCTTATAAAACAATCGGTTTTAAAACAAGCTTCTACACTAGGTGGATTTGATACGAGCCCGTTAGAAAAC
GCTTTCTTAAATTCACAAACACAATGGGAAAAAGTAAACGATGCAGCTATAAAAAGGATACAAGATTTAGAAGCGCA
GTTAATAGTTTGGCGACAAATTGACGATACAAAGAATCAAGTTGTGTCTTGGCTAAGTGAAACTGGACAGAACCTTG
CAAATGCTTGCGATAACTTAGAAATACGATTTGGACAAAATCATCTAACGAAGTATCGAGAAGAATTGCCTATTTAT
GTAGGTCTTAAAAATAGTATTAAAGCAAAATGTGAACAAATAACAAATCTTAATAAAAGCATACCCGTAGGACAAGT
TGCTTCAATTACCGATTATCTCGATAGTGAGTTTCAGGCTTTGGAATCTTTGGCGGAAAATCTTGAGACTGCTGTTT
CATCACTTGAATCTAAAGAAATAGCTTTCAAAGATAAAATTAAGCGTATTTCAGATAAGATAAGCAAAATAAGAGAT
GATATTATAAAATGCGACGATATGTCTGGGGATAATGCTAAGATACTGGATCGCTTGAAAAAATGTCAAACTTGCAA
AACAGAGTTACAAAGTCTCAATGATGAAATTGATAATTTAACACAAAATGTATCTGAAATGAGTGCAAACTATCCAG
GTTTCTATGAGTCATTAGTTCCAAAAGAACTAAGTACATTGCAAAAACGTTTTGAAAGTGTATTAGTTCATGCAAAC
AAAACAGAAACAACTCTATTGACGTTTTTGCAAAAATTATTTACTGATAAATTAAGCATGTTTAACAGAAACTTGAA
GATATTAGATGATAAAACTAGATGGTGTATGCCTGATGCTTCTAATGACAAGTATAATTTAGAAGTAAAAACAGCAG
CTCTTTCTGATGTAGAAAGTGGAATAGCTGATTGTGAATCCAAAGTAAAAGAATTAGTAGATGCTTGTAGAATTTTA
AGTGTTGTAGCTGAACCAATGTGCGCCCAAGAAGCAGCAACACAGACTGAAAAAGCGCAAAAGAATTTAGATGTCCT
TAAAGCTAATTACCAAGAAATCCAAAAACGCTTACAAGAAAGTTCAGAAACTTGGCAAGAATATGAGACTTTATTGG
AAAATGTATCCGAATGGCTAAAAGATAAAGAAAATAAAGTACGACAGGAAGCTGCTACTTTAATTGACCTCAGTTCT
ATAGATCAAAAAATTGCTGAGATAGATGTTGTTGACAAAGAAATTAAAGAATATGGTATTGAAGTTAGAAAGCTTGT
TGATGTTGGAGATAAAGTTATGTCTCATAATCCTGATTCCCGAATAACACAATATATTACACATCTGGAAAGTAGAT
ACCAAACTGTTGGAAAATTCATGGACAATCATTTAAATAGATTAAAGGATCTTAAAAAGGATAATGTAGCATATAAC
AAATCAATAGCAGAGTTCAAGAAGTGGCTACAAAATGCTAATGAAAAAAATTCTGAAGTTGCTGCTATGACGAAACA
TACAAAACCGACATTGGCTGATTTGGAAAACGTTAAGAAATTAAACGAAAGTAAAGACTTAGGTCAGAAATTATTGA
ATAAAGCTATAAGTGATGGAGAAGCTCTTTTCAGTGGTATCACTCCAGAAAATCGTGAGAAAGTCAGGAATGAATTA
AGAACCTTGCGAGATCAGTTTGAAGGATCCGTAGATTCTTTAAATAATGTAATTAAAGACATAGAAACAACAATAAG
TAAACGTTCATCATTAGATGACACATTCAATCAGGTACAAAAATGGATAAGCGACAAGGAAATTGAAATGGGTGAAT
TTAAACTAGCACCAACTCTGCCTGAAAAGAAAGCACAGCTTCATGCTAGCAAAATTCTTAATCAAGAAGTGGAGTTA
TATCAATCTATGTTGTCCCAGTTGACTGAAAAAATTAAACTTATGCCTGATGAGGACGCTGAAAAGGCACTGGCTAG
TATAAATGACAAATACAAGCGTATATCTGATGAGCTTGGTAAAAGAATAAAGGCTTCTGAATCCCACGTGGCCGATC
ATGAAAAATATGTACAAATGTTTGAGAGTGCACGGGACCGCCTTACCCATATAATAGCTGAAAACAGTTTGGTAAGT
TACGGAGTAGTAACTCAAAAAGACGACGTTGACTCTAAAATTTCAGCAATTGAAAAAATCACTTCTAAATCGGAAGA
AATTGATAAAAATCTTGATAAGTGTAAAAAACAGTTGCATGCTGTTTTAGAAACTACGAGTCAAAATGGTCACCCTG
TGCTTATCAGTGAGTTCGAACAACTTAAAACAACGTGGGATCAGTTTGCTTCCCAATGTAAGGAGCATGCCGTTAAA
CTTAAGGAAACTTTGACTCAGTGGAATGCGAGTCAAAAGAAATTAGATGAGTTAGAAGAATGGTTAAAGGTTAAGGA
AAATCAACTAAGGGATCAAAGTTTAAAAAGCGATCTTGAATCTAAAATTGGACATTTACAAAAAGTTAAGGAAATTC
AAACAGAATTAATAACCAGGCAGCCAGAATTTTCAGCTTTAACTAGCAACCAAAATGTTACAGCTGAATCTGAACTT
TCATCAAGAACTTCCAAATTAGCAACTACGTATCACACTCTGAGTAACTTAGTTAATGAAATTATATCTAAATACGA
AGTATTTGTATCTGAGCATCAAATATTTGAAACAGAATATAAGAACATGGAAACTTGGCTCACTAACATGTTGGGAG
ATTTACAAGACCTCAATGAAATTGTAGGTGACTATGCAATATTGCAAGAAAAGCAAAACAAAGCCAAAGAACTTTAT
GACAGTAGAAACAAAAAGACTCCTGCTTTTGAAGAATTCTTAAGTTTAGGAGAAAAACTGTACACTCACACAAGCCC
TGATGGCAGGGAAATAATTCGTCAAAAAATACGCAAAATAAGGAGCTTGTGGGATAGCTTTGGAGATAGTTTCCAAG
AAACAGTAAACAAATTAGATCAGTGTCTCCTACAGTTTTCAGATTTTTCACTAGCTCAAGAACAATTAACCAAATGG
TTAAAAGATGTAGAAAAAGCCATGCAAAAGCATACAGAGCTAAAAGCATCACTTGAGGAAAAGAAAGCCCAACTTCA
GAACCACAAAATCATGCACCAAGAAATTATGGCCCACCAACAACTTGTAGAATCCGTTTGTGATAAAGCACAACAGT
TAGTTGATCAAACCCATGATGCTTCCTTAAATATTTATTTGCAATCCATTAAACAACTTTTCCAAAATATAGTTACT
AAATCACAGAACCTTCAGGATAATTTAGAAGATTGTGTTAAGAAACATGAAGAACTTGTACGTCTCATACAGGCTTA
TAAAGAATGGTTTAGTTCTCAATCAGAAAAGTTATTAGATATAGAAAACGTCATGGGTGAAAAACCTGAAATAGTAA
GAAAACTTCAGACAGCAGTTGCTTTAAAAGACATCGAAAAAGTGGGTTCTAGTAAGTTAGATGAAATTCGAACGTTA
TTTTCTACTGTGAGTAAGAGTACATCAGAAGTAGGAAATGCAGTTATTAAAACAGAAATAGATAATTTACATGATCA
ATTGAGAAAAGGAGTAGATAGTATTGGAACATCAGAACACAAATTAAAGCAAACATTAGAGGTTTGGAATAAATTCG
ATCAAACAATAGAATCTATAACTGACTGGCTAAAAGATATGGAATCTAAATGTAGAGATCAAACGTTATGTTCTATG
TTAGAAGAGAAAGAATCTCAGTTACAAAGATATAACGATCTCAGAAACAATATCTATGCTAAGGAAAAAGAAATAGA
CGTTTTTGTTGATGAATCGCATAGTCTTATTCAGCTTAGTGGCGTAGAGAGACTTAAACCTTTAGTTACTCAAGTCA

ATAACCGGTACCAGCAACTGCATTTAATATCGAAAGAAATTGTAAATCATTGGTCAGAACTAGTAACAGATCACAAA
AAGTATATTCAACTTAAAAATGAATTTGAATTGTGGCTTCGTCCATTAGAAGAACAGTTAGCTCAGATCACGACAAA
AGAAGATAAAATGAGTATTGAAAGTAAAGGGAATAAATTACAAAAACTCTTATTAGAAAAAGATAATGGTGAACATA
AAATAGGAGTTCTCACTAGTGCGGGTGATAAAGTTCTGCCAGAAACTGCAACTAATGGAAGAGAAATAATAAGAGCT
GAAATAAGAGATTTACGAGCGAAATGGGATAAATTCAATGATGGCATTATTCAACAGCAAAAAGATCACGAGTCCCA
AGCCTTGCAATGGTCTAGCTATCAAGATGTGCTCCAGCAGACTTTGACGTGGTTAGATGAAAAAGAAAAACTTATAG
AATCAGAAGAAAAAGCTACGCTTAATACAGCCCAAGAAATAAAATCTAAATTATTAAAAAATAAAACACTGTTACAA
GAAATCAATTCTCACAAACGAGTTATTGAAACAGTAACAGAAAAAGCAAAAAATGTAGCTGGCGTGCATTCTGATAA
ATCTGAAAATGATAAAATGGCAGCCACAATTAAATCAATTTGGGATCGATATAATACTTTGACAAATAGACTAAGTG
CTGTAATTAACAGGCATGAACAATCCTATGAAATTTATCAACAGTTTGCTGATCAACAAAAAGCTCTACAAGATTAT
CAAAAAAATCTATGGGATCGTTTACATGGTTTATCAGATTTTACAGGAAATAAGTCTACATTAATGAATAAATTATC
TAAGATACAAGAGTTATTAGATGCTGTACCAAATGGAAATAACAAATTAAAAATTTTGTCAGATTTAATTGAGAACA
ATGAAACTAAGCTTTCACCGCGTGGCAAAAAAGCTATGACGCGGGAGCTAGGCATATTAAAAGCAGATTTGGAAAAG
TTTAATGCTACTGTACATGATGTCAAGAGAGGCATAGAAGATAAAATTCAGCAATGGATTGAGTTTGATAGCGCTAG
TGATCGACTGCAACATTGGTTAACTGACACAGAAATGAGCCTCAAAACATATACACCAAAAGCTACACTAGAAGAAA
AGATCGATCAACTTAATAAGTAC

>cne1547.14.1:T789C | Titin
ATGACTATAAAAAATAAAAAGACTAAGCAAGAAGTGACTTCGTCGAAGGCTTCGAGTAGTGCGCAGAAGACTAGCTC
GTCGACTTCTGTGCAGAAAAGCAGTACAGTGCAAAAGGTCAGCACATCTGGTAAGAAAGTTCGGTATATCGAAGTAA
AGGTCGAAGAAGATGATCCTCTCATGATAACGGATGTCACCGACCATGCATCCATAAGTGGATCTACCATTTCTGAG
CATTATAATAGCCACCCCCACTACATAATTACGGAAGCTCCTTCCGTCACGGATTTATCCCAAATAAAGTCACATGA
ACATCACGTAAGCGATATCTCACAGTCAAACATCGGTGAGTACGTTTCTGGATCAGTGCTCCAAGAGTCTTCTACTT
CCCACCAACAAAGCTCTACAAGTGAGACGCATCAGTCGTCGTCAACGTCTATTCAACAGTCAGGGTCTAGTCATAGT
CAAACTTTCGATCGCACTATGGACTCCAACGTCAATAATAAAACCCTGAACACTGGTTTTATTGACAACTCAATGCA
TAACGCAAGTGACTCGAGTAATACCTTTTTGTCACGATCTCAGGGAGCAAATGCAACTGACTCCTTTATTCAGTCTG
AGCGTCAAAATTTAATGAACACACAATCAAGGAATAGATCCAGTGTTTCACAAGACAAGATAATAAATAAGCAGATG
TCTTCCACTGATTCGAGAAATGTTAATATTCGCGAGAATGACCGTTTTCAAAACATTCCAAGTACAAGTAGAGATAA
GACTGTCAAGTCAAATACTTTCTAACTTTTACGGGGGTGAAGGAACCCTCGATAAAAATACAAAATCTAAAGAATATA
CCAGTACATATCAAACGAGTGAAAATAAAAGTAGCATGAACACGAAGTCCTCCTCATCTTCGTATGTTGTTGAGATA
GTTGACGGCAAAGAACGTATTATTGATAGTTCAAAAAGAGAATGGGGTGATTCACAGGAAAATGCTTCAAAAGAAGC
ATATGCTAGTGTGAGTGGTACTGATATAAAGCCAGAGACTATTTATAGTAGCCAAGATTATGACATGAGATCAAAGT
ACGACACAGGTAAAGATGGAAGCAAACCTATAAGTGAAATGTCTGTTAAAGAAAATTCTAAAGTCATAAAAGATGGA
AAACAGATTAGCGGTCATGAAAACATGATTTCTCAAAAAGGTAATGTTCTCCATAAAGATTATACTTCAATACAAGA
CAAATCATCATCAGACGAACGTTATCACACAAGTATTGAAAAGGATTCTACTCGTCAAAACATTGATCGTAATTCAA
CCAATATAACGAGCAGAAATACAGTAAGCAACATAACAGACAATAATACAGTAAGCAAAGATACCATAACAAGAAGA
CAGATGAATCAAACGGATTCATCCAACTTTTATGGTTATGATTCAACTATACAAAACCAACTCAAAAAAGTAGGAGA
TATTTTACAAGTGCCTGATACAGAAAATCTAAATTACTCGACGAAAATATTAAAAAGTAACACAAGCAGCGCACAAC
AGGCTTCAACGTCGTCGTATGTTTTTGAAGTTGTAGATGGAAAGCAAAGGGTTGTAGATAGTTCGCATAGGGAATGG
GGTGATAGCAAAGATCATTCAACTTACGAAAAGAGCCATAATTTAAGTGGAACAGGGCTGGAACCACAGCATGAGTA
TTCTCGGCATGTTTTAGATAAGGAATCTTTTTACGATACTGGTAAAGATGGTATACCTAGAAGTGCTACTCAAGTCA
CGGAAGAATCTGCTTTATATAAAAATGGAAAACAAATAGCATTAACTAGCAATACGTATGCTGGTGATTTCACCAAG
AAACCAGCTATTACGGATCATTCCACTGACATAAATGACATTAAAAATAAGCGTATCATAGATCAGACTGATAGAAA
TATTTACTCTGACACTCAAGAGACCACAACTAGAGATACTACATCTAAGACAACTAATGAAAACTATGAAACTATAA
CTAAACAAACACAATCCAATATTACTGACGAGAACTACAAAACCACAACTAGAGATACTACATGTAAAGTAACCGAT
GACAAATTTGTAGACAAGACAACTAGTTTTATTTCAAATGAGAAAGAAAATTTGATAAAAGAGACCACATCTACTAC
TGAAACAAAAGACACTCGTACAACATATGAAAGGAGCACTGGAACTTGGAACGGCAAATTTGTTTATGAAAAAGATG
ATGACCGACCCAAACGTCCAAAAGAAATGTCACCATTTGGAAAACAAGAGCAGCCTAGACATCATTTAAAAAGACAA
GACACAGAGGATAACATTATTTTATCTTCACGTGATATCAGAGATTTCACGTCAATTAGCGATTTACGAAAAATAAT
TGAGTCATCGCAAACCAATAAGGATGTTACTGTTAGTAACAAGAACATTGTTATAAAAAGAAACATAGATGATCGTG
TTCTTAAGGAAATAATTGAAACGGTAAAAAAGTATCCGTTCAAGAGAATCGATAAGGTGACTTTTGGAACAAAAATC
AATGAAGATGTTAAAGATTTATACGAAGGAATCGACACAGAGGAGACTACGGTAGTGAGTACCTCTAGTGGAGATAA
AATCAAAAGATCTTTTGAAATTGACAAAACAAGAAGTCAAATAGAAGTTACGCGTTATATCACTGAAAATGGTATAA
CTAGGAAAGTGACGACTTATGAAGATGCAAAAGAAGACGAGAATGTCAAGACGAATGTTTATGTCGATAATAGTTCG
TTAGACGTCAGAAATTTG

>cne3037.1.5:T844C | Integrator complex subunit 8

CCTGGAATGCAAACAGATACAGCCTACAATCCTTTAAACCAAAACTTTGAGAAGATATTAAGAATGTGTGAAGCAAA
TAGGATGCAAAGTATAAGATATTTAGACAGTATTATAGCATATTATGAAAGCGGACCAGGGAGAAGAGATTCTAAAA
AGATAAAAGTTCCAGTCTTAGAAGCCTTTGTTCACCTTACTGAAGATTCTGATGATATGGATCATGATTGGGAAGCT
GGTGATGTATGTGTCAGCCATTATGAACTAGCCATGCAAATACATTTCGACCTATGTTACAATTACTTCTTTTATGG
TCAACATGATATGGCGAAGCAGCATATTCTTGGATGTCGAGAAAACTATAATTATCTGGAAAATGAAACCAAGTCTT
ATGGCTATGGCTTAGAAAAAGTTCCACAAGGTATACCTTGGGGAGAGTTTTATTATGCAAGTATGACCAAAGATGAT
ATTTTCGGTTATATTAGAGCATTGAATTTAGGATACGAAGTTTTGAATGAAGAGCCCTCTCTCTTACAAAAACTTCA
AGAGTCTATAGCTAATCATTATGCAGGTATTGTAGCCATATTACAAGCTGATAATCTTGCACGAGAAATACCAATGG
TTCACAGAGAAGTTGTAGAACTTGATATACAAGGCTCTGCTTCTAGTGGTGCCTTTACAGTTGCAAGAGATTTACTG
AATAGAGTTTCAGCGCTAAATGCGGTGCGATATGCCCTTGAAGGGGGCATTCCTTCAACCCACCCAGACTTCCTCAA
CAGAGTCAAAACTTTTGGGGCCAAATTTTTTGATCTTCTATTTTGGGCGTTTGCACCAGTCCTAGTATCAGATTTAT
CGGAGGAAGATTGGTATAATTTAAGAGTATTCTTCCTTCATTTGGGAACTTCGCAGTGTAGGATTCCAATTGAGAGG
ATAGACGAGTATCTCAAGCAGTTTATAGGAGATGCAGCTAAAACTATTAGGCGAAAATTGATAAGTGACTCCCACTT
ACGCGACATCCTTGATGAAACGGCAAGTAATGATGATGAAAATATGACTCTTCCCCGAGAGTTACTAACAGATGATT
GGGAAACACCAGATTTTGAATTCAAAACGGTACCAGAACTTGAGATGGGTAGGCTCAAGAAACGCTTAATAGAAGCG
TCAACAGCTGATGATGTACGAATGTGTTTGGTAAAATTAGCCATGATGGCACCTTCATCGCCGCTTTGGAAGTTGAG
TCCTTCATGGAAGCCTCCCGGAGGTTTAGGAAATGCATTAATTTCCTTACCTAGAGGGTTTTTGCAGGACTTTGGGT
ATGTCGTATCAGGTGCTGCTAGGGCACGCGCTGAAGCAGGTCTTGCACGTACGGCATTATCCTTATTGTCAGTATTA
GAAGGTGAAGCTCGAAGCCAGTTGGGTGGCGGTTCAGATCCAAATCTCTACCGCCTTTGTCGTTTATTGTCCTGGGA
GGTGCTATTGCTCCAAGTTAATGTAATGCTAAGCGAGTGGCCCCATCATCGTCTCAACCTGACAGCCTTGGCCAATA
AATGCAAGGCTTGTATTGCCGTTGCTAATTCAGGCGAGAGTATTGTTCCACGT

>cne11073.6.5:A770T | Laminin subunit alpha-2
CTCAAACTAGAAGATGAGCAATATCAAAACAGTGCGGTTATTGAAACTAAAGCATCTGCGAGAACGCTGAGTGACAT
AGTGTCTATATCAGAGTATGACGAACAAGATTTACAAATGCGACGGGCAGACCCAAAACAGAACATAAGTTTTATGG
GACCTCCTGAGGGAGCAAAAATAAACCCAAATAAAACTTTACCCCCCGAGTTACAAAAACCAAACATGAGTTCTCTA
CATGCTGATTATGTGGATCACTTTGATGGAACTAATTTGACACCAAGAGCAGATTCCTTGCCCGTTCACTTAACGTC
AACACAAAACAGAGAAACATATAAAAGAAACGTAAACGAGGTTACTTCAATTGGTATACTAGACAAATTCGTAGATA
GCGGTAAACAGTCTCCAGTTCAGATATTTGACAATTGTTCATTATATCCAAATAGAGATGTGAACGAGAGCAAAAAT
GTTAGCGTTGAACCCAAAAAGATCAATTTCTGTGTGGAACCATCGGATAATAGAACTCCCGATGATGACGAATTTAC
ATCATTAGGAGAGGTAGGTATAACTTTAGACATTAAACAACAGAATTTCCCAGATATTTTATCTCAGTTAAAACATG
AAATCAAAAAATCTAAGATAGAATTAGAAACGTGTAAATCTGAACTTAAAAACGCAGAAGAACAATTATGTGAATTC
CCAGCTCTAAAAGAAGAAGTTGAACAATTGAAAAATGTCTTGGAAAACACTGTTGCTGCAATGGAAAATGACAAAAA
ATTTTATAACAATCAATTGGAAAGCTTCTCCTCAAACAAACAGCTACTAGAGCAAAGACTATTTGAACTAACACAAG
AGGCAAACGAGAAATCAAAAGACCTAAATTTACTTAAAGAAGACATTTTGCGACGCGAAAATATGATTTTAGAGTTA
GCTAAAGAAAAACGGAGCTTAACGAACAAAATAACAGAATTGGAAAATAAAATTGAAGAGTTGCAAGGCAATAACGT
TGTATTAGAAAAATATGAGAAAGAGAATAAACAAATGAGGGAGAAAATAAATGAATTACAGAAACTGGAACAACTAG
TTTCTGACAAAAATCAACAGATAGACAGTCTGAATCAGCATTTAGACAGGTTAGATGATTTGCAGCGTCGATTGAAT
GATAAAAATGAGGAAGTGGAACATTTGAAAAAAGCGTTAAAGGAAAAAGCAAACACTTTGTATCAATTGCAAGAATC
TGTAGGCGAATTGAATAGGGACATTGCTAAGGTAATTCAAGAAAATGACCAGCTCAATGAAGAAAACAAAACTCTCA
AATTAAATCTAAGTAAGCTAGAAAAGGAACAGGAAAATGCTTCAATAAAATTACAAAACAGTGAAGTTGAAGTAGAA
CGCCTTAGTTCATTAAATAATGATTTGAGTTCAAGAATAGAGGAGTTGAGACAGTTAAACGACAAGCTCAAAGATAA
AGAAACTGAAATAGAAATCTTACATGAAGATATCAGTGTATACCATAATGAAATAGCTTTGCTCAAAGAACAGTTAC
AAATGGTATCGCGAAGTCCTTCTCCAAAGAACAAAAGCATTGAAGATAGAAAAACACCTGACAGACAAGCCACTAAT
GATAAAAAACAGTTGGTTAAAATTAAGAAGGAAATATCTCTGCTGCAGCATGAACTTGATTTCAATAGAAAGGAATT
AAACGAT

Poeasia:
>cne703.2.8:A5082G | Protein lava lamp
TTACATAACATGCAACAGAAGTGTGTTGAAATGGAAGACAAATTGGCAGATATGTGTCAACTTCAAAATGAACAAGT
TTGTACGGAAATGAAATCTGTAGAATTGGAAGAGCAAATTGATGAGCTTACATCTTCCAAGAAAGAGTTAGAGCTTG
TTATAGAAAACCTAAGATTAGATAAAGAACATCTAAATAATGCTTTGAAATTGTTACATGACGAAAAAGATGAATTG
TCTCATAAGTTAGAAAACTATATACAAGAAAATATGGAATTGACAGATAAATTAGAAAAGCTAAGTGCTGAAAAAGT
AAGTTCTGCGGAGTCAATAGAAATAGTAGAATCTTTAACAACGCAAGAAAAATTAGAACTTGAAGAATATAATAAAA
GTGTAGAGTCAGATTCAAAAGGAGAGACTCATATACATCATATGACAATATCTGAGGAAAAAGAGAAACTAGTAGAA

GAGGCAATTGAATTAAAAAAGAAACTTGAACTGTTTGCTCAAGAAAGGCAAGAAGTTATGGATAAAATGAATAAAAT
GTGCTCGGAAAATGACGATCTCCATAAGAAATTAACAGAGCTGAAGGAAGAAAGTGACAAACTACGTGACAATATAA
ACATTCTCGTTAACGAAAAACATAAACTTAAACTTGTTAATGAAGAACTTACTCAGAACATTGAAGATTTGAAACAA
GAGAAACTTGAAATGGTAAAAGAAACGGTAGAAGTAGTTAAAAGACCCAGTATTGATGAAAATATTGACACTCCGAT
TGGTGAAACGCCCATTGATGATAAATCTTCAAATGAGAAAGGCATAAACCGGTCTAAATCTATGAAGCAGCTGACTA
AAGAAATCCTTAAGTTAAAGAATACAATTAAAGAACGAGAAAATGAAATTGCTGACTGTCAGATGAAAATATTGTCA
CTAGAAGAACAACAAGAAAAACAAAAAGAATTATTGCAAAATCTTGACTCAAAAGAAGCGACTTTAAGACGACTGAC
AGATGAGAACACACAATTAAAGAAAGATATTGAGGAATTAAAAATAAACAAAGAATCGGATGCAAACATTTTACAGC
TTAAACATGATCATGATAAATTGCAACAAGATTTGCAAAAAATTCACCAAGAATATAACTCGGCTATAAACACACGT
GATTCAAGGATCCATGAATTAGAACAACTTCTATTAGAGTACGAGAAACAAGTGATTACTTATGCCAATTCTTTGCA
ACAAAAAGACAAAGAGATATCTGAGTATATCAATCAAATTACAATGCTTAACGATCTTTCACAAAAGCTTAAATCTA
CGATAGATTTGTTTGAATCTGAAAAAGTAAAAGATCAAAACTTAGAAAGAGGATTAAATAAACAAATAACTATGCAA
CAAAAGACGTTAGCAGAATATGAAGAAAAATTGCGCAATTTAGAAAAAGAAAAAACGCAGTTAGTTTCTTTAAAAAA
TAAGCTGGAAAATAAAAACAATAACATTGAATATGAAATGAAAGCTCTACAGGAAACTATAGCAGAAAGAACAACAC
GTATTAATGATTTAGAAATGCAATCTAAAGAATTTTCGTCGCTTATGGCACAGGCTAAAGAGCGTGATGAAGAAATA
CACCAAATAAAGTTGGAATTAAGAAAAGAATCTATAGAAAATGAAAAATTACGTAAGTTAATTGAACAAAAAGATCT
TGAAATAGATGAACTGAAAAAACGGTTTACAGATGTAACAAATCAACTGAACGCAATTTCTAATGTAGACGTAACTA
AAAGTGAGCAGTATGCAGCATTGGAAAATAAAAATAAAGAGCTAATGGAAAAGTTGAAAAAGTATGCAGTCAGTTTA
AAAAAAAAAACTGCAATGTACTCTGAACTAGAAAAACGGCTAAATGAGGCTCAAGAACAACTTGAAATGAAGAATCA
ACAAGTAGAACAACTCTGTATTCAAGTTGAAATTATTCCAGCGTTGAAAGAAAAACTTAAATATGCTGAAGAAGAGA
TTAATCAATTGCAAACAAAACAAAAAATACTGGAAAATAATTTTGAAGAATTACATAGGGGACAGGCAGAAAACAAG
ACATTAAAAGAAAGCAACATTGGTCTGGAAAATCAAGTTATGAGTCTTAACACACATTTAGATACCTTAAGTATGAA
TTTGGAAGCAGCTTACGACGAAAATAGTACTCTGAAATCAAATATCGAAAATTTGAATAAAAAAATAGTAGAATATG
AAGTAGATCAAAAAAAATAGTTCTAATTTATTAACAAAAATAGCTTGTTTAGAAGCTGAAGTAAATAAAAAACAAATG
CAGCTGACAGAACTCTCTAACGTGCATGACAATCATAGTGAAAAATTAAGTCAAATGCAATTTAGTCATGAGGCCAA
ACTACAGGAACAAGATATGTACATAGAAAGCTTACAATCTGAAATCGAAAAATACAAAAGCCGTATTGGACGGCTTG
AAGAAAGTATATCAAAAATGGAAAATAGCAGATTATCTTTAGAAAGGAAGGCAGATCAATTAGATTATCAGTTACAA
GATAAACAAAAAGCTTATAACGATTATGTCAATCAGGAAGATGAGCTAGTAAGTAGATTAGCTGTTTTAATTGACCA
TGATAAAGTAGTTGAAAAGCAACTACAGGAACTGGAAAATGAAAATAAAGAATTAAAATACAAAATTCAAAATTTCA
AAGAAGACCTGCAAAAAGTTAACGAATTACATTCTGTACTGCAAGAAAAGTATCACGTGTATGAAGTTAAAGCAAAT
AAGTATGATGTAGTTGAGCAAGAATTAAACGACTGTCAAACAAAACTCAGTGATATGGAAATAATGATGAAAAGAGT
AACTAATGAGAATCAGATGACATTGGCTCAAAAAAACAAAGATTTGGAGAATTTGGAATTAGAGTTTACTACGCAGA
TCGAAAGCACAATTAAAGATAAAAAAATATTAAGTGAAAAATATGAAAAAGCATGTGAATATGTAAACAAACTGGAA
TTGGAAATTCAAGAAAGCAGATCTACAATAGAGAATCTTACTAGTAACTGCCGGGACATTGTCTTAGAAAATCAGAA
ACTTTTAGAAGAATCTTCTGTTGCAAAACAATTAGCTTGCCCTGATTACACAGAACAATATATTGGTGAAATAAACA
AGTTACAATCAGTTGTCAATTCTAAAAATGAAGAAATAACTGAAATATCAAATAAAATTCAATCAATACATTCAAAT
AATTTGTCAAAACTTTCAATAGTGGAAGGCAAAGTTATTGAATTAGACGAAAAGCTTAAATTTTCATCTACCGAAAT
TTCAAGGTTGCAAGAAGAAATTAGTAGTTTGAAACATAATAACGATAATCTTCAAATTACTTTAGCCCAAAAAGAAG
ACCAAATAAAACAGCTAATAGAAAATAAAAAACTTGTATTTGAAATGACTATACCAAAAACTGAAGGCATGACAATT
TCATCCACTATAGAGGCATTAGACACTGAACAAAAACCTTGCGATGTATCCAGCATAGAATCACAAATAATTTCTGA
AGGAGATATTGTTGCCCAACGATCAGATAACGTTCAAAAAAGGGAAGAAAAAAAAGGTGAAACTTCCATTACAAACAG
GACCTTCAGGAGAAATAATGGAACCAGCAATTATAATTAAAAAATCTTATATATGTTACAAAAAAGATGAAGAAGTT
GAAGAAACTTCTATTGATCCCTTTAACTCTGATGAAGGTTGGGGTTTGGAAGCTAACATAGAATCTGATGAAGTGAC
ACCTGGAATGGCATACCTTCATAAGCAGATAAATATTTTAAATGAAGCTAATCTAACATTGAAAAAAGATCTTGATG
TTAGTAACAACAAGTTACTCAAAGCTTTAAAAAAACTGAAGGAGTTGAAATCGACGAATGAGATGTTGATGAACGAA
TTAAAGCTCACGAAACAATTATCTCAATCGTCAATGTTAGATATGGCAATCGAAAATGAGTTGTCTAGTAATGTAGA
AGTTTTAGAAAAAAAAGTACAGGATTTAAACACTGAACTTACGAAAGAAAAGCGCGAGAAAGAGGCAATAAAGAAAC
AAAATGAAATTCTTAAAAACGCTAACGACCGTATGATAGAAATGAAAGAAAAAATGGATAACGAAGTAGAATTGTGG
AAATATAACTTCAAACAGGCCAATGATAAATTATCTTCGATGCAATGGGGTGGAGAATCTAAGAGCTATGAATCTAA
AGGAAGTTTTGATGAACCTATCCCAGTTCAAACTACACGCTCCGTGTATGAAGAGGACCTTCTAAAGGTTGAAAAAG
AAAACGACGAATTACAAATATTGGTAGATAATCTTACCACCCAAAATAAAACTTTATCTACTCAGGTAATGCAATCG
AAAGTTGAAATAGATAATCTAAAGCAGCAGTTAGAGGAGCGCCTATCTAAATGTGAGGACTTAAAAAATCAGCTGTT
AGAGTTAGAGAATAAAAACACAGCTCTCCAAAAAAGTTACACTGTTTGTTCCGAAAATATCTCTTTATTGGAAACGC
AATATAATGAATTGAAACACGATAGAGATCAATTAAAATGTAGTTTTGAAGACTGTAAATTGGAGTATGAAACTAAA
AATTCTGAATTAGTTGCCAAAAATAACGATCTGGTAGATGAAATACAATCTCTAAAACATTTACAGTCTGAAAATGC
TTTAAAATTATCGTCTCTTTATTCTGAATTAGAATCTGCTAAGTTAGAATTACAAAATTATAGAGATGACAAAGACA
AAAATATGGATTCAATGATTATAGCAGAAAAGTACAATTCTTTGGAAGAAATTAACCTAAACCTTCAGAAAAGCTTT

GATGAGGCAAATAAAAATAATGAAAAACTAAAACTTGAAAATTCTACACTTTTAGAGAAAATTAACGAATATGAAAA
TAAAATTGATGACTTGACAAAGCAAATAAACCAATTGAATTCTGAAAATGACCAACTGTTGTCAAGCGTTGCAGAAT
TGCGTGCATCTATTTCTAGTGCTCTAGATCAACGAGGTTTCGAAATAGCTGAATTGTGGAAACAACATCTAGCGCAA
AGAGAAAATGAGTTTCAAACGTTAGAACATGATCTTAGAAACCAATTGAATGCTTTTGAAGTGAAATATGAGCAACT
TCTGGAAAACGTACAATCATCAAGTCAGGAGGAAACTAACAAAATTGTTATGACAGAGCAACTCACTTCTTTACAGA
CTAAACTACAGGAAAAAGAAGAACACCTTTTAGATTTACAAAATAAGTATGCTGACGCGATAAGTCAGTTAGATATG
TTACGTTCAGAAATTGAGGACGAACGTCTTATGTTAGAAAATAAGTTACTTGTTCAACAAGAAGAATATGAAAATGT
TATTAAAGATTTAAACTCGAGTCATCAGCTACATTTAAGTGACTATGAAAATAAAAATAAAATTCTCTCCGCTGAAA
TTGATACACTTAAATCAGAAAGTACAAATTTAGATAAACATCTTGAAGAAATAAAACAGCAGTACGAAATAGAGATC
ACAGAATTGAAGAAACAACTTCAACGGAAAGAAGTTGAAATTTTCCAAAAAACCCATGATTTCACTATTTCTTTAAA
CCAGCGAAATGAAGAATTTGAAGTTATAAGAAAACAGTTAGTTGAATATGAGAAAAGGATAGAAGAACTTACATTCG
AAAAAGAATCAGAACTTGCTATGTTGAGGTTAAAAATACATGAACACGGAGAAACTTCCGCGAAAATTCAAAAGGAC
TTGGAGAGTGAAAGAAATACCTTATCAGAAGCCTTAAATGAAAAGATTATAGAGTGCACAAATCTGAATAAATCTAT
TTCTGACTTAAATAAAGAATTAAAAGAATATGCAGATAAAGCTGCGGAAACTCAAATGGTATTAGAAAGTCAAGAAT
TAGAAATTGTTACACTGAAAGACGAAATATCTAGCTTAAGAGACACATTGAGATCGGCTAGTAGCAAGGTAGAAAAG
CATGTAACATTTTCTTCTGATACTAAAAAGAATTTAGAAGAAGAAACCCAAAGTATTTTGAGTAAAGATTTATTAAA
CGCAGTTCCGAAAGCTGAGCTGGATTTGGCCATGTATATGTTGCACCAAAGAGATGTACGTTGTGAAGAGTTAACAC
TAGAATTGACACAGCTATTAGAAGAAAGAGACACTCTACAGTTACGATTGTCGGATAGTTTACGCTCATATGAAGAG
CTTAGATCTCAATTAGCTGGTGCAAATATTGACCAATCTGGTCATGTTTCACCAACCGCATTATCTGACCTATCTGC
TGCTAGTGCTGTTACAGATCGACAATTCGTTGACACACATCGAGGACAAACGTCACGAAGCAGTAGTGTCAGCGATC
CCGACGTAGAAAAGCCGAAATTGCAAGCT

>cne628.1.1:A2634G | Tetratricopeptide repeat protein 37
GTCAAAAGTTTGCTGAAGGAAGCAAGAAAACTTGTCGATGAGAAAAATTATAAGGAAGCTACAGAATGCTGTAAGAG
TATCTTGAGAAAAGATAAACAGAACTACTTGGCTTTAGTGCTTCTTGGTAAATCCTTACAAGATTCTGACCAAGCTC
CATTGGCATATCAAAAGGCCATTGCCTCTAAACCTGATCAACTTCTGGCTTGGCAAGGCCTTGCAAACTATTACGAA
AGAAAAAATGATGAGTCAAGGAGCAAATTGTTCTCTATTTATAATGAAATATTAAAATTACAGATTGATGAAGAAAA
GGCTATAGAAATTGTTAATAAACTTGGACAATTGGGGTGTTTACTTAAAAATAATGAAGCACAGAGCTCTCTAGTTA
ATTTTTTAGATAAAAAACCAAATGAAAATGTTTATAAAGCTGTAGAAACACAATTATTGCAATTAGTCAAAGCAGGT
ATTCCATGCAAAGAAGATGATATTCCGAAACTTGTTGAATTTATTCAACATTTGAACAATTATGGCATGGATGAAAA
CCTTTACATATTACTCGGACAAATCATCCTATCCAAAAAGAACCTCACAAGTGCATTTGAAGAAATATTAAAATTGG
ATATCTTCCCTGCTTCTGTTATGTTTCGAGAATGGTTGTGCAAATATATTTGTACTTATTTTGTACAAGAACAATTT
TTTTCTAATATGAATATTATCACACAATATTACAATGATATTACTGTTGGCATTGAGAATTCTAAATATCCTGGCCT
TTTAAAATCCATGATATTGTTTAATGAAGGTTTATATTTAGATGCTTATAAAATGTGTGTGCCTTTGATAAACTTTC
ATGAAGCTGATATAACTGAAGCAACATTTGTTATTAAATGTACAGTAATGCTTAAGAAGTGGTCTGTTACCCAAAAA
TTAGCTTCGAATTTTTTAATAAAATCCAAAAATCAAGATTTCGTTATTGTTTTGAAGAAATTCTTATTTTTATCTTT
AACTGAACAACATAAATGGAAGCAGGCTATACAGGTTGCAAATGATATTCCACACAGTGCCTTAGATGATAGTGAAA
AAGCTGCTTTAGCAAGATGTTACATAGAAATAAATGAAAATGTCGAAAATGTTTTACAAAATGTAGAATCGACAGAT
TATCATGTACAATTAAAAGTGTTGACATTGATGAAACAAAATAAATATGCGGAGATCATTGAATTGTTAGAAAAACA
AGAAGAAAACTCTTCAAATTTGTTTAACATGGGAAAAGCCTACTGGGAAATGAAGCAATATGATAAAAGCCTAGTGT
ATTTACTTAAAGCTGCAAAATTAGATGCAGATCATGCAGATACATTTTTATATTTAGGGATGTTTTATCAACAATAT
AAAAAAGATTATGAAAAAGCAAAAAAATGTTACGAAAAAGCTTATAGTCTTAATAATTGCGATATTAACACAGTGAA
GAGTTTAAGTTCAGTGTATATAAAGCTACAGCTACTTGATGCTGATTTTGATTTATTGACCAAAACTCTACAAAATA
TTGAAGATGACACATCATGGATAAATTTTAGACTAGGACTTCATTGTTTGAGGAAGAGACAATGGGAAAATGCTATA
ATGCGGTTTAGAGATGTTATTAAACATAACCATAATGATGCAACTGCATTTGAGTGCTTAGCAGATGCTTATTATTC
ACGTGGTTCGTATACGTCAGCTCTTAAGGCATATAATAAGGTTATAGCGTTAGATTCCACAAAAAGCGTCCATTGTT
TAACTAGAATTGGATTAATTCACTCATTACTTACTTCATATGAAGACGCTTTATTAACATTTCAAAAAGTTTTAGAA
ATAGATTCTGGATCATTAATAACATTAAAAGGTATTGCAGAGACCTGGATTAGAATTGCTAAAAAGAAATTTGCTGC
TAAACTTTATGGTAGTGCTAGGGATGCAGCCCAAAATGCTATCAATTATTTGACTGATGCTCTTACAAAAGAGAAAA
AGCATTTATGTTTTTGGAATTTACTAGCAGACATTTTAATAGTAATAACAAAGCTACCTAATAAATATTCTTACGTG
TATATCAAAAACATTATACATGATAACAGTGAAGAAATAGTAAAAAAGGAAACACTTGATATTTTCCCTTTAGCTAT
CTCATGTTATTCACTGATTGCTAAACAGAAACAGCAAGTAGAATCTTATGATTTAGCAAAAACTTATCTTGATTATT
TTCTTGCTTCAAATGATTTAGTCAACTGTCATATAGCATATAGGCTTACAATTGCTTGCATTAAAAAGAAGCCCACT
GTTTGGAGACATTGGAATTTACTTGGAAAAATATGTATGTACCTTAAAAAATATGCTTTAGCACAACACTGTTTTAT
TAAAGCATTATTACTTACTAGAAAATGGTCTGTAGCGAAAATTTGGTGTAATTTGGGAACATTGTATGTTAAAATGA
AAGGTTACAAATTAGCTAATTACTGTTTTTGGCGCGGGCAATCCACTCTTCCTTCCTATCCGCATAGCTGGATAGGC
CAGGCACTAATCGCAGAAGCAATACAAGAAGAGGAGGCCATGGATTTATTTAGACACGCGTCACGGTTAGGCTATCA

CCCAGAAAGTACATTAGGATATGCCGATTGGGTTTGTCGAACTTTAAAGAATATTAAATGTAGTGATTCTGAAACAA
AATATGTCATTGATGGACTCTATGCTATTCCTTATGCCATGGATCTTGTAAACTGGTTTTTGAGTTTTGAACCAAAC
GACTCGTATGCAAATAATGTTGTTGGGATTCTGCAAGAAAGAAGTGGCCTTCTTCATAGTGCCTTACAATCTTACGA
AAAAGCATTAAATTATGTTGATGACAGTAACAGAAATATTGTCCTTTTGAATACTGGACGTATTTACCTAAGGCTTG
AAAGATATGATGATGCTATAAAGACATATAAGGCTATTACGGAAGGAAGTTTGAACTCCGCTTGTGGCCTAGCCTTA
GCTCTTTTAAAGAAATGTGATTACGAGGAAGCTTATTCAGTATATGATACAGCATTCCAATGGCTTTGTAATGATGA
TGAAGAAAAAGCTGATATTTTGGTAGCAATGGCTGGTATATACTACAACTTCAAAGGTGCAGACATGGCTAAAACAC
TGCTATTTCGTAGTATACAAGTTTCACAACAAAAACCTACAGCATACAGTCTTTTTGCTATATGCTCATTTGGAATT
GTCCATTCAGATCAAAGTTTGTCTCGGTTGGCTTTGAGTGAATTAGTTAAATATGAAAAAGATTTTGGATTTGATAT
AGGGTTTCTTAAATCATTTCTGTTCTTTAACAGCGACTTAAAGTTGTCTATCAAAACTATTTCAAATTCTATTTATG
AGCATCCCAATAACGCATTTCTTTGGTACTGTATGGCTCAGTACTGTCTTAATTCAAATGATACAACCAAAATAGCT
AGTGTTTGTGCACAACGAGCATTGTGTTCTGAAAACTTGAATGAATATAACATACATCCAGCTAAAATGCTTTCCAC
TGCTGGTATTGCTGAACATAGCGTGGGAGATATTCTAAAAGCATTTTCGTTAGCTAAAAAGGGAATTCATATGCATC
CATTGCATTCAGATGTATGGGCTCTTTTAATATTTACAATGACTAAAAAGATAAAAGAAAATAAGGCTTGGGTCCTT
AAAACTGCTAATTTTATGAGAAAACAATTGAAAGTTTCAAGAAATTTGAGTAGGTGGATAAATCTTTTAGAGAAAAA
ACTTACAAAGCAAGCTAGT

>cne8205.5.4:A837G | Sentrin-specific protease 1
GTGACATGGATTGAGGAGAAACCAGAAACCAAGAAGTCATCCAAAGTTTACGTAAATGTAGATCGACATGATGTGGA
AAATGATGAAGATGATGAAGTTATATTTGTCAAAGAAATATCCACTCCACCACCACTCAAGCCATACAAATATTTTA
TATCCAAAAATAATCTTGCAGAATCCAGAGATAAAAACATCAAATGTTATAAATTATGTAGGAAACCTAATTCTAAT
TTATCACCTAGAACATATAGTAAGTTTAAAGCGCCTGCGGGTATTACTAAAACATATAAAAGAGCCCCTTCACCAAT
ACCAAAATGGATGCAAGGACAAAACTCGAATCTCTCCCAAGCAAAAAACAAATATCTTAATACAAATGGCAATATGA
GATCAACACTGTCAGAAATTTTTAATTTAGATGAAAAGAGAAATTATCAGGAACTAATAAGAAGAGTTGCTGGGACT
ATGAAACCAGTTTCTTTGACAAAACCATTAGATATTATAAACTTAGCTGAAGACTCGGCTGCCTTTAGACAGACACA
GAAATCTCAAAAGAATGCATTAAATGAAATTAAACTAGTGGAAAAAGGTCTTAGTCTAGAGAAGGATAATGAAATTA
GCAAAGAATATGATCCTATCACAGTCGCATCTATTAACTCTTCTGATTCTGAAATTGAAATTATACCATCAGATGCA
TCCACTACATCATCTATGCGAATTGAACCAATTAACTCATTAAGGGAGTCATTCAGAGATAGAGCAATAACATCTAG
CAATTGGCTCGCTCAAATAGAAAGTAAGTATAGAAAAAAGAAACAGGACACACAAGAAAGGTTAAAAGATGCACATC
GAGAGTCTGAAATAATATCTAAAGTAAATTATGAACAAAAATTAGCACACTTAGAACATAAACTTAAGTATGAGTTA
AGTATACCAGAGAGTCTTATAGAAGAAGCTCCAGCAGTGGTTGAACTGCCACCGCTGACACCGGAACAGGAGAAGTT
GGTAGCCAGGGCTTTGGGGCCCGGCCCACCCGGGCAGCTGCTTATTGAGAAGTTTAATTTGAGAATA

>cne239.4.1:T1275C | Exportin-1
ATGGCAACTTTAGAGCAGCAAGCTTCTAAACTGTTAGATTTTAATCAAAAACTTGATATTAATTTACTCGAAAATAT
CGTGGGGTGTTTTTATTCAACTGTTGGAGATCAACAGCGTGTTGCACAAGAGATTTTAACAGCATTAAAAGAACATC
CAGATGCCTGGACTAGGGTAGACACAATACTTGAATATTCACAGAACCAAGAAACAAAATATTATGCTTTACAAATA
TTAGAACAAGTCATCAAAACGAGATGGAAAGTATTACCAAGAAATCAATGTGAAGGCATAAAAAAATATATCGTAGG
CCTTATAATTAAAAATTCCTCTGATCCTGTGACAATGGAAAATAACAAGGTGTATTTGAAAAAACTTAACATGATTT
TGATTCAAGTGCTAAAAAGAGAGTGGCCTCACAATTGGGAGACATTCATAAGTGATATAGTGGGAGCATCAAAAACG
AATGAAAGCCTTTGCCAAAACAACATGGTGATTTTGAAACTTCTTAGTGAAGAAGTGTTTGTGTTCAGTACAGGCCA
ACTAACGCAAACTAAAGCAAAACATTTGAAGGATACTATGTGCTCAGAGTTTAGTCAAATTTTTACTCTGTGTCAGT
TTGTCCTAGAAAACTCACAAAATGCTCCTCTTGTTGACGCCACATTACACACACTTCTGAGATTCTTAAATTGGATT
CCTTTGGGATACATTTTTGAAATGAAATTGATAAGTACACTTATTTTCAAATTTTTAAATGTTCCAATGTTCAGAAA
TGTTACTCTTAGTTGTTTAACAGAGATAGCTGGTGTAACGGTGAGTAATTATGAAGACCAATTTGTTGCCTTATTGG
TTCAAACTATGGAGCAACTTGAAGCCATGTTGCCTTTGACAACTAACATCCGAGAAGCATATGCTGCAGGAAGGGAT
CAAGAACAAGTATTTATACAAAACCTTGCTATGTTTCTTTGCACTTATTTAAAAGAGCATGGTCAATTAATTGAGCG
AAGAGGCCTTACAAATACACTTATGAATGCACTTCGCTATCTTGTATTAATATCTGAAGTTGAAGAAGTAGAAATAT
TCAAGATTTGTCTAGAGTTTTGGAATGCACTAGTTGCTGATTTGTATAAAATAGCGCCATGCTCCCACTCAACTGGA
ATTTATAGCTTAGGCAAAAGTGTTGGTCGAAAAGTGCTATATGGAGATGTCCTGAGCAGTGTTCGTTATATCATGAT
ATCTAGGATGGCAAAACCAGAAGAAGTTTTAGTTGTAGAAAATGAGAATGGTGAAGTTGTAAGAGAGTTTATGAAAG
ATACTGATTCGATTAATTTGTATAAGAATATGAGGGAAACATTAGTATATTTAACCCATTTAGATTACCAGGATACT
GAGCGTATTATGACAGAGAAGCTTCAGAACCAAGTAAATGGAACTGAATGGTCCTGGAAGAATCTTAACACACTTTG
TTGGGCCATTGGATCCATTTCCGGAGCTATGTTGGAAGAGGATGAAAAACGATTCCTTGTCGTGGTCATCAAAGAAC
TTTTAGGCTTGTGTGAGCAAAAGAAAGGTAAAGATAATAAAGCAATAATTGCTAGCAACATTATGTATGTTGTTGGT
CAATATCCACGCTTTTTGAGAGCTCATTGGAAGTTTTTAAAAACTGTTGTAAATAAACTTTTCGAATTCATGCATGA
AACTCATGACGGTGTTCAAGATATGGCTTGCGATACTTTTATAAAAATTGCTCTTAAATGTCGTCGTCACTTTGTAA

CAACACAAGTTGGTGAAGCATGTCCTTTTATTGAAGAAATATTGAGTACTATAAGCTCTATTATATGCGATTTACAA
ACACTTCAAGTACACACATTTTATGAGGCAGTAGGATATATGATAAGTGCTCAAGTTGATCAGGTAGCTCAAGAACA
GCTTATTGAAAAGTATATGTTGCTGCCTAATCAAGTCTGGGATGATATCATCTCCCAAGCATCACACAATGTTGATA
TTCTAAAAGATCCTGAAGCAGTCAAACAACTGGTTAGTATTTTAAAAACAAATGTGCGTGCTTGTCGAGCACTGGGT
CATCCTTATGTGGTCCAACTTGGAAGAATTTACTTAGACATGCTAAATGTTTATAAAGTTATGTCCGAAAATATCAG
TCAAGCTATAGCACTAAATGGCGTAGTTGTCATCAAACAACCTCTGATCAAAGATATGAGGATTATAAAGAAGGAGA
CTTTAAAATTGATTAGTAATTGGGTTTCCCGTTCCACTGACAATGCAATGGTGTTAGAGAACTTTATTCCTCCTTTG
CTCGATGCTGTACTTTTGGATTATCAGAGGACAGGAGTTCCTGTTGCAAGAGAGCCAGAAGTTCTGTCTTGCATGGC
CGCAATAGTGTACAAGCTTGGTGGTCATATAACATCTGAAGTACCAAAAATCTTTGATGCTGTATTTGAGTGCACAT
TGGAAATGATAAACAAAGATTTTGAAGAATACCCTGAGCACAGAACAGAGTTTTTCTTATTGTTGCAGGCAGTAAAC
ACACATTGTTTCAAAGCATTCCTAAGTATACCACCAGCACAATTTAAGTTGGTGTTAGATTCCATAATATGGGCATT
TAAACATACAATGAGAAATGTTGCAGATACTGGCTTGCAGATTTTATACAGGTTGCTACAAAATGTTGAGCAACATC
CACAGGCAGCACAGAGTTTCTATCAAACATATTTATGTGATATCTTGGAACATGTGTTTAGTGTTGTTACAGACACA
TCCCATGATGCTGGTCTTACAATGCATGCCACAATATTGGCATATATTTTCTCTCTAGTAGAAACTGGTCGAGTTTC
TCTTCCTCTTGGCTGCACTTCTGACAATATTCTATACATACAG

>cne2803.9.1:A528C | Alpha-1,3/1,6-mannosyltransferase ALG2
ATGGTGAAAATATTATTTCTCCACCCAGACCTGGGTATCGGAGGAGCAGAACGTTTGGTTTTAGATGCCGCTTTAGC
ATTTAAAAGTAAAGGCCACGAAGTTTCTTTTTACACTAACCATCATGATTCTTCGCACTGTTTTTCTGAAACACGTG
ATGGAACATTTCCAGTTGCTGTGGTTGGTGATTGGATGCCTCGTTCTATATTTGGGAGGTTTAAAGCCTTATGCGCC
TACACGCGAATGGTTTACGCTGCTATATATCTAGCTTGGTATGTAATACCAGTTGAAGAACCTATGTTAATATTCTG
TGACTTAATATCGTTGTGTATACCGTTTCTTAAAATGGCACGAGGTTCTCATCGAGTTGTCTTTTATTGTCATCATC
CTGACAAGCTACTTTCTTCGCGAGGTGGTTTTCTGAAAAAGTTATATAGGGCACCACTGAATTGGTTAGAAGAATTA
ACCACTGCTTGGGCTGATAAAGTTTTAGTAAATAGTAAGTACACAGCTAGAGTTTATCAGGATGCATTCAAAAAAAT
AAAAGATGTACCAGACATATGCTACCCTTCTATAAATGCTGATTTCTTTAAATCTACTGTGCCTAAAAGCTTAAAAG
AAATAGTTCCAATTGGTGCTGATAAGTTTGTATTTTTATCAATAAATAGGTATGAAAGAAAGAAGAATTTAAAATTA
GCATTAAAAGCATTAGCTGATTTAAAGCAGCACATTTCTAGTTCAGATTGGGATAGAGTTCATTTAATAATGGCTGG
GGGTTTTGATCCCATTAATTTGGAAAATATGGAGCATTTTATTGAGCTCACAGATTTGGCGGCTGAGCTTGATATTG
AAGACAAAGTAACATTCATGAAATCTCCAAAAGATATTGAAAAAGTGTCTCTTTTATACAACTGCAAGGCATTACTG
TACACTCCTTCTAATGAACATTTTGGCATTGTTCCTCTTGAAGCAATGTATTATAGTAAACCGGTCATAGCAGTGAA
TAATGGAGGACCAACCGAAACTATTGTGAATGATGTTACTGGATTCCTATGTGACCCAACAAGTGAATCATTTGCCA
ATGCTATGTATAAATTGCTTACAAACCCAGATTTATGTAAAAGGTATGGAGAGGCAGGAAGAAAGAGATTTGAAACC
AAGTTTTCTTTTGATGCTTTTACCAATCAGCTAGATGGTATCTTAACCAGAGAACGGCTTGCTATTACAGATGCTCG
AGCAATTGATGAACCAAAGAAACAAAAA

Mandania:
>cne3461.1.14:T2709C | Nesprin-1
ATTTGCCGGGATGCCGACCGAAAGCGTGAAGACATGAAATGGTTGGTTCAAACGTTAGACATGCTTTCCAACCACTG
TTCGGACACAGAAGCCCAAGAAGAACAAGTTAAATTAGAAAATCTTATTACAAGATATAAAAATCTCATTCCGACTA
TAGAAATAACAATGATTAAAACAGAAACATATACGAGATGTTACACGTATCGACGAGAAGTACATGAAGTTATATGT
ATTTTAGAAAATGCTAAGCAATATGCACAAGTTAAGCCTGATCCGCAGTCGCTTCAACATGTAGAACAACTTGTCTT
AGAACAACAAGCTGCAGTTCAGAAACTAGATCGTCAACGTCCGTCTGTAATGTCAATGCTTCAGCGAGGCAAGGAAC
TAATAAAAGATGCAAATGCTCCCGCTTTTGTACGTGAAGATGTACGGAATCTTGAAACTGGATGGTCTGCTGCGTAT
GACACCAATACGGAACGACTACACAAACTTCATGACACTCAAAAAGTGTGGTCAAATTACGAATATCAAAAAGAAGA
ACTGTTATCAGATATCAATAAGTTAGAAAGCTATGTTGCTCACCCTGGCTTGGAGCTGGGTGTAACTAACATTGGCC
GTGAGTTACAGGAAACAAAGGCTATTAATATAGATTTAGAAAAAGCCAAATCCGAAAGACTGCCACAGTTACAAAAG
GCCTATGCTGAACTTCAGTCTCTAACTTCAAATAAACCTAAACCAGTTATTGAAAAAGACTTAGAGACGATTGAACG
TAAAATAGGCAGAGTTCATGAAGAGGTTCAGTCTAAAGTTAATCATCTGGAGAAGTTCAATACGGAATGGGTAAAAA
TTGAGCGTAAGTTAGAAGTTGTTCGTGAATGGATTAAAAAAGAAAGCCCTGTATTAATATCACAAATACAAGCAGAA
AACATTACCCCAGAAGAGCGTGTTGAAAAATCCCAGGAACTTCAAAAAGTTATTTCAGAAAAACTAGAAATTATTAA
GAATGTTGCAGACCAAGGGACAAAATTGGCTGTAGAGCACCGCGTACAAGATGCTAACAGACTCAAAGGGGAGGTAG
CTTTATTAGAAAAGATGATGGCTGATTTGCAACGTCATACTGAACATCAGACCAAAGTCGTTGAACACGATTTAGTT
AGTTGGCAGAAGTATCAGAAAGGAGTAGCAGAAATCAAACCATGGATTGAAGAAGCTGAGATTAAGTTAGGTAGTGT
TCCAAAACCAACTACCTTACCTGAAGCACAGCAACTTCAGCAGCAGTCGAGGGCTTTAGTTTCAGATTGTGACAAAC
AACTGACGAACTTACAGATTCTATCGAGTGTTAGCCACCAGTTATCAGGCAAAACTAGTGCTCCCGATGAAGTAGAT

GCTATTCATTCCAGATGGGCTATAGTACATGACTTAGCTGACCAATGGAACAACAAACTTGAAAAACTTGTAACTAA
TTGGGAAAACTTTGAAAGAGATGCTACGAAATTAGAGACTTGGGTCAATGAAGGTGAAAAACTCATAAGTAGTCGAA
GCATAAACTTAGAGACACCGCAATTAGAAAAACTGGATCGGGAGTTAAATAAACTCAAGTCTTTTGGTAATGAAATT
TCTCAACAACAAGCGAAAATTATCAGTCTATCACAAATTTGTGATTCCATATGTCACAACATTCAACCAGAGGGAGC
AGCTGTTTTAAAAAATAGAGTCACCGATATTAAGGACAAAACAAATATGCTTGCTGAATCTGTCAGGTCCAAAGTTA
ATGAATTTTCAGACAAAATTATTGTACGACAAGAGTTTCTTGCTAAATTACATAATTTCGATAATTGGATAAACGAT
TTCAAGAGGAAAACTGAAAGTTACGATCAGATCAGCTCCGACAAAGTGGAACCAACCTTGCAGTCTATGCATGTCCT
ATTACAAGAACATGCTGGAAAAACACCAGAATTCGCTGATATCTACAATGAAGTAAAAAATATGACTTTATCAGCAC
ACCCAGAAGAATCAAAATCTTTGAGTGACACTTACTCAAACATTGCTGAAAATTATCAAGTTATAGAAACAAACATT
CAAAAAAATAAAGGCATACTCCAGAAATGGTCGGATTTGTTAAATTGGTTCGAAGACAACAAACAGCAACTTAACCA
TATTCAGTACCAAATAGAGGGCCCCAAATTGACTCCTGATAGCTATGAAGCACTTCGCCAAGAACTTGTGAATATTT
TGGATAAAATACCTGAATGGAAAAGTAATGTTGTGCTTTTAGACGGTCCTTCAAAAATTAAAGTAACTGATCATAAT
ACGGGTCGAATATTATCACCAACAAGTCTAGTAAAAGAAATAGAAATAAAAGCTGACAATCTCTTAAATTTAGTAAC
TTCAAAAAGGGATCTTGTTCAAAAAGTAGGTGCGAGATGGGATAAATTCAATTTACAACACAATGCATTAACTCAAG
TATTGCATCATGTTCAATCGTCACTTAGTGAGATGGCCCAGAAACCAGTACCTTTAACTGAGGGAACCATACAGGAA
ATGTCCGATAAGTTAGACAGCATTGATGCAGAATTAAAGGAAAAACAATCAGTCCGCAAGGAGCTAAGAGAAGAGGG
ACTGCACTTGATGAGGGAAGATCAACCTAATATGGGAACTATACAAAGTGCACTTACTGCTGCTGACAACAGTTGGG
ACCAAGTTGTAAATGTTGTTAGAGATACTAAAAATAAGTATATTCTACTTTCATCTACTTTGCAAGAGTTGAAAAAT
TTTACTCTTGCTTTTGAAAAGGAAATGAGCCGAGCGGAACAATTATATAATGATAGTAAAGAACCACCAAATGACTA
TGTACAGACAGGCCAATTTTTGGATAAATCGAAGAAATGTTATGAAATTTTAAAGCGTTCTAAAGGGTTATTGGACC
AAATGGATCTTATAAAACAATCGGTTTTAAAACAAGCTTCTACACTAGGTGGATTTGATACGAGCCCGTTAGAAAAC
GCTTTCTTAAATTCACAAACACAATGGGAAAAAGTAAACGATGCAGCTATAAAAAGGATACAAGATTTAGAAGCGCA
GTTAATAGTTTGGCGACAAATTGACGATACAAAGAATCAAGTTGTGTCTTGGCTAAGTGAAACTGGACAGAACCTTG
CAAATGCTTGCGATAACTTAGAAATACGATTTGGACAAAATCATCTAACGAAGTATCGAGAAGAATTGCCTATTTAT
GTAGGTCTTAAAAATAGTATTAAAGCAAAATGTGAACAAATAACAAATCTTAATAAAAGCATACCCGTAGGACAAGT
TGCTTCAATTACCGATTATCTCGATAGTGAGTTTCAGGCTTTGGAATCTTTGGCGGAAAATCTTGAGACTGCTGTTT
CATCACTTGAATCTAAAGAAATAGCTTTCAAAGATAAAATTAAGCGTATTTCAGATAAGATAAGCAAAATAAGAGAT
GATATTATAAAATGCGACGATATGTCTGGGGATAATGCTAAGATACTGGATCGCTTGAAAAAATGTCAAACTTGCAA
AACAGAGTTACAAAGTCTCAATGATGAAATTGATAATTTAACACAAAATGTATCTGAAATGAGTGCAAACTATCCAG
GTTTCTATGAGTCATTAGTTCCAAAAGAACTAAGTACATTGCAAAAACGTTTTGAAAGTGTATTAGTTCATGCAAAC
AAAACAGAAACAACTCTATTGACGTTTTTGCAAAAATTATTTACTGATAAATTAAGCATGTTTAACAGAAACTTGAA
GATATTAGATGATAAAACTAGATGGTGTATGCCTGATGCTTCTAATGACAAGTATAATTTAGAAGTAAAAACAGCAG
CTCTTTCTGATGTAGAAAGTGGAATAGCTGATTGTGAATCCAAAGTAAAAGAATTAGTAGATGCTTGTAGAATTTTA
AGTGTTGTAGCTGAACCAATGTGCGCCCAAGAAGCAGCAACACAGACTGAAAAAGCGCAAAAGAATTTAGATGTCCT
TAAAGCTAATTACCAAGAAATCCAAAAACGCTTACAAGAAAGTTCAGAAACTTGGCAAGAATATGAGACTTTATTGG
AAAATGTATCCGAATGGCTAAAAGATAAAGAAAATAAAGTACGACAGGAAGCTGCTACTTTAATTGACCTCAGTTCT
ATAGATCAAAAAATTGCTGAGATAGATGTTGTTGACAAAGAAATTAAAGAATATGGTATTGAAGTTAGAAAGCTTGT
TGATGTTGGAGATAAAGTTATGTCTCATAATCCTGATTCCCGAATAACACAATATATTACACATCTGGAAAGTAGAT
ACCAAACTGTTGGAAAATTCATGGACAATCATTTAAATAGATTAAAGGATCTTAAAAAGGATAATGTAGCATATAAC
AAATCAATAGCAGAGTTCAAGAAGTGGCTACAAAATGCTAATGAAAAAAATTCTGAAGTTGCTGCTATGACGAAACA
TACAAAACCGACATTGGCTGATTTGGAAAACGTTAAGAAATTAAACGAAAGTAAAGACTTAGGTCAGAAATTATTGA
ATAAAGCTATAAGTGATGGAGAAGCTCTTTTCAGTGGTATCACTCCAGAAAATCGTGAGAAAGTCAGGAATGAATTA
AGAACCTTGCGAGATCAGTTTGAAGGATCCGTAGATTCTTTAAATAATGTAATTAAAGACATAGAAACAACAATAAG
TAAACGTTCATCATTAGATGACACATTCAATCAGGTACAAAAATGGATAAGCGACAAGGAAATTGAAATGGGTGAAT
TTAAACTAGCACCAACTCTGCCTGAAAAGAAAGCACAGCTTCATGCTAGCAAAATTCTTAATCAAGAAGTGGAGTTA
TATCAATCTATGTTGTCCCAGTTGACTGAAAAAATTAAACTTATGCCTGATGAGGACGCTGAAAAGGCACTGGCTAG
TATAAATGACAAATACAAGCGTATATCTGATGAGCTTGGTAAAAGAATAAAGGCTTCTGAATCCCACGTGGCCGATC
ATGAAAAATATGTACAAATGTTTGAGAGTGCACGGGACCGCCTTACCCATATAATAGCTGAAAACAGTTTGGTAAGT
TACGGAGTAGTAACTCAAAAAGACGACGTTGACTCTAAAATTTCAGCAATTGAAAAAATCACTTCTAAATCGGAAGA
AATTGATAAAAATCTTGATAAGTGTAAAAAACAGTTGCATGCTGTTTTAGAAACTACGAGTCAAAATGGTCACCCTG
TGCTTATCAGTGAGTTCGAACAACTTAAAACAACGTGGGATCAGTTTGCTTCCCAATGTAAGGAGCATGCCGTTAAA
CTTAAGGAAACTTTGACTCAGTGGAATGCGAGTCAAAAGAAATTAGATGAGTTAGAAGAATGGTTAAAGGTTAAGGA
AAATCAACTAAGGGATCAAAGTTTAAAAAGCGATCTTGAATCTAAAATTGGACATTTACAAAAAGTTAAGGAAATTC
AAACAGAATTAATAACCAGGCAGCCAGAATTTTCAGCTTTAACTAGCAACCAAAATGTTACAGCTGAATCTGAACTT
TCATCAAGAACTTCCAAATTAGCAACTACGTATCACACTCTGAGTAACTTAGTTAATGAAATTATATCTAAATACGA
AGTATTTGTATCTGAGCATCAAATATTTGAAACAGAATATAAGAACATGGAAACTTGGCTCACTAACATGTTGGGAG
ATTTACAAGACCTCAATGAAATTGTAGGTGACTATGCAATATTGCAAGAAAAGCAAAACAAAGCCAAAGAACTTTAT

GACAGTAGAAACAAAAAGACTCCTGCTTTTGAAGAATTCTTAAGTTTAGGAGAAAAACTGTACACTCACACAAGCCC
TGATGGCAGGGAAATAATTCGTCAAAAAATACGCAAAATAAGGAGCTTGTGGGATAGCTTTGGAGATAGTTTCCAAG
AAACAGTAAACAAATTAGATCAGTGTCTCCTACAGTTTTCAGATTTTTCACTAGCTCAAGAACAATTAACCAAATGG
TTAAAAGATGTAGAAAAAGCCATGCAAAAGCATACAGAGCTAAAAGCATCACTTGAGGAAAAGAAAGCCCAACTTCA
GAACCACAAAATCATGCACCAAGAAATTATGGCCCACCAACAACTTGTAGAATCCGTTTGTGATAAAGCACAACAGT
TAGTTGATCAAACCCATGATGCTTCCTTAAATATTTATTTGCAATCCATTAAACAACTTTTCCAAAATATAGTTACT
AAATCACAGAACCTTCAGGATAATTTAGAAGATTGTGTTAAGAAACATGAAGAACTTGTACGTCTCATACAGGCTTA
TAAAGAATGGTTTAGTTCTCAATCAGAAAAGTTATTAGATATAGAAAACGTCATGGGTGAAAAACCTGAAATAGTAA
GAAAACTTCAGACAGCAGTTGCTTTAAAAGACATCGAAAAAGTGGGTTCTAGTAAGTTAGATGAAATTCGAACGTTA
TTTTCTACTGTGAGTAAGAGTACATCAGAAGTAGGAAATGCAGTTATTAAAACAGAAATAGATAATTTACATGATCA
ATTGAGAAAAGGAGTAGATAGTATTGGAACATCAGAACACAAATTAAAGCAAACATTAGAGGTTTGGAATAAATTCG
ATCAAACAATAGAATCTATAACTGACTGGCTAAAAGATATGGAATCTAAATGTAGAGATCAAACGTTATGTTCTATG
TTAGAAGAGAAAGAATCTCAGTTACAAAGATATAACGATCTCAGAAACAATATCTATGCTAAGGAAAAAGAAATAGA
CGTTTTTGTTGATGAATCGCATAGTCTTATTCAGCTTAGTGGCGTAGAGAGACTTAAACCTTTAGTTACTCAAGTCA
ATAACCGGTACCAGCAACTGCATTTAATATCGAAAGAAATTGTAAATCATTGGTCAGAACTAGTAACAGATCACAAA
AAGTATATTCAACTTAAAAATGAATTTGAATTGTGGCTTCGTCCATTAGAAGAACAGTTAGCTCAGATCACGACAAA
AGAAGATAAAATGAGTATTGAAAGTAAAGGGAATAAATTACAAAAACTCTTATTAGAAAAAGATAATGGTGAACATA
AAATAGGAGTTCTCACTAGTGCGGGTGATAAAGTTCTGCCAGAAACTGCAACTAATGGAAGAGAAATAATAAGAGCT
GAAATAAGAGATTTACGAGCGAAATGGGATAAATTCAATGATGGCATTATTCAACAGCAAAAAGATCACGAGTCCCA
AGCCTTGCAATGGTCTAGCTATCAAGATGTGCTCCAGCAGACTTTGACGTGGTTAGATGAAAAAGAAAAACTTATAG
AATCAGAAGAAAAAGCTACGCTTAATACAGCCCAAGAAATAAAATCTAAATTATTAAAAAATAAAACACTGTTACAA
GAAATCAATTCTCACAAACGAGTTATTGAAACAGTAACAGAAAAAGCAAAAAATGTAGCTGGCGTGCATTCTGATAA
ATCTGAAAATGATAAAATGGCAGCCACAATTAAATCAATTTGGGATCGATATAATACTTTGACAAATAGACTAAGTG
CTGTAATTAACAGGCATGAACAATCCTATGAAATTTATCAACAGTTTGCTGATCAACAAAAAGCTCTACAAGATTAT
CAAAAAAATCTATGGGATCGTTTACATGGTTTATCAGATTTTACAGGAAATAAGTCTACATTAATGAATAAATTATC
TAAGATACAAGAGTTATTAGATGCTGTACCAAATGGAAATAACAAATTAAAAATTTTGTCAGATTTAATTGAGAACA
ATGAAACTAAGCTTTCACCGCGTGGCAAAAAAGCTATGACGCGGGAGCTAGGCATATTAAAAGCAGATTTGGAAAAG
TTTAATGCTACTGTACATGATGTCAAGAGAGGCATAGAAGATAAAATTCAGCAATGGATTGAGTTTGATAGCGCTAG
TGATCGACTGCAACATTGGTTAACTGACACAGAAATGAGCCTCAAAACATATACACCAAAAGCTACACTAGAAGAAA
AGATCGATCAACTTAATAAGTAC

>cne1411.6.4:A2699C | Histone-lysine N-methyltransferase ash1
GATGGATCAAGTAGTGAAATGGAAATCACTCCTCAACTAGTGACAGCGGCGATCCAACGAGCTACAGCTGATTCATC
AGGATCTGAAAACGAATGTTCTAATTCAGATGCTACTCATCAAAATACTTCACATTATGCTTCAAGCTTGTTGCAGC
AATTTGTCGAGCAAACGCAACTACTAAGTAGTACTGCGCCGTTGCCTTCTATCAATACTCCGGCGGCGTGTGGTTTG
ACCACTAATTCTATAGAAGGAGTGGGATCGATAAGTGATTGTGTATTAGGCCAAATAAATAATTTAACAGACATACA
GCCAATCCCTTCAAATTTTTTAAATGGAAATCAACAACATTTAATATCACAACAAACTGAAGCACTTTCTCAAATAA
ATAAAGCCCTTGAAGACCTAACATCAGTTACAGACTCTGTGGATATATCAATACCTAATCCACCGAGTCTAGAGGAT
TGTGTCGATAATAACGATTTTATGAATTTAGATATAACTTCAGGAGGCTCAGAGATCGGAAGTGCAAGTGATCTTTT
AAAAAGTTCGCCCATTACTGTTACAAACAATGATGGCAATTCTGTAAATCGAATAGACTCACAAAAACTAGATAGTA
TCAGTACTAATTCAGTAGAATCTCAAGAGGATGTAAAAAACGTAGTCGTTGAATCAAGAAGAAAAAGAGGGCGGCCT
AGAAAAGTTAGGAAAGTTGAAAGATATGATAATAAAAAATCTGAATCTCAGAAAGAGGACAAATCGGTAGTAAATGT
GATTAATGACTTCCACAATAATAACGATCCGCCCAATGTATCACCCGATTCGGGAATTCTATCAAATCACAACTCAC
CTACACATTCACCATTAAGGCGACACGATGTCGATGAAAGTCATAGTAGACATAGTCGAAAGTCGATTCAAAAAGAA
AATAGTACTATAGAAAGAAAAAGTATGAGGTCAAAATCCTTAACTAGAAATAGAACAAATAGATCTGATTCGAGTGA
GTGTGATTTTCAGAAGAAGAAAATAGAAAATGAGTTTAAACAAATAAAAACAGAAGCACCGTCGCCAGTTCCATTGA
AACAAGAACAACAAAATAAATTTGAGAAACCTAAGAAAAGTGATCACAAACTAGATATTGCTGCTTTAGATAGAATG
TTGTATGCTACTGATAGAGTATTGTATCCACCTCGTAAAAAAGTAGGCCGGAAACCACAGTCAAAAACTAAAGCAAC
TAAAACTACACCAAAAACTTTAAAAGATAAAAACCAATACGATTCCGGTGATAGTGATGATGATTCCTTGCCGTCAA
ACAGATCTGTTTTGTCTGGCGTATATGCAAAAAGAAAAGAACTGAATAGTAAATTATCAAATTTATCAAAGAACAAT
AAAAATAACAAACCTATTGCAAATGCTTGGAGAGATCATCAAAGTGAAAACGAAGCAGCTGTTGATGATCCATTGGA
CCCGACATGGAAACAAATTGACCTTAATCCAAAATACAAAGACATTCTTTCTGGATACAAGAGTGATCATGAATTTA
AACCCTATAAAAGTTGTAGTAAGTTAATAGAATCTGGCTATAAAAGTGACTATGGTTGCAGATCTGGATATAAAACG
GACTACTATCGGTCAGGATATAAAAGCGACCACAAGTCTGGCTATAAAAGTGATAAATCTGGATATAAAACTGATTA
TAGTGTAAGGAGCATGAGGAGGCGAACGCGAAAGTTAAAAAAGACGAGATCAGTTAGAAACCGATCGTACTATAAAA
ATCAAAAACATTTTGTATCAGATCAAGAAATTATGATACTAACAAATAAAACATTTAGCAGTTTAAATTTAGGACAC
AGTTCTAGTGACTCAGAATGTGATACATTTTTACGCAAGTCGAATGCTAGTCCGAAATATGTTAGTGTGTGTACGAA

ATATCCACTACCTTCACGATATAACTATGGTTTTACTAAATTAAATCAAAAACATGTACTAAGGTTAAATGCATCAG
ATCCTTTCGCACCGGGTCCAGTATTTAGTGGTTTGCAACGACCAGTAACTACCTATAATATTTTCAAAATAAATCAT
AACAATAATAACACTTTGTTAAAGTCACCATCTAAAGCAAGCCACGTAGGCAAATCATTGCCTTCTCTAAAACCGGC
ATTTACTCATAAATTTGGAGCGTCTCCAATATTTGTTAATTCCAAAACATCTGCGTCAAAAAAAGACAACGATGTAG
CCAAATATACATCTTCATTATCACCTAAAAGTCGCAAGCAAGTTAAAACTGGACCATCTTCGTCACCCACAACAAAA
AAAGAAAATGCATTGAAGTTGTTACCTAGTATATTTGAACGAGCTAAAAACAGTTATGTTCCAAACAAATCATTGTT
AAGAAACGTGTTTTTGAATTTCAAAAAGCCAAATATCAGACCATCTTTTCACATAAAGAAACACAGAAGAACTGTAG
TACTAGCACCTCCTAAAATCAATTTAAAACCATCTGAGAGGCCTTCACGGCTTACTATTAAAACAGAAGTTAAGCAT
CATAGGCATAGAAGTAAAAGAAGACATCGTTCTAGATCAGTGTCCAAGTGTAGAGACAATTCAGTTGATTTGGTCGA
CCAGAAATTTACTCAAGACATGGATGGATTAATATCTAATTTCATAAAGCTGTGTCAAATTGCTCCTAAGTTAATAA
CAAATTCTACGCAAAGTCAACAGAAAGTAGTCGAGAAGGAAAAACCTTCAACAGAATTACCACCGCCAAAAGTAAAT
AAAAGAGTATCGAAAAAGCGGAAAACATCAGATAATCAAGAAGTTGTGACGCCAACATCTAAAAGAAGACATAAAAA
ACAATTAACTGAATCACAAAATAAAGGTGGAAAAGACACTAATGAACACAAATTACCATTGAAAAAGAGACATTATC
ATATCAATTCTTCTACAAGTAATTCATTAAGTTTGAGTTTGGTCACAACCGAATTTGACGAAAATCTAAAACCCAGT
AGTCCCGATAAATTTTTATGTACGGAAACGGATTGCCTCGGAGAAAATAATGAAGAATCTCCTAAAACTAAATGCCC
ATCTGAAAAAGGAAGAAAAGTAAACGACACCTCAAAAGCGAATGGTCTTGTGAACATGAATGATTTAAAAGCTAATG
TTGAAAGTTCGCCAAGATCATTAAAAACTACTAGCGTCTCAATGAGTGATGACAGCATACTTTTCAAAGATGAAGAA
TTGATTTGCACATCTCCTAAAGATCAGTCAACTTCGCCATCTATTCTTGATGATGTTTTAATAATTAAAAAACCTTC
TCCAGATCAATCTGAATTGTCTAAAAAAATATATGAAACCTCTGAAAAACTTAAAGCTGTTCACAAAATGGTTCACG
ATTTAGAAAAATCTTTACCTAAAACAAAAGACGATTCAAAACCTGAATTATCTAAAATTGAAACAAATCGAATATCT
TCACCAAGATCTGAGACAAAGGCATCTCCGATACGTAATTCTGCTCCTATCGTGACACCCAAGAAACGACACCGATT
AGAAGCTGATAAAGTTATAGCTCATTCGAGTTTAGATCAAGTAGTTCAATCACTATCTAAAAAGTTATCAGAAGACA
AAACCATCAACAATAATATAGTCAAAGATTCAACACAAAGTGATATCAATGATGTTGATAAATCAAATGAACAGTCT
GCAACAAATACTGATGAATCTAACAGCAGCAATTCTACAATAGATCCGCTAAAAAGCATGTCGGCTCGAACCTTGTA
CAAAAGTTCAATACCTCCAGCTCAGAAATCAGAAATTATGACACGGAAAAAGAATAGATTAGAAGGTTTGACTAGTA
ATTTGGTTTCAAAAATAAATCCAAGTGCAGCTACAAAAGTTTTGGACACTCTCCTAAACAATAACATTCGAAAATCA
ATTGAATCTCGAATTTTAGAAAAGGAAAAGAGCATTTCAGAACCTTCAAATAAATCGTCTGAAGAAAAAGAAAGACA
CAAGGAATCCCCTCAGATGAATACAAGGGCTAGTGTTATAAAATCTCCAGTTTCAAAAGCAAAAGTGATGATAGATA
ACAAGAAAGCGAAAGGTGATGTACAGGAAGATTCAAACTTAGTAGTTAATGAGGATAAGCCGACAGGTATTTTTGAA
CCGTCTGTAGATTTAGAAGATCAAATACCCAAATCATCTATTTGTGTCAATAGTATTTTAAGCGATACTGGTAAAAA
CAAAGGGAAATCTAAGGCTGGCGAAAATAGAAGTTCATTGTTGTTACCAATAGACACTGAAGCTGATATACCTCTTG
CATTGATATCGGAGACTACAGATCCTGTAATAAGGCCTAAAAGAGGAGAATCAATTGCTTCTGTGCTATCGGACAAA
ATTCAAGAAACGACCGGTGGGCATAATTTACGACAAGCAAAGAGGAATATAAACTCTGAAAACGAAGAAGCCAATGA
AAAGAAAAAGAAGAAAACATCAAATAGTATTATTAGAGAAAGTAAATTAGTGTTACCAGCAAAAATATTGTTGCCGA
AAATACAAACTGAAAAATTACAAGTCGCTGAACTTAGTAAAAAGACTGCTGTGGCAGAAAATGAGGCTGAGACAGTA
GAATCTAATGAAAAAAGTAATGATTCTTCTAAAAAGAAGCCTAGAAGGCGTAAAGCTATCAATAGAACCGGCTTTCC
AAGTATCAAGAAGAAGAAAAAGAAAGTTGAGTTAAACAATAGTTCTGCTAATGTAACTTCAGATAATCACTTAACAT
CTGAAGATACAGACCACTCCGCATTTGAAAGAGTTCCTAAAGATGGAGAAGCTATGAGTAGTTTCATAGAACGTACT
CACAATACCGGTGCTAAGAAACCAGAACTTAAAGTTGTTTTGAATAAAGATGAAATGCCAAAGCATGGGCGATTGAC
AGTTGTAGCATTGGAGAAACTACAAGGGAAAGAGGTTAAGGACAATAAAGCTGCTGTCCCAATTAAATCAAATGAAA
AGAAAAGCAATATACTAAGAGCACCCGCTTTGCAATTAAAACAAACTAAATTAGATAAAGAAATTAAAAATCATGTT
AATAAATGGGAAGTACTGAGTGAGACGGACAGCATTCCATCATTGACAAGTTCTTTAGGTAATGATCCAGAAGATAG
TATCCCATTGAGTTTACTTAATTTAAAACCTAATGCCAATAAACCTAAATTGGAGAACTTGGAAACATTAAAACGTA
AAACAAGAGCTATGTCACCGTCAAATGAAATTGAAGAAATATTTTCAAAGAGAAAGCATGTAGAGAAAAACAATAAA
GTAGGATTAAGGCCAAAATCAAGTCTAGCTATTTTATATCCTAGTGAAAGGCGATTTACAAGAAGTTGGGATGCAAA
TGACGAAACTAAAAGTAAACCTAAAAAAGTAATACCTAAAAAGTCACTACCTGAAAGTGCTTCCGAAAAAAGTGTTA
AACCCTTAAGTATAGCAGCTAGGAGGAAATCTAGGTCATGTCAAATGAACAAGAAAGTTTTGGAAGTTCAATCAAGT
TCACGGGAAAGTTCATTAGATACTGTAATCAGTCGAAGGCTACCTTCTAAGTCTCGAGAACCTTCTATGGACACATT
GGATAATGATGAAAATGAACCTTTACCATTACACGAGAAAGAGATAGACTTTGAAAAGAGTATAGATACACTCTCTA
AGAATATAATATCCAAGAAGAGAGTTGCCTCTTCACGTGACGAAAGTCCAGCTAGTAGTGTTGATAATCGAGACAAG
CCAGTCGTTTCCAAAAAGAATCCTCGTTTAAGAAAGAAGTTTTTGGTTGCGGGATTGTTCTCAGATTATTACAAAGA
A

>cne1086.2.2:A384G | Tetratricopeptide repeat protein 21B
CAAGTGCAAAATGGAATTAACGATTTGACTCCATTACAGTCTGATAAAGATTTGCAATTAGCTGTTGCTATAGCCCT
CATTTACAGCTATAAACAATTTAATGCTTATGAAAGAGAAGACCTCTACAACTTGGAATCTAAGTTGAAAGAAGACC
GCAAGAATGCAAGTTCTACTTCATTCTACTTTGCTGGTCTTTTTTTATCTTTAGCTGAACGATATGAGAAAGCATCA

GATCATATTAATAAATCGTTAAGGAAAGATCCTAACAATATTGACACTGTTATCCTTAAGGCATGGAATGATATGTA
CATGAATGAACTTACCACTTCAATACCTGATTGTTTCGAGTTAATTCTACATAAGAGTGACAGAAATGTTGAAGCCT
CATTGGGATTAGCAAGATTTAAATACCTGACAAAAGACTACAATGTATCTAATTTGATTCTAGATAAACTAATAGTT
AATAACCCTGGCCAAGTTCTTCCACTTATGGAAAAATTAAAAAATGAACTGGCAGCACAAAAATGGGAATCTGTTTA
TGATGCAGTTGAGAGAATTCTGTCTTTAGATCCTGCCAATACTGAAGCATTAAAAATAAGGATTTATATTGCACTTT
GCAAAAATTCGGATCATATAGAAGCAGTTGATCAACTTAACACATACTACTCAATACTTGAAAATGAAGAAGTCACG
AATGGCTATCAATTTTATAATATTGCCCAATTATTCTCCCGAGCCTGTGGACGGTCAAGTGCAGTTCTTTCACAAGT
TTACAGATTTGCACAATATGCTTCAGAACTTTACCCAAACAATGTTGATTATTTAAGTGAAGTTGGCTACCAGTGTA
TACTGCAAGGGAAATATAAAGATGCATTAAATTTCTTCAAAGCTGCTTCTAAAATAGATAGTAACTCAATAATTACT
CTATGCGGCTTAACACTTTGTCAGATGTTTGAAAATGGCCCAACAGACCAGATTTCACAACAAGTTGAGCTCTTGTT
TGAAATGCAGGGAACACAGAAATTTCCCATGTTATACTTATTATCAGCTCAGTTGAATAGTAAAAATAATAACGCTG
TATCATTTCTAAACAAAGCAATAGAAGCCCAATTGTCACAAGTTGAAAAACTTCCATTTAGCATGTCTTATATACAC
CAGTTAGATCCAGATTTCATAATGGAAGTTTACAAAGAGTACAAAAAACATTTACCTAAAAAGCCTTTTGTAATTAC
GGGTTACTTGCTATATTTACAAAATGATAGTAGTGATTTGGCTGCTGCTTGCATTAATATTTTGACTACAGTAGTCA
ATGCTTGTCCAGGACTTATCCCTGCATTATATGAGTTGGCTAAAATAAAATTTTTATTTGGTTTTGCAAATGAAGCA
CAGAAACTGGCTCAACAAATAGGAGATCTTGATAATACCCATGCTGGTTGTCAGATGTTATTAGCACAAATTTATAT
ACAGCAACAAGCATTTAAAAAAGCTGACCAAAGTCTAGAAACTTGTTTAAGTTATAATTTTAAAGTGAAAGACAGTG
CTATGTATAATTTATTAAAGGGTGTTATTCTGAAAAATGACAACCAACTGCATGATGCCTTGTCACATTTTAATAGC
AGCCTACAAATGCTGACAAGCAAAAGTAATGTGCATATGAACAAGCAATATGATGCCGATTTGAATATAATTGACAA
AGCTACATTATATTTACAAATAATTGAATTACAAACAACTTTGGGTTTCTTGGGCGAAGCTGGAAAAATGATG

>cne123.6.3:T427C | Leucine-rich repeat-containing G-protein coupled receptor 4
ACCAGTCTCAACGTTTCATACAATGAAATAGCCACTTTTCCGGAAAGCTTAAAGAAATTTTACAATTTATTAACATT
TGATATAAGTGGAAATCAGCTGAGAAATATACCAGAAGATGCACTACAAAATTTAACTTCATTAGAAGTTTTAATTC
TATCCCGAAACTATTTAGAAGAGTGGCTGAATATTAACCCTAATGTCTTGCTGCAACCAGCAACTAGCTTAAAAACT
CTAGATTTATCTCAAAATAAATTTAAATCATTATCAAATCTGGCGAATCAAGAACTCTTAATTAGCTCATCCTTAGA
AAATCTAATTTTAGATAATTGTGAAATAAATTCTGTATACGGAAGATCACCGCTTAGTGGCCTCAGCAACATAAGAG
TGTTAAAAATTAATAGCAACCCAATAACACAATTCCGAAAATTAATATCACCTACTTTGCGAAGCCTTTATATGAGT
AATTGTCAACTCAGTTATATAAATCATGACGAACTTTCTTATTTACCGTCATTAGTATATTTAAAGATGTCACACAA
CTTTCGCTTAGAGTTATCTACTAACACCAATATAATATTTTCAGATTCCCTAAAATTTCTAGATATATCCTATTGTA
ATGTATTTCAGCCAAATTTAAGAGGATTTCCTAATCTTAGAAGAGCTATAATCAATCACAATATGATACGGTATTTA
AAAAGCAATGAATTTACAAATAATTCAAAATTAGAGTATTTAGATTTATCGTACAATAATATAGGATCTATAAAAAG
TGACACTTTTCGTGGTCTTAGTATGATAAGATATTTAGATTTGTCTTGGAATGAAATAGCCCAAATACCAGAAGATA
GCCTTTTGGTCATGCCTTCACTGACGCAGTTAAAGCTTTCAAGAAATTACTTAACAAGAGTTGGTCACCTCAAATCA
ACATCTTTATCAATTTTGGATCTTAGTTCCTGTGAAATAAATACAGTGGGTAAAGACTCACTTGAAGGTTTACCTTC
AGTCACAGACTTAGATTTATCTCACAACCTTTTATCACATATTCCAGATAGTATTTCATCAAACACATTGAAAAATT
TAAATATAAATTATAATAGAGTCAGTTCTGTTAGTAATTACACTTTCTTTATGCTACCACGACTTACCAGTTTAAGC
GTTGTCGGCAACCGCTTTACAATGATTTGGAACAAGTCTTATTTTGATTCCAATTTATATTTGGAAAGCCTTGATTT
AAGCGACAATATGTGGCGTTGCGATTGTTCGGATGACAATATGTTCGATTTCTATGAATATGTGACATTAGAACCAA
ATAAAAAAGAAGAATCATATAATCTTATATGTAACAGTCCCATTCAGGTGAACGGTCAAAGTTGGTTAGAGGCGTGT
TACTTTGTATGGCACCCCAATGAAAAAACAGCTAACGTAGACAGTGTTATTTGGTTCATCATAGTTATGATAATTGG
ATTAGCGTTATGTCTCATATTAGTTAATGCTATTAGGAAATCTATGAAGAAA

>cne3461.1.14:T2338C | Nesprin-1
ATTTGCCGGGATGCCGACCGAAAGCGTGAAGACATGAAATGGTTGGTTCAAACGTTAGACATGCTTTCCAACCACTG
TTCGGACACAGAAGCCCAAGAAGAACAAGTTAAATTAGAAAATCTTATTACAAGATATAAAAATCTCATTCCGACTA
TAGAAATAACAATGATTAAAACAGAAACATATACGAGATGTTACACGTATCGACGAGAAGTACATGAAGTTATATGT
ATTTTAGAAAATGCTAAGCAATATGCACAAGTTAAGCCTGATCCGCAGTCGCTTCAACATGTAGAACAACTTGTCTT
AGAACAACAAGCTGCAGTTCAGAAACTAGATCGTCAACGTCCGTCTGTAATGTCAATGCTTCAGCGAGGCAAGGAAC
TAATAAAAGATGCAAATGCTCCCGCTTTTGTACGTGAAGATGTACGGAATCTTGAAACTGGATGGTCTGCTGCGTAT
GACACCAATACGGAACGACTACACAAACTTCATGACACTCAAAAAGTGTGGTCAAATTACGAATATCAAAAAGAAGA
ACTGTTATCAGATATCAATAAGTTAGAAAGCTATGTTGCTCACCCTGGCTTGGAGCTGGGTGTAACTAACATTGGCC
GTGAGTTACAGGAAACAAAGGCTATTAATATAGATTTAGAAAAAGCCAAATCCGAAAGACTGCCACAGTTACAAAAG
GCCTATGCTGAACTTCAGTCTCTAACTTCAAATAAACCTAAACCAGTTATTGAAAAAGACTTAGAGACGATTGAACG
TAAAATAGGCAGAGTTCATGAAGAGGTTCAGTCTAAAGTTAATCATCTGGAGAAGTTCAATACGGAATGGGTAAAAA
TTGAGCGTAAGTTAGAAGTTGTTCGTGAATGGATTAAAAAAGAAAGCCCTGTATTAATATCACAAATACAAGCAGAA

AACATTACCCCAGAAGAGCGTGTTGAAAAATCCCAGGAACTTCAAAAAGTTATTTCAGAAAAACTAGAAATTATTAA
GAATGTTGCAGACCAAGGGACAAAATTGGCTGTAGAGCACCGCGTACAAGATGCTAACAGACTCAAAGGGGAGGTAG
CTTTATTAGAAAAGATGATGGCTGATTTGCAACGTCATACTGAACATCAGACCAAAGTCGTTGAACACGATTTAGTT
AGTTGGCAGAAGTATCAGAAAGGAGTAGCAGAAATCAAACCATGGATTGAAGAAGCTGAGATTAAGTTAGGTAGTGT
TCCAAAACCAACTACCTTACCTGAAGCACAGCAACTTCAGCAGCAGTCGAGGGCTTTAGTTTCAGATTGTGACAAAC
AACTGACGAACTTACAGATTCTATCGAGTGTTAGCCACCAGTTATCAGGCAAAACTAGTGCTCCCGATGAAGTAGAT
GCTATTCATTCCAGATGGGCTATAGTACATGACTTAGCTGACCAATGGAACAACAAACTTGAAAAACTTGTAACTAA
TTGGGAAAACTTTGAAAGAGATGCTACGAAATTAGAGACTTGGGTCAATGAAGGTGAAAAACTCATAAGTAGTCGAA
GCATAAACTTAGAGACACCGCAATTAGAAAAACTGGATCGGGAGTTAAATAAACTCAAGTCTTTTGGTAATGAAATT
TCTCAACAACAAGCGAAAATTATCAGTCTATCACAAATTTGTGATTCCATATGTCACAACATTCAACCAGAGGGAGC
AGCTGTTTTAAAAAATAGAGTCACCGATATTAAGGACAAAACAAATATGCTTGCTGAATCTGTCAGGTCCAAAGTTA
ATGAATTTTCAGACAAAATTATTGTACGACAAGAGTTTCTTGCTAAATTACATAATTTCGATAATTGGATAAACGAT
TTCAAGAGGAAAACTGAAAGTTACGATCAGATCAGCTCCGACAAAGTGGAACCAACCTTGCAGTCTATGCATGTCCT
ATTACAAGAACATGCTGGAAAAACACCAGAATTCGCTGATATCTACAATGAAGTAAAAAATATGACTTTATCAGCAC
ACCCAGAAGAATCAAAATCTTTGAGTGACACTTACTCAAACATTGCTGAAAATTATCAAGTTATAGAAACAAACATT
CAAAAAAATAAAGGCATACTCCAGAAATGGTCGGATTTGTTAAATTGGTTCGAAGACAACAAACAGCAACTTAACCA
TATTCAGTACCAAATAGAGGGCCCCAAATTGACTCCTGATAGCTATGAAGCACTTCGCCAAGAACTTGTGAATATTT
TGGATAAAATACCTGAATGGAAAAGTAATGTTGTGCTTTTAGACGGTCCTTCAAAAATTAAAGTAACTGATCATAAT
ACGGGTCGAATATTATCACCAACAAGTCCTAGTAAAAGAAATAGAAATAAAAGCTGACAATCTCTTAAATTTAGTAAC
TTCAAAAAGGGATCTTGTTCAAAAAGTAGGTGCGAGATGGGATAAATTCAATTTACAACACAATGCATTAACTCAAG
TATTGCATCATGTTCAATCGTCACTTAGTGAGATGGCCCAGAAACCAGTACCTTTAACTGAGGGAACCATACAGGAA
ATGTCCGATAAGTTAGACAGCATTGATGCAGAATTAAAGGAAAAACAATCAGTCCGCAAGGAGCTAAGAGAAGAGGG
ACTGCACTTGATGAGGGAAGATCAACCTAATATGGGAACTATACAAAGTGCACTTACTGCTGCTGACAACAGTTGGG
ACCAAGTTGTAAATGTTGTTAGAGATACTAAAAATAAGTATATTCTACTTTCATCTACTTTGCAAGAGTTGAAAAAT
TTTACTCTTGCTTTTGAAAAGGAAATGAGCCGAGCGGAACAATTATATAATGATAGTAAAGAACCACCAAATGACTA
TGTACAGACAGGCCAATTTTTGGATAAATCGAAGAAATGTTATGAAATTTTAAAGCGTTCTAAAGGGTTATTGGACC
AAATGGATCTTATAAAACAATCGGTTTTAAAACAAGCTTCTACACTAGGTGGATTTGATACGAGCCCGTTAGAAAAC
GCTTTCTTAAATTCACAAACACAATGGGAAAAAGTAAACGATGCAGCTATAAAAAGGATACAAGATTTAGAAGCGCA
GTTAATAGTTTGGCGACAAATTGACGATACAAAGAATCAAGTTGTGTCTTGGCTAAGTGAAACTGGACAGAACCTTG
CAAATGCTTGCGATAACTTAGAAATACGATTTGGACAAAATCATCTAACGAAGTATCGAGAAGAATTGCCTATTTAT
GTAGGTCTTAAAAATAGTATTAAAGCAAAATGTGAACAAATAACAAATCTTAATAAAAGCATACCCGTAGGACAAGT
TGCTTCAATTACCGATTATCTCGATAGTGAGTTTCAGGCTTTGGAATCTTTGGCGGAAAATCTTGAGACTGCTGTTT
CATCACTTGAATCTAAAGAAATAGCTTTCAAAGATAAAATTAAGCGTATTTCAGATAAGATAAGCAAAATAAGAGAT
GATATTATAAAATGCGACGATATGTCTGGGGATAATGCTAAGATACTGGATCGCTTGAAAAAATGTCAAACTTGCAA
AACAGAGTTACAAAGTCTCAATGATGAAATTGATAATTTAACACAAAATGTATCTGAAATGAGTGCAAACTATCCAG
GTTTCTATGAGTCATTAGTTCCAAAAGAACTAAGTACATTGCAAAAACGTTTTGAAAGTGTATTAGTTCATGCAAAC
AAAACAGAAACAACTCTATTGACGTTTTTGCAAAAATTATTTACTGATAAATTAAGCATGTTTAACAGAAACTTGAA
GATATTAGATGATAAAACTAGATGGTGTATGCCTGATGCTTCTAATGACAAGTATAATTTAGAAGTAAAAACAGCAG
CTCTTTCTGATGTAGAAAGTGGAATAGCTGATTGTGAATCCAAAGTAAAAGAATTAGTAGATGCTTGTAGAATTTTA
AGTGTTGTAGCTGAACCAATGTGCGCCCAAGAAGCAGCAACACAGACTGAAAAAGCGCAAAAGAATTTAGATGTCCT
TAAAGCTAATTACCAAGAAATCCAAAAACGCTTACAAGAAAGTTCAGAAACTTGGCAAGAATATGAGACTTTATTGG
AAAATGTATCCGAATGGCTAAAAGATAAAGAAAATAAAGTACGACAGGAAGCTGCTACTTTAATTGACCTCAGTTCT
ATAGATCAAAAAAATTGCTGAGATAGATGTTGTTGACAAAGAAATTAAAGAATATGGTATTGAAGTTAGAAAGCTTGT
TGATGTTGGAGATAAAGTTATGTCTCATAATCCTGATTCCCGAATAACACAATATATTACACATCTGGAAAGTAGAT
ACCAAACTGTTGGAAAATTCATGGACAATCATTTAAATAGATTAAAGGATCTTAAAAAGGATAATGTAGCATATAAC
AAATCAATAGCAGAGTTCAAGAAGTGGCTACAAAATGCTAATGAAAAAAATTCTGAAGTTGCTGCTATGACGAAACA
TACAAAACCGACATTGGCTGATTTGGAAAACGTTAAGAAATTAAACGAAAGTAAAGACTTAGGTCAGAAATTATTGA
ATAAAGCTATAAGTGATGGAGAAGCTCTTTTCAGTGGTATCACTCCAGAAAATCGTGAGAAAGTCAGGAATGAATTA
AGAACCTTGCGAGATCAGTTTGAAGGATCCGTAGATTCTTTAAATAATGTAATTAAAGACATAGAAACAACAATAAG
TAAACGTTCATCATTAGATGACACATTCAATCAGGTACAAAAATGGATAAGCGACAAGGAAATTGAAATGGGTGAAT
TTAAACTAGCACCAACTCTGCCTGAAAAGAAAGCACAGCTTCATGCTAGCAAAATTCTTAATCAAGAAGTGGAGTTA
TATCAATCTATGTTGTCCCAGTTGACTGAAAAAATTAAACTTATGCCTGATGAGGACGCTGAAAAGGCACTGGCTAG
TATAAATGACAAATACAAGCGTATATCTGATGAGCTTGGTAAAAGAATAAAGGCTTCTGAATCCCACGTGGCCGATC
ATGAAAAATATGTACAAATGTTTGAGAGTGCACGGGACCGCCTTACCCATATAATAGCTGAAAACAGTTTGGTAAGT
TACGGAGTAGTAACTCAAAAAGACGACGTTGACTCTAAAATTTCAGCAATTGAAAAAATCACTTCTAAATCGGAAGA
AATTGATAAAAATCTTGATAAGTGTAAAAAACAGTTGCATGCTGTTTTAGAAACTACGAGTCAAAATGGTCACCCTG
TGCTTATCAGTGAGTTCGAACAACTTAAAACAACGTGGGATCAGTTTGCTTCCCAATGTAAGGAGCATGCCGTTAAA

CTTAAGGAAACTTTGACTCAGTGGAATGCGAGTCAAAAGAAATTAGATGAGTTAGAAGAATGGTTAAAGGTTAAGGA
AAATCAACTAAGGGATCAAAGTTTAAAAAGCGATCTTGAATCTAAAATTGGACATTTACAAAAAGTTAAGGAAATTC
AAACAGAATTAATAACCAGGCAGCCAGAATTTTCAGCTTTAACTAGCAACCAAAATGTTACAGCTGAATCTGAACTT
TCATCAAGAACTTCCAAATTAGCAACTACGTATCACACTCTGAGTAACTTAGTTAATGAAATTATATCTAAATACGA
AGTATTTGTATCTGAGCATCAAATATTTGAAACAGAATATAAGAACATGGAAACTTGGCTCACTAACATGTTGGGAG
ATTTACAAGACCTCAATGAAATTGTAGGTGACTATGCAATATTGCAAGAAAAGCAAAACAAAGCCAAAGAACTTTAT
GACAGTAGAAACAAAAAGACTCCTGCTTTTGAAGAATTCTTAAGTTTAGGAGAAAAACTGTACACTCACACAAGCCC
TGATGGCAGGGAAATAATTCGTCAAAAAATACGCAAAATAAGGAGCTTGTGGGATAGCTTTGGAGATAGTTTCCAAG
AAACAGTAAACAAATTAGATCAGTGTCTCCTACAGTTTTCAGATTTTTCACTAGCTCAAGAACAATTAACCAAATGG
TTAAAAGATGTAGAAAAAGCCATGCAAAAGCATACAGAGCTAAAAGCATCACTTGAGGAAAAGAAAGCCCAACTTCA
GAACCACAAAATCATGCACCAAGAAATTATGGCCCACCAACAACTTGTAGAATCCGTTTGTGATAAAGCACAACAGT
TAGTTGATCAAACCCATGATGCTTCCTTAAATATTTATTTGCAATCCATTAAACAACTTTTCCAAAATATAGTTACT
AAATCACAGAACCTTCAGGATAATTTAGAAGATTGTGTTAAGAAACATGAAGAACTTGTACGTCTCATACAGGCTTA
TAAAGAATGGTTTAGTTCTCAATCAGAAAAGTTATTAGATATAGAAAACGTCATGGGTGAAAAACCTGAAATAGTAA
GAAAACTTCAGACAGCAGTTGCTTTAAAAGACATCGAAAAAGTGGGTTCTAGTAAGTTAGATGAAATTCGAACGTTA
TTTTCTACTGTGAGTAAGAGTACATCAGAAGTAGGAAATGCAGTTATTAAAACAGAAATAGATAATTTACATGATCA
ATTGAGAAAAGGAGTAGATAGTATTGGAACATCAGAACACAAATTAAAGCAAACATTAGAGGTTTGGAATAAATTCG
ATCAAACAATAGAATCTATAACTGACTGGCTAAAAGATATGGAATCTAAATGTAGAGATCAAACGTTATGTTCTATG
TTAGAAGAGAAAGAATCTCAGTTACAAAGATATAACGATCTCAGAAACAATATCTATGCTAAGGAAAAAGAAATAGA
CGTTTTTGTTGATGAATCGCATAGTCTTATTCAGCTTAGTGGCGTAGAGAGACTTAAACCTTTAGTTACTCAAGTCA
ATAACCGGTACCAGCAACTGCATTTAATATCGAAAGAAATTGTAAATCATTGGTCAGAACTAGTAACAGATCACAAA
AAGTATATTCAACTTAAAAATGAATTTGAATTGTGGCTTCGTCCATTAGAAGAACAGTTAGCTCAGATCACGACAAA
AGAAGATAAAATGAGTATTGAAAGTAAAGGGAATAAATTACAAAAACTCTTATTAGAAAAAGATAATGGTGAACATA
AAATAGGAGTTCTCACTAGTGCGGGTGATAAAGTTCTGCCAGAAACTGCAACTAATGGAAGAGAAATAATAAGAGCT
GAAATAAGAGATTTACGAGCGAAATGGGATAAATTCAATGATGGCATTATTCAACAGCAAAAAGATCACGAGTCCCA
AGCCTTGCAATGGTCTAGCTATCAAGATGTGCTCCAGCAGACTTTGACGTGGTTAGATGAAAAAGAAAAACTTATAG
AATCAGAAGAAAAAGCTACGCTTAATACAGCCCAAGAAATAAAATCTAAATTATTAAAAAATAAAACACTGTTACAA
GAAATCAATTCTCACAAACGAGTTATTGAAACAGTAACAGAAAAAGCAAAAAATGTAGCTGGCGTGCATTCTGATAA
ATCTGAAAATGATAAAATGGCAGCCACAATTAAATCAATTTGGGATCGATATAATACTTTGACAAATAGACTAAGTG
CTGTAATTAACAGGCATGAACAATCCTATGAAATTTATCAACAGTTTGCTGATCAACAAAAAGCTCTACAAGATTAT
CAAAAAAATCTATGGGATCGTTTACATGGTTTATCAGATTTTACAGGAAATAAGTCTACATTAATGAATAAATTATC
TAAGATACAAGAGTTATTAGATGCTGTACCAAATGGAAATAACAAATTAAAAATTTTGTCAGATTTAATTGAGAACA
ATGAAACTAAGCTTTCACCGCGTGGCAAAAAAGCTATGACGCGGGAGCTAGGCATATTAAAAGCAGATTTGGAAAAG
TTTAATGCTACTGTACATGATGTCAAGAGAGGCATAGAAGATAAAATTCAGCAATGGATTGAGTTTGATAGCGCTAG
TGATCGACTGCAACATTGGTTAACTGACACAGAAATGAGCCTCAAAACATATACACCAAAAGCTACACTAGAAGAAA
AGATCGATCAACTTAATAAGTAC

Brimia:
>cne6566.2.1:T111C | Cell growth-regulating nucleolar protein
GTAACCTCAAAAAACATGGTTGTCTTTACTTGTGGCCATTGCGGAGAGTCTGTCAACAAGCCTAAAGTGGAAAAACA
TTATTTAACTAAATGCAAAAACCGAAACCCTAACCTATCATGCATGGACTGTTTTAAGGACTTTTTG

>cne293.8.1:T183C | Fas-associated death domain protein
ATGACCCTATCGGAGTATACACGACTTAAACAAGATGTTGTTTTAAGCTTTGCTAATAATGAAAATCATACTGCGAT
CTTAAATGTGCTAAAAGAATTTTACAAAGACGACATCGACTCTAAAAGGAGATTTGAAAAAATCTGTACTATTGTTC
AATTACTAAAAACTTTAGAAATTAGAGATATATTGTCTGAAGATGACATAGGACCCTTGAAAGAAATAGCTCGTAAG
CTAAACAGTACCACCCTGTTAAGGAAAATTTGTGACTACGAAAGAAATCATATACCCAGGGAAAACATCAATTATTA
T

>cne4269.3.1:C933T | Zinc finger protein ush
GAAGACGAGGAATGGGGAAACGAGAGCGAGGCGATGCCAGGAGAACCTCGCACACCGAGCTCAGGGGGTGAAAGGGC
GGCGTCTAGCGGAGATGCCTCACCCACTTCGGAAGGCTCCGCCACAGCTTCGCCTCCACGATTACGCCTCAACACAA
GCCTTGCTACAGATCCAGCACTCGCACCACAAGCTGCACCACTTAAACATGAACCCCCTTCGCCATCGCCACCGCCG
CAACCTGCGCAGCAAGCAAGAGACTATATAGCTTTAACTGCAGCAAATTTTCCGGCGCTATTCCCAGCAACTGCAAC
TCCGCCTGGTTACATGTGCCAACCATGCGGGATTCGTTACTCTTCACTGAGCACGCTTCAAGCGCATCAAGAACATT

ATTGCTCCAAAAGGAGGACGAAACCAGATGGCACTGAGACACCTGTCGACAGAGCTGCGGATGAGTCTAGCGGAGAA
TCTAAAGCTCCTCGACCACTTGGCAAGCAATATGCTTGTACTTACTGTTCCTATAGCGCTGATAAGAAAGTTAGTTT
AAACCGCCATATGCGAATGCATTCTTCTTCACCTGTAAGCAGTGGTACCCCCGCTCCAACACCAACTTCTAATGGTG
ATTCTACAGAAGGTCCACCTTCCCAAGATCGGTACTGCGTTGACTGCGATATAAATTTTAGTTCTGTTAAAACTTTT
CGTGCTCATAAAGCAAATTACTGTAACACGCGACAAGTAGTAAAACAAGCCCTCGCTGCTGCAAGAGCTGGTTCCGT
CACATCGGGGTCTGCTCCGCCTTCGCCGGGCGCTACTCCTCCTGCAGCAAATCAATATGCACTTGCTTTACCAACAA
ATCCCATTCTTATAGTACCTTACTCTATTTTACGAAGCGCTAGCACATTGCCGGGCACCACTCTTCCTGATCCAGAT
ACTCCATGTTTTCTGTTACCTAATGGTACGTTTCAGCCTTTAAGTCGCGCGTTACCTAATTTGAATGCAGAAGTTAA
AGAACCAGAAGTCCTAAAATCTGCTAATCGACCTCGGGAACCATCGCGAGATGGAGCAACGCCTTTAGATTTAAGCG
TTCGACGTACACCTGAATCCTTAACAGCTGACGATCATGAGAAAGAAAATAGAGTGCGCTCTACGACACCAGAGCAA
ATTGTATGCGCTCCTTCACTGCCTGGTTCTCCAGGTACACCATCTCCATCAAGACGTTCTTCCTCACCAATCGGTGA
AATATCTCCGAAAAGACGTCGACGTAATTCAAGAGGCCCCACCCCAAAGCCTCCAAGTGTATCATCTCCACCAGATC
AAAAATTTACAGCTTCACCAATCATACCTCCAGCAGTAGCATTACGACTTGCAACAGAACTACCTGTAACTGCTGCC
ACTCCACAAGTACTTGTTAAACAAGGAGTTTCGAAGTGCAAAGAATGTAATATAGTGTTTTGCAGATATGAAAACTA
TCGTGTACATAAGCGATATTATTGTTCAGCGGGTAGTGGAGAAGAGAGAGCTAGTCCTGCACCACCTGAACCGGGTC
CTCCACTGCAATACCGGCAATTGATTTGTCTAGCCTGTGGCATAACTTTCAGCTCTTTAGATAATCTGACAACTCAT
CAATCATACTATTGCACGAAGAGAGAGACCAGATCGCCACGAAGTGTGACGGATGCTCCACGACCGTCCTCGGGACC
GGAAGGGGGTTGGAAATGCCCTTGCTGTGAAGTCATATCTCCAACTGCAGCAGCCGCACAAAGACATATGGAAGCAC
ATGCTGGGGTCAAAGCTTTCCGGTGCACTATCTGTCGGTACAGAGGAAACACATTACGAGGCATGCGCACTCATATT
CGCATGCATTTCCGTGAGAAACCAGCTGATTTACAG

>cne22329.2.1:A1358T | NF-X1-type zinc finger protein NFXL1
ATGGCGAGGAGGTATCGCGACGCTGCCGCGAAACTACAGAGTAATGTTCAAAAACATTTGCAGGTGATGAAAGATTT
GTCGTCCTCTGAAGACGAAGAACCGTTTGATTCGAATGTCCTGGAAGGTGTATTTCAGAATTACTGTAAAGGTGGAG
GCGACAAACAATTGCTGAACAGAACAAAGAATCTACTAGAAGAAGCAATAAGTGGCCGTTCAGTAACTTGTCTCATA
TGTATAAGCTCTATCAAACGTGTTGATGCGATTTGGACTTGTAGTCACTGTTACTCATATTTCCATCTATCTTGTAT
ACAAAAATGGGGTAACGATAGTGTAAGTTTGCGCAGTGAGGACAACTCTGGCCCTGTTACTGTATTTAGGCCCCAGA
AAATAGAATGGTGTTGCCCTAAATGTAGAAATTCGTATAGCAAAGATGAAATTCCTCGTAAATACCGCTGCTTTTGT
ACAAAAACTGAAGATCCTCCATTTCATCCATGGCTACTTCCACATACATGTGGAGAAGTATGTGAAAAAAGACTCTC
AGACGGTAATAATTGCAAACATAAGTGTCTCTTGTTGTGTCATCCTGGACCATGCCCACCATGTCCTCAAACAGTTA
ACAAAGTGTGTTTTTGTGAAAAGGAACATAAAAAAGTCAGATGTAGTTCATCAAGATGGTCTTGTGGAGCTCAATGT
AAGCGATTGTTACTTTGCAAGTCACATAAATGTGAAAGTATATGCCATGATGGTGATTGTCCTCCTTGTAAATATAC
CAGCTTACAATCATGTCAATGCGGAGCTGAAAAATCCAAACGTCCATGTAATGACTTAATGTGGCAGTGTAACAAAA
AATGTAATAGGCAATTTTCATGTGGCTACCATATATGTGAAAAAATTTGTCATTTTGATGAATGTGGAATTTGCCCA
TTTTCTGGAGTGAGATCTTGTCCTTGTGGAGCTAATCAACGATATATTGAATGTCCAGATACAATTGAAACATGTCT
TGGTACTTGCAATAAAAAGCAGGATGATTGTGAACATAGCTGTCCAGAAAAATGTCATAAAGGTCCTTGCCCTCCCT
GTCAAGTTTTAGTTGAGAAGAAATGCGTATGTCAAACACACTTTAGATCTCTGCCTTGTAGTAGGGAATTCAAATGT
GAAACAAAATGCAGAGGCATGAGACCTTGTGGTAAACATGTTTGTGGCCGTAAGTGCTGCAATGGAAGCTGCCCACC
TTGCATAAAAAATTTGTGACAAACCATTGCAATGTGGTCGTCATAAATGTACTTCTATTTGTCATCATGGGCCGTGCT
ATCCTTGTCCAAGAGAATCTAAGATTACATGCCGTTGTAAAGAAACTTACATTACAGTTCCCTGTGGCAGAGAAAAG
CATGTAAAACCCCCAAGATGCAATTTACCATGTAAAATTAAATACAAATGTGGTCACGTTGAAGATAACAAGCATGT
ATGCCATTTTGGCGACTGCCCTTCTTGCAAGGCAATTTGTAACAAGAATTATGAAAAATGTGACCATGTATGTAAAG
CTATATGTCATGAATTTGTTTTAGTTGAGTTTAAACAAATAGAAAAACCTGCTACTCCCTGGGAAGTGCAACCTGTA
AAAACGAAGATTGTAAAATTAGATTGTCCTCCATGTGAAACACCAGTGGAAGTAATATGTTTTGGGGAGCATGAAAC
AGAGACTCAACCTTGTCATACAGCTTCTAGAAGGCCCTGTGGACGCGAGTGTGGCAAACCACTTTCTTGTGGAAATC
ATTCTTGCAGTCTACTTTGTCACTTATATAAACATGATCCAGCTTATCCATCAGTGCCTTATAATTGCAAACCGTGT
GATAGAGCCTGTTCTGTTATCCGTCCAGAAAAGTGCACTCATAAATGTGCATTACAGGCTTGCCACCCAGGTGCATG
TCCTCCATGTAATTTATTAGAACGTATACCATGCCATTGTGGTGTCGCAGATATATATTTACGTTGTTCGGAAATTG
CAACAGCTACAGAAGAGATTCTAAGTTGTAAGCAACAATGTCCAAAGACCTTAGAATGTGGGCACCGCTGTAAGAAT
CAGTGTCATTCTGGAATATGTGGATCCAATCAAACTTGTACTAAGAAGACAAAGATCTATTGTCCTTGTGGTAATTT
AAAGAAAGAGGCACCTTGTAATGTTGTAAGGAAACAAGAAGTGAAGCTTACTTGTAATGAAAGTTGTGAAGCTAAAA
AACTTGCAGCAAAAATAGAAAAAGAGAAAGAAGAGCAGAGACTTAAAGAATTGGAGGACGAAAAAAACCGAAGAGAA
CTTGCTGAATATGAATGGAAATTAAGTGAGAAGAAGAAGAAATATAAAGAAAAGAAGGTTGTGGTTAATGTAGACAC
TCGAAATTGGCTTCAAAGATATTGGGTTCCTATTTTAGCTGTACTTGTAGGATTAATTTCCAGTGTTTATATCCTTA
GCATT

>cne37625.2.1:G450A | Ankyrin repeat domain-containing protein 27

ATGGCTGGTTCTTATGACGAAATAATCTCCGAAAATCCATTCTTCATTGAACTTAAAAAAGAATACACAAGTTTGTT
CCATCATTGTATATCTGAGTCGTGGATAATTTGTGTACCGAGGATAGGCAGCTTGACCACTCGTGTGTTTACCGTCG
AGGATTTCTGTGCTCATATCCTCGTACCAAGCGATGAGTTACCTGAAACTCACTACAGCACACTCACCGAAAAACAG
GCAACCGTAGCCAACAAAGTAATAACTCTTGAGGTTACAAAAGGATTACCACTTCAGAGTCACATACTTTTTGAAGA
AACTTTCTATACAGAAGACTTTATAAAATATATGGTGTGGTGTATTGAAACACCATTAGAACCGTCAACTAGTGTAA
GTGAAAATGTGTCAAAAGATTGTCTTACATGTATCAATGATTGTATTGACTTACTATGGACACAAGCAGCTGGACGT
CAAGTGCTAGACCAAATTGAACTGAATGTGCAACTATTTTTAAAGAAACATACTACATTACCAATAGCTATAGCACC
TTTAAAAGATCTAATTAGTGATTTATACACTCAAAGTTTGCAGTCTGCCCTCCAAAACCGTAGACTACGAGAGAAAA
CAAAAAATTCTAAGCATTTATTGGAAAATATCAAATTAGCTGTAGAGAGTTATATGCAGCATTTACTGTTTGATACA
ATTTTTAAAGCAATATGTACCAGCTGCGCATACGAAGACTCTCATCTTAATAAGAAAATAAGAAATATGTGCGATAT
CCAGTTACGAGACCTTGATATAAAAAAAGAACTCTATCACGCAGTGCCAAAAGCAAAGCAGATTTTATCAAAAATCA
ACACATATAATACTGTATTAGAAAAAGTTATTTGTCTTAAACAAGCCCTTAATACTGTCAACAAGATCGATAGTAGC
AATAATGTTGTTCTTTTAACTGCAGATGACCTACTTCCAGTATTTGTTTTTCTTCTAATAAAGTCAGGGTTGCCTAA
TTGGTACAGTCATTTGACTTATATGAAAGAATTTAGACTTAGCGGCCTAGGAAAAAGTGAAGGAGATGAGAGTGACT
TTCTTATCACAACTTTGGAAGCTGTCATTGAACATATCCAATCTGGTGCATTAACAGGCCCACCTAACCCAGAAGCC
TACTACTATGACAGTAACTTCACAATTGATGACAATATAAATAACAACAGAAAAAGCAGTTTGACAGAATCTGTTTC
TACATCATCTGGGAGAGAAGAAACCCTGGAATATGTTTTTGATTTAATAAAAGCCAATCACATTGAGCAAGTTGGTA
TTTTACTAGAAAAAAATCAAAAACACATAGACAGTATTCAAAAAAATTATAACTTTGCAGTAAACATTGCTGCTGAT
GAACAGGATGATGATGACGAAGATGAGGAAGATGAATATGAAAATATGACTGAAATATATCAAAAGTTATGTCACCC
TCTTTGTAATTGTAGAAAGTGTAGCTCTAAAATATCTAAAAACTTAGTGAAGATATCTCCTACTGTCAGTTCAAGAG
ACAGTCATGGATTAACACCATTACATGTGGCATGTATACATGGAAAAGCTTCAATAGTAGAAATACTAATTGATATG
GGTGCTGAGGTTGACACAACTGATCTCAATGAATGTACACCCTTGCATTATGCAGCTTCTAGAGGACATCAAAATGC
TCTGTTGCTGTTACTACATAGTGGTGCTAAAGTAAATAAAATAAATATTGATAAAAACACACCACTTCATCTGGCTG
TTAATAATGGCCATTTAAATTGTGTAAAAGCCCTCATATATTTTGCAGAACATGGTCGAAAAAATATAATGATAAAT
TGTAAAAATGAAAATGGTAACACTCCTTTGCACTTAGCCTCAAAGTGGGGTTATGAAGGTATTGCCAAATTATTAAT
AGAAAATGGTGCTGAACCATCTCTACAGAATAATAATAATAAAACTTCATTTGATTATGCACACAATTTGAAAATAC
TAAATGTTCTCAAATCGAGCACTCCAAATTTATTTGAATACATACACATCACAAGTTCCCAAGTTAAAACATTAAAT
TGTAAGTCAGATGATTTTGTGTCATTGAAGTTACAGAATTTAAAAATCAAGCCAAATGAGACGAGTAATACTTCAAA
AACTATAGAAAATCTGAAAAGAATTGATAGGATCTTGCAAGCCATAACATTCGGAGATGTGAAGCTTGCTTGTTTTT
ATATGAACATTGATTATGATAACTATGTTAATACAAAGAGTGAAATAAATCGATCATTGTGTCATCCTCTTTGTGAG
TGTTCGGTTTGTAAAAATACCAATTTACGAGTATCTGATTATGATGTTGATTTTGCAGACACTAATGGTTTTACCGC
ACTACACTATGCTGCCCAATATGGATTAGATGAGTTGTGCAATATTCTATTACTAAATAAATCTAATGTAAATTGTT
TTAATAAAAAAGGGGAAACACCTCTTCATATGGCATCTACAAATAACAGAATATCAGTAATTAATGTTCTCCTTAAT
AATGGAGCTAATATTAATGCCATAGATATTTCTGGCAACACTCCTCTACATAATGCTTGTGAGATGGGAAATATAGG
GGCAGCTAAAACATTGCTATGTTATAAACCAGATATCACCTTGTTGAATGGCTCTAATAAAACTGCATTAGATGTAG
CTAAAGCTAAGGTTCACTTAACAATAATAGATTTAATAGATAAACATACTAAGCAA

***Tenedia*:**
>cne11306.1.2:T60A | ATP-dependent RNA helicase p62
TTGAATGTTTTGTTACAAGAGATTGGTGAAAGTCAAGAACCTGGAGCAAAGACTATAATTTTTGTTGAAACTAAAAG
AAAGGTTGAAAATATCACAAGAAACATAAGGCGTTATGGGTGGCCTGCTGTTTGTATGCATGGTGATAAATCACAGC
AGGAGAGAGATGAAGTTTTGTATCAATTTAAACAGGGCCGTGCTAGCATTCTTGTAGCTACAGATGTAGCTGCTCGA
GGA

>cne5833.2.1:A108G | Cyclin-dependent kinase 9
ATGCAAGGCGTAAGTGCTCCTCGTGAGCCAGCACCAGTCGCCAGTACAAGTTCTACCATCCTAAATATGAGAGAAAA
GGAGAAATACATAGAAGATTTCGAATTTAGATTCTGTGACGAGTCTTCGAAATATGAAAAAGTAGCTAAAATAGGTC
AAGGGACGTTCGGGGAAGTCTTTAAAGCCAGAGCAAGAAATAGTAATAAAAAGTTTGTAGCAATGAAAAAAGTCCTT
ATGGACAATGAAAAAGAAGGATTTCCCATCACAGCTTTACGAGAAATAAAAAATATTACAACTCTTAAAACACGAAAA
CGTGGTAAATTTGATCGAAATCTGTCGCACAAAGGCAACAGCTATTAACAAATATAGGTCAACATTTTATTTAGTTT
TTGACTTCTGTGAGCATGATTTAGCTGGACTGTTATCGAATGTTAATGTGAAATTTAGCTTAGGTGAAATTAAAAAA
GTTATGCAACAGCTTTTAAATGGACTTTATTATATACATAGCAATAAAATTTTACATAGGGACATGAAAGCTGCCAA
TGTATTGATTACTAAAAATGGTATTCTAAAGCTAGCAGATTTTGGTTTGGCGAGAGCTTTTAGTGTGGCAAAGTCAG
GACAAGCCAACAAATATACTAATAGAGTAGTGACACTTTGGTATAGGCCTCCTGAATTGTTATTA

>cne12476.6.1:A395G | Type II inositol 1,4,5-trisphosphate 5-phosphatase
ATGAATCACGAAGAAATTAAATCTGTGGTACAGGAGAAGTTCTTACCAAGTGAAAAAGTAACAGCATTTCTATGTGA
TGCTAAAGTGTTTCATCCAGAATGTGTAAAAACAGATAGACTTTTAGCAATCGTCGAGTACGGAGACGAAAAAGCTG
TTTTTTGTTTATTTACGTCATGTTACCCACCGAAGTCTTTCACCGATTTATCTATAGAAATAGTTTTACCTATCGAC
AATAGTTTAAAATGTGATATCGACAACAAACCATCAGATTTAGAAACTATACTCTTTTTAAATCTTCAGTCTCGTTC
TAATACGATTAAGATTGAAATCCAAATGACTCCACGAGTGGCCAACTTCGTAGACGATTTGTTTCGTGGAATAGAAT
ATGTAAACAACTTACAAACGCAACCTGACTTTGTTTGGCTTAAGAAGTACAACGGTAAGAATGAAGATGACATCCTC
GCCCACACGATGGTTGTCCGCCGTGCTATATCACATAATCAAAGCCCTGCACCAGTGGCTATCCGAGAAAGCCTTAT
TAAACGCAAGATGTGTGATAAGGAGTTTGAATACACATATACACAAAATTTTACAGTTTACTGTGGTACATGGAATG
TTAATGACAAATCTCCTGTCATACCTCTGAAGAACTGGCTAAGTGTTGATAAAGAACCACCAGATATATATGCAATT
GGCTTTCAAGAATTAGACTTGTCAAAAGAAATCTACCTCTTTGATCAAACTGTCAGAGAAGATGAGTGGTACAATGC
TGTATTAACATACGTTGATAGACGAGCAAAATATATTAAAGTAGAAAAGGTAAGACTAGTTGGCATGATGCTTATTG
TGTTGATAAAACAGGAACATGTCCCTTATGTACGCAATATAGTTAGCGATACAGTAGGTACAGGGATTATGGGGAAG
TTGGGAAATAAAGGTGGGGTAGCCATAAGATTTGATCTTCACAACACATCTATGTGTTTTGTTAACTCACACTTAGC
TGCCCATGTAGAGGAATATGAAAGGAGAAATCAAGACTTCAGAGACATTTGTAATAGGACGAGATTTGCTCAGCCTA
ACCAACAGTCAAAAGCAATAAAAGATCATGATCAAATCTACTGGATAGGAGACCTTAATTATAGGATAACAGAACTG
GATCCTGCAACTGTCAAGAAATTAGTAAGTGAAAACAATTTTGTCCCAGTATTGGAGTGGGATCAATTGAAACAACA
ACATAAAAATAACAGTGTTTTTGTTGGATATACCGAAGGCAATATAACATTCAAACCAACATACAAGTATGATACTG
GGACAGACAACTGGGATTCCAGTGAAAAGAACAGAGCACCAGCTTGGTGTGACAGAATATTCTGGCGAGGGGAAAAT
ATTACACAGCTAGAGTATCGAAGCCATCCTACTTTAAACATAAGTGATCATAAACCCGTATCAGCCATATTTAAGTG
CAAAATAAAAATAGTAGATGAAGAAAAGTACAGAAAAATATATGAGGAAGTTATTAAACAACTGGATAAACTGGAGA
ATGAGCTAATACCGCAAGTCAAAGTGGACATAACTGAAATTGACTTTGGAACTGTTAAATATTTAGAATTAGAAACA
AGGACCATAACAATAAAAAATACCGGGAAACTGCCAGTGGAATTTGAATTCATTAAAAAACTAGATGAAGCAAGTTT
TTGCAAGGAATGGCTCATAGTCGAACCTTACAAAAAATGTATATGTGCAGATGAAACATGTGAAATACAATTAAAAG
TACTTATAAATAAAGTTTCAGCCTGTAAAATGAATGCTGGGATAGACAAACTTTATGATATATTAGTTTTGCATTTG
TACGGCGGCAAGGACATTTTTATAACAGTTAACGGTACATACCAAAGAAGTTGCTTTGGGTGTTCCATAGAAGTTTT
AGTTAATTTAAATATGCCTATCAGAGAAGTACCTGTTGGTAAACTGATAGAATTGGAAAGTAAGAGGGATCAGTGTG
TTTCAAATCAATCAACATACTCTATACCAAAGGAAATTTGGTTTTTAGTAGACCACATTTACCTACATGGACTCAAG
GAACCAAATTTATTTGAACAACCTGGATTACACTCGGAAGTTTTGCAGATAAGGGACTGGTTGGATAGTGGATCTAT
AGATCCCATTCCAGGTAGTATCCATTCTGTTGCTGAGGCACTGTTGCTGTTATTGGAGAGTACAGCTGATCCAATCA
TTCCATATAACCTTCAGTCTATTTGCTTAAGGTCTTCAACTAACTATTTGCAATGTAAACAGCTTGTAATGGATTTA
CCTGAATTTAGAAAGAATGTATTCTTATATCTTTGTGAATTCTTGCAAGAAGCCTTACAACACAGTGCAGAAAATGG
TTTAGATTCCAAGACATTGTCTACGTTATTTGGTTCAATATTTCTTAGAGATAATCCTAATGTAAATCAGGAGCCAC
AGTCAAGAATAAACAAACAAGTAACTGATAAAAAGAAAGCCCAATTTGTATATCACTTTTTAATGAATGATCACAGT
GACTTAATATTTTCACGA

>cne18163.3.1:A186G | 60S ribosomal protein L9
ATGAAGCAAATTGTTGCTAATCAGAAAGTGAAAATACCTGAGGGCCTAACCGTGCACGTTAAATCACGGTTAGTGAC
AGTAAAAGGTCCTAGAGGAATATTGAAGAGAAACTTTAAACACTTGGCTGTTGACATTCGCATGGTAAACCCTCGGC
TTTTAAAAGTTGAAAAATGGTTTGGTTCTAAAAAGGAACTAGCTGCTGTAAGAACCGTTTGTTCTCATGTAGAAAAC
ATGATTAAA

>cne2337.5.1:A603T | Glucosidase 2 subunit beta
ATGAATTACTTTTCGTGGATTAGAAGAATCCAATCGTGCGTGTTAATAATATTTTTAGTTGTTATATCCGCTCAATC
CGAGGTTCCTAGGCCGCGCGGTGTGTCTTTATCTAAAGCGTCGCTATATTCGCCGACTAAAGATTTTACTTGTTTTG
ATGGAACAAGTACTATACCATTTAGTTATGTAAATGACGATTATTGTGATTGCTTCGATGGCAGTGATGAACCCGGC
ACATCAGCTTGCCTTAACGGGATTTTTCATTGCACAAATGCAGGTCACCAACCTTTGAACATACCAAGTTCTCGTGT
CAACGATGGTGTTTGTGACTGTTGTGATGGCACGGACGAATATGCGAAATCGGAACCCTGTCCTAACACATGTGAGG
AATTGGGAAGAGAAGCCAGAGCAAAGGCTCAACAACTAGCAGATCTTCATAAAGCAGGCAATGCATTAAGGTTAGAA
CTTATAGAAAAAGGTAATAAAAAACGAAGTGAAATGGCGGAGCAGCTGAATCAGCTTCAGAAAGATAAATCTGAAGC
CGAAAGAATCAAAGACGAAAAAGCACAGATAAAGGATCAATTAGAGGCAAAAGAGAATGAAGCACTTCAAGTGTACA
GGGACGCTGAGGAAAAGGAGAAACAAAAGAAAGCAGAATTAGAAAGACAAGAGCAGGAAAAAGAGGCATCTGAGCAA
TTTTCAAGGTTTGATTCAAATGGTGATGGTGTTTTAACAAAGGATGAAATTAAAGTTGTAAATACCTTTGATAAAAA
TAAAGATGGAGAAGTTGATTCTGAAGAATTACAATATTTCTTGGGTGAACATGAAAGTGTTGATAAGGACTCATTCT
TGTCGACAACATGGCCATTAGTGAAACCATTGTTGATGATGGAGCAAGGAATGTTCCGCCCTGCTGATGCAGAGGAA
GAAACTGAACATGCTGATGAAGAACAACCTGCTGATGAGGAAACTGAGGATGCGGATGGTGAAGAGGATCCAAGTGA
TTTGGATCTTCATGCAGATACTGATGATCATGAAATAGACACAGATACATCTAAAAGCTATGATGGTGAAACCCAAA

GACTTATTGATGAAGCAACAGAAGCTCGCCGACAATACACAGAGGCGGAAAGGACAGTTAGAGAAATTGAATCAAAC
ATCAAAAATTTCCAGCAAAACTTAGAAAAAGATTATGGATTAAACCAAGAATATGCAACTCTTGATGGTGAATGCCT
AGAATATGAGGATAAGGAGTATGTTTATAAATTGTGCCTGTTCCAGAAGGTAACACAAAAGTCAAAGAATGGTGGTA
TGGAAGTAGGTCTGGGTAATTGGGGTGAATGGGCAGGAGAGGGTGATGATAAATATTCAGTCATGAAGTATACAAAT
GGAGTAGCCTGCTGGAACGGGCCTAGTAGAATGACAACAGTAGATATTAAGTGTGGGCTGGAAAACAAATTGATGTC
TGTCACTGAACCTTACCGTTGCGAATATAAAATAGATTTTGTCACTCCAGCCGCATGTGATGAATCTCGCCACACAA
GACAACAAACATCACATGATGAACTA

Xanthosa:
>cne658.3.1:T1835C
ATGAAAACTAAACAAGCTAAAGAAGAGTCAGAAAAGAGTATTGATGAAGAATCAGTAGTTGAACCTAAGACCCGTGC
CCGTAAAAAGGCTACAGATCCAACGGAGGAAGACAAAACCAAGCCCACACAAGATGACCCCAAAACCTCTAAAGAAC
CTAAGAGAAATGTAGGGAAACCTAAAGCAATAACTAAGAAAAAACCTCTTGGTATTAGAAAGACAAAAACTCTTAGA
GGGAAAAAGGTACCAATCACTGCTACAAAAAAAAATGTTTTGAGACAGAAAATTACCAGGACAAGAAAAGCTGCTGT
AAAAACTGAACTTGACCAAGAAAAAGAAACCGTCCCTTTAATACCGGCCTCTGAGATTAAAAAGGAACCACCCGAAG
AAACCACATCTAATTCAAGATCTTCCAGCCCTAAGACAACTGGACGCAGGTCAAGACTTAGCTCTGATATGGTAATG
ATGAAGTCAGTGCTAGGAGATTCTCCAAACAATTTAGTTCACGGGCCACGCACAAGTCCATATTCCATGCGCTCGGA
GAGGAGTAATAGCCCTTCTCTCTTTGATGGCAAGAACTTGAGAAGTGGTAAGCCCAGGAAATTAAAGACTAATCTTC
TAAATGAAGTTGTAATGAAAGAACAAAAGAAAAGGAGAAGATTGTTGTCTGATGCGAAGCCTGATTCCACAGAAAAT
TCAGAAGACTGCAAGAAATCGAAGAGAGGCCGCTCTTGTTCAAGGGATGGCTCTGAGATCTCAAAATGCTCAGATGT
AACTGAATCGGATATTAGCCTTAACGAGGCACCTAATGATGGTGAAACAAAGGAGCTCAACAAAAACTTAGATAAGC
CTAAGACTGACCTAGATTTAGATAATGAAACTAATGTAGAGATCTCTAATATGGGAAAAGTGCCTTCAAATGTTGAC
TCCAAAGTCACAGAAAATAAATTGGACATAGAGCTTAGTTCTGTACCAAAAGAAAAAGTTAAGAAAAGGATGAGCTT
AAAACGGAGTACTTCACTAGATTCAGACAACAATAATAGTAATAGAGTTAAGTTAGAACATTTAAATACTTCAGATC
TAGATGATAAATCATTGTTTAAGACTGACGGTAGGCTTTTCGAAGAACGAAGTAGTATATTGACTTGTATGTCAAAA
ACATTTAACTCGAAGGAGGTTTCAAAAAATATAAGGAAAGCAAGAAGAGGAAGGAAAGCAGCAGCTGCCTCAAGACT
AGGCCCAAATCAACCCTCTAAGAATGGAATAAGTAGCACAATAACTAATGACTTAACAGAGGACAAGCCAGAAACAG
TCGACTCACTTTCAAAAGAAATTAGTGACCTTATTAATGATTTAGATCAGAATATAGATCGTGATCAAGAACTGACT
AATGATACTTTCTTAGTATCAGATCAAGCTCAAGTTAAGCCTTTAGGAGATAATAATAGCATTGTTACACCAGACAC
ACCAATAAAGGATAAAGAAAAGAGTTTGACAATACCAAATGAGTGTACGCCATCTAAAAACGATGATAGTGAGAATA
TAAGGCTACATTATGAAGAAGATTCGGCAGAAAAATGTTTTGAAAACAGATCAGAAATAACTTACAAGGGTAGCTCT
GTTGCTAGTATAGATAAATTAATGGATAGATTAAAAGAATTTGAAGAGTTTGATAAGAGAAGACAAAGACAGGAATC
TGAATCTAAATCAAACGAAAACAAATCAGTAATGGTTACTAATGACGTGGTACTAATACCCAAGTCAGGAGCTGACA
TTAAGCCTCTAAAAGACATACAAACTGTACGATCTCCCGAAAGAGTTATTGAAAAAGAAAATGTTTTATCCTGTTTT
TTCGAAAACAATCCTGCTGTTTCTATAGTAAAAAGAGATCAAATGAGAAGATCAATTGAAATTGACTTGCCTAATTC
TGTTACTCTTATAAAAAGAACAGTTAACTCACGTAAAGAATCAACTAGCTCAAATCATTCGAGAGAATCAGATGCTA
TTAGTATATTTGAGAAATCACTGGGCAAAGATGTGACATTGACAGAAATTAGAAAATCTGTGGAAAAACCTACTCCT
GCTCCACAGATAGACCTGCATCAATTTGCAACTTTGCATCCAAACAGCCCAAATCAAACTGTTGCGGGTGTTGTATT
AGATTCAAGACAAATATCGATAACGCCACGCAACATGGATGCCAAATCAAGTTGTAATGAGGTGCAAATAATGAAAA
GGAAATCAAGTTTGACTTTATCTCGAAAATCTTCCGAGTCAGGCTTAGAAATAGAAACTGAAAGACTAGCTACACTA
GAAAAGATTAAATCGCCGCCTCATCAAATGAAGTCTCCCGCGATATCTATTACAGCAACGCCAGTAGCGATCAAGTC
TCCAGAAACACAGCCTGTGGAAGCAGAAGCACTGCCACCGCCAATAGATACTGTCAAACCTATTCCAGAACACATAA
CTTTGGCCTCTGAGGTCCCAAAACTAGATAGCATATCAATCGATTTAGGATTACCAGAAACTAGTATTGATGCTCAT
CATGCTCAAATTATTCCAGTCGCAGATAGTAAAAAAATTGAAGAACCAAAGGATAAATTAGAAAAGGATGCTATTCA
GAAAGATGAGCTAATGGAAACAGAATCAATTACTAAGACTGATGCTGTTGCTCATGCAGTACCTTCAGCCACAGTTA
CGGAATCAGTGTTAAATTCCGTACCTGAATCAGTGATGACTGAAGAACCGATTGTTGAAGACCCAGTTTTACCAAAG
GAATCTTTGGCGAAGACAGAACCCCAAGCTAAGGCTGAACTTGTCTCTAGCGCAGAACCTGTTTATAACACAGAATC
TGCTAAAGAAGATCCTAATGTCAAGATACAGGAAGTAGACAAAACAGTTTCTAAATCAGAGTTAGTCGCTAATACTG
AATCTGTTACTGAAAATGTAGCTGTGTCTAAAGTTGAAGAAATAGCTCCAAAGTGTGAGATAAGTAAGCCATTAGAA
CAGAAGGTGTCCGATATTAAATTAGATGAAACTAAAGAGAAAAGTTCGAAAGTGAAATCTGCGAGAAATTCAATTGA
AAACCAACCTGGACCGAGTAATATTTTACAAGAAACACCAGAAAGTCAAAAGAGGAAAGAAAATGTTTTACGCACTC
TTGGTCTATTAACGCATAAGGCCGCTAATGAAGCAAAGATCGAGAAGCAGAGGGAAAAAGAACGTATTTATGGGTCT
AACTATTCAGGTATTATGGGCAAAGGAAAATCTGGAAAGTCTGACTATACTGGCACTTTAAAGACGGTTATAAAGTT
GAATCGAGGTTCTGGTGAAAGAGATAAAAAGAAGTACAGAAGTTCTCTCAAGATGACATTCCAGAAGAGTAAGAACA
GGTCCGGAAGACCACAACTGCAGGTCGGGGAGCCGGGAGAGGGTGACGCGTACTACACAATCGAAAGACGAGAG

>cne29742.2.1:T1372C | DNA polymerase epsilon subunit 2
ATGGGTGACATTCAACAATTAAGATCAGAAGTGATTAATGCGTTTAAACTTGGTGGTTTCACGTTGAGACGTGAAGC
CAGTACCTTTCTAGTAGAACATGTACAAGAATTAACATACCAGGAGAGAAAGAAACTTATAGATAAGTTGATAACCC
ATATTTTACACCAGTGTTTATCCCAACCAGTACTAGAAAAGTCTCAACTTGAACTTGCAATTAAAGAGTGTTCTTCT
TTAGGATTAGAAGAATCAGAATCTATTTTAAATGTTATTGATGCATTTAGTGTACCTAAGTTTTGTTATGATAATGA
AAGGAAGAAATTTGTAAAAAATATTTACGACTCAAAAAGGCTTTATCCTGAACCAAAATGGAAATCACATATGTTAA
TAGACAGGTACACAATTATTTGGCAAAGAACTGCTAGAAATAAATTATTTGCGGCCAACACATTACCATCAATGGAG
AATGAGAATCAGTTTCAGCTGCGGAAAATTGAGGTTCTTTTGAGCAGCTCAAGTAAAGTCAATGATGTGATAGTATT
GGGATTGTTAACACAACTAACTGAAGGTAAATTTTATTTAGAAGACCCAACAGGTAGTGTGCAATTAGACATAAGTG
AGACAAGATACCATTCTGGGCTATTTACTGAAAGTAGTTTTGTATTAGCTGAAGGCTATTACGAAGATAAAGTCTTA
CATGTGATGGGCTTGGTTCTGCCTCCCACAGAAAGCAGAGCCATGTCTATGCCATATTTTGGCAATCTAAATACTTT
TGGTGGACCATCAAAGACTTTGTTGAAAAATTCTCAAAATTTACTCAAATTAGAACAAGAAAATGAAGATGGTATTA
TTGTTTTTCTGTCTGACGTTTGGTTTGACAATTTAAAAGTTGTGTCCAAGTTAAAAACCTTATTCTCCGGGTTTAAT
GATTATCCGCCAATTGCCATAGTATTTTTGGGTGAATTCCTCTCATGTCCATATGGGTATGAACATAGCACACAATT
GAAGGCAGCTTTAACAAACTTTAGTGACATTATTTTGCCATTCACAAAACTACGTGAAAGCTGCAAGTTTATATTTG
TTCCTGGGAGTGGAGATCCAGCTGCTGCTAACATTTTACCTAGACCAGCATTACCAAACTTTGTTACTGCAAATGTA
AAGGACAAGTTAGGTGATAGTGTTGTTTTTACAACAAATCCATGTCGTATTCAATATTGTGCACAAGAAATTGTTGT
TATCAGACAGGATTTAGTGACAAAAATGTGCAGAAACTCAATACATTTCCCTGAAACTGGAGATATACCAGATCACT
TAACAAAGACTCTTTTAAGCCAATGTACATTATCTCCATTGTCTATAGGTGTACAACCAGTATATTGGAAATACTCA
GATGCATTAAACTTATATCCTATTCCTGATTTGGTGGTAGTTGGTGATCATTTCCAGCCATATACTAGGTCATATCA
GAACTGCCAAGTTACTAATCCTGGGTCATTCCCTCGAACTGAATTCTCATTTAAAGTCTACATACCAGCCTCTAGAA
CAGTGGAAGACTCTCAAATACCCAATGATGATACT

>cne8287.9.1:A925T | Mesoderm induction early response protein 1
ATGTCAGATTGTGCACTGGTAGCCAATGTAAACGAACACGATGCCAGTATGGATGTGGGGAACGATAAATCTCTATT
CGAACCTACTATTGATATGATGGTAAATGATTTCGATGACGAGAGAACATTAGACGAAGAAGAAGCTTTGGCGGCAG
GTGAGCAACAAGATCCGAAAGCGGAACTCAACAGCCTTCAACGTGAAGGTGATATGCCATTAGAGGAATTGCTTGCA
TTGTACGGCTATAACAAAACTATGGAGAAAACCATTTCAGAACCACCCCAAGAGGTTGTGCCGGAGGAAAATGAAAA
AAGTGAATCGGCACTTCAGCAATTGTATACTGAAAATGCTAGTCCAGAAGCTACACGCTGTCTTAGGTCTGGTTCGA
GACCACCTTCTGAAGAAGAGGATGATGGCGATTATAGCCCTGAAGAAGATGATTGGAAAAAGACAATAATGGTAGGA
AGTGATTACCAAGCTGGTATTCCTGAAGGTTTGTGCAGTTATGATGATGCTTTACCATATGAAAATGAAGACAAATT
GCTTTGGACACCGACAGTGTTAGATGAGAAAGTTACTGAGGATTATATGAGAAAAATTTGCGCAATGAACTCGGGTT
CTGGTATAGAAGCAGTACCAAGAGGTAAGCAACTTCGAGATGATGAAGAAGCTTTATTTCTATTGCAGCAATGTGGG
CACAATGTTGAAGAAGCTTTACGACGAAGACGTATCTCTGCCCAAACTCCTGCGCATGCTAGTGTGTGGTCTGAAGA
AGAGTGCAGGAATTTTGAAAGTGGAATCAAAATGCATGGAAAGGATTTCCACCTTATACGTCAGCAGAAAGTAAGAA
CGAGATCAGTAGGTGAACTGGTGCAGTTCTATTATATTTGGAAGAAAACTGAGAGACATGATATTTTTGCAAATAAA
ACAAGACTAGAAAAAAAGAAGTATACATTACACCCTGGTCACACAGATTATATGGACAGGTTTTTGGAGGAACAGGA
AGGTACAGGATCAAGTGTGGTAAGGCCAGTGTCTCCATCTCCTATGATGGTGTATGTACCATCACCAGCTGCACAAC
CTGATCCATTAGCATTAGGAGAGAAAGAAGTATTTTCTCAATTGAATGCTCAAACTACTCCTCCACAGAACCTATCA
AATGAGGAGCCAGAGACCGATATTGTTTCC

>cne4342.2.1:C323G | Regulator of nonsense transcripts 2
ATGACCTCTAACGATATGAGTAAAAGTGACGCTGACGTATCCGAAGATACGGAGGAAAATAATAAACTCGCTTTGTC
GGAGTATGTAACAAACTTAGAATTGAGAATTAAACAAAAGTCTGAATTGCGAAATCAAAACTTAAATTGTGTTAGAC
CTCCTGATAACTACTTCTCTAAACTGGATTCTGGTCTGAAGAAAAACACAACTTTTGTAAAAAAATTGAAGTCGTTT
AGTGCATCACAGTTAGATGCACTTTTAAAAGATTTGAGCACGTTAAATCTTACAAAATATATTTCAGAGGTAGCCTC
CGCAATTGTTGAATCAAAGTTAAAAATGTCCGACATAACAGCTGCTCTAAATTTGTGTTCAGTATTGCATCAAACAT
ACGTTGAGTTCTCAGTATTTTTCTTCGAAAATTGGCAAAAAATTCTAACCTTTAAGGCAACAGATAAAATAAGTAAT
TCATCCAAGTTAAGAGTTGATCTAAGATTCTATGCAGAGTTAGTTTCTGTTGGAATATTTACAAACAAAACTGGTTT
GCCTCTGCTCGGAAATGTTCTTACAGTACTGATAAATATGGATAAAGAAGAACACAATAACATACCCATTTTGCTCT
CATTTTGTAAACACTGTGGTGAAGATTATGCTGGTCTTGTGCCAAAGAAAATCCGAGATATTGCTGAG

>cne29742.2.1:C767G | DNA polymerase epsilon subunit 2
ATGGGTGACATTCAACAATTAAGATCAGAAGTGATTAATGCGTTTAAACTTGGTGGTTTCACGTTGAGACGTGAAGC
CAGTACCTTTCTAGTAGAACATGTACAAGAATTAACATACCAGGAGAGAAAGAAACTTATAGATAAGTTGATAACCC
ATATTTTACACCAGTGTTTATCCCAACCAGTACTAGAAAAGTCTCAACTTGAACTTGCAATTAAAGAGTGTTCTTCT

TTAGGATTAGAAGAATCAGAATCTATTTTAAATGTTATTGATGCATTTAGTGTACCTAAGTTTTGTTATGATAATGA
AAGGAAGAAATTTGTAAAAAATATTTACGACTCAAAAAGGCTTTATCCTGAACCAAAATGGAAATCACATATGTTAA
TAGACAGGTACACAATTATTTGGCAAAGAACTGCTAGAAATAAATTATTTGCGGCCAACACATTACCATCAATGGAG
AATGAGAATCAGTTTCAGCTGCGGAAAATTGAGGTTCTTTTGAGCAGCTCAAGTAAAGTCAATGATGTGATAGTATT
GGGATTGTTAACACAACTAACTGAAGGTAAATTTTATTTAGAAGACCCAACAGGTAGTGTGCAATTAGACATAAGTG
AGACAAGATACCATTCTGGGCTATTTACTGAAAGTAGTTTTGTATTAGCTGAAGGCTATTACGAAGATAAAGTCTTA
CATGTGATGGGCTTGGTTCTGCCTCCCACAGAAAGCAGAGCCATGTCTATGCCATATTTTGGCAATCTAAATACTTT
TGGTGGACCATCAAAGACTTTGTTGAAAAATTCTCAAAATTTACTCAAATTAGAACAAGAAAATGAAGATGGTATTA
TTGTTTTTCTGTCTGACGTTTGGTTTGACAATTTAAAAGTTGTGTCCAAGTTAAAAACCTTATTCTCCGGGTTTAAT
GATTATCCGCCAATTGCCATAGTATTTTTGGGTGAATTCCTCTCATGTCCATATGGGTATGAACATAGCACACAATT
GAAGGCAGCTTTAACAAACTTTAGTGACATTATTTTGCCATTCACAAAACTACGTGAAAGCTGCAAGTTTATATTTG
TTCCTGGGAGTGGAGATCCAGCTGCTGCTAACATTTTACCTAGACCAGCATTACCAAACTTTGTTACTGCAAATGTA
AAGGACAAGTTAGGTGATAGTGTTGTTTTTACAACAAATCCATGTCGTATTCAATATTGTGCACAAGAAATTGTTGT
TATCAGACAGGATTTAGTGACAAAAATGTGCAGAAACTCAATACATTTCCCTGAAACTGGAGATATACCAGATCACT
TAACAAAGACTCTTTTAAGCCAATGTACATTATCTCCATTGTCTATAGGTGTACAACCAGTATATTGGAAATACTCA
GATGCATTAAACTTATATCCTATTCCTGATTTGGTGGTAGTTGGTGATCATTTCCAGCCATATACTAGGTCATATCA
GAACTGCCAAGTTACTAATCCTGGGTCATTCCCTCGAACTGAATTCTCATTTAAAGTCTACATACCAGCCTCTAGAA
CAGTGGAAGACTCTCAAATACCCAATGATGATACT

Befrostia:
>cne2576.1.37:A2012G | Twitchin
ATTGAAGGTGATGTTACAAGTGGAAAAGTACCAGATCTTATTGAAGGCAACACGTATGAGTTCCGCGTGCGTGCTGT
TAATAAGGCAGGAAAAGGCATTCCAAGTGACGGAACTGGACCTCATCTTGCAAGACCCAAAAACTTACCACCTAAAA
TTGACAGAAACTTCATGTTTGATATCAAAATCAAAGTCGGCCAAAACTTTGAACTGGATGTTCCAGTTGCTGGAGAA
CCAACGCCTTCAAAGGAATGGCTTCATGGTGACAATATGGTTCTAAACACAGACAGAATTAAAGTAATTAATGATGC
TCACTCAACCAAAATCAGAGTTATTGATGCCAAACGATCTGATACCGGCACGTACACACTCAAAGCGAAGAATATTA
ACGGAACTGACTCAGCAACTGTTAAAATTGTTGTCATGGATGTGCCGCTACCACCAGAAGGTCCACTGCGTGCGGAT
GATATTACAAAATCTGGCTGTACTCTAAGATGGAGGCCTCCTAAAGATGATGGTGGCTGTGAGATCAGTCACTACGT
TGTTGAGAAAATGGATCAAGACAATCTTCGCTGGGTGCCAGCTGGAGAAGTGCAAGGTTGTCATATCCGCGTTGACC
ATTTGATTGAAGGTCACGATTACAACTTCCGTGTCCGTGCTGTTAACAAACAAGGTGAATCGCAACCACTTGTTGGC
CAAGAACCAATTACCGCCAAGGATCCTTATGGAACACCAGACAAACCTGGAACACCTGTTGTCAAAGACTGGGATAA
AGATCACATGGATCTGGAATGGGTACCACCAAAGAAAGACGGTGGCTCACCAATCACAGGATATGTCATCGAGGCAA
AGAAGAGGTACGGACCTTGGGAAGTTGCTGCAACTGTTACTGGTAATAAAACAACTGCAACAGTACCAGGGTTGCAA
GAAGGTGAAGAGTATGAATTTAGAGTCATTGCTGTTAATAAGGCTGGTAATGGCGAGCCTAGTGATGCTTCTACGCC
ACAAGTGGCTAAAGCGAGATTCTGTGTTCCAACCTTTGATAAAACGCTTCTCGCTGATAAGACTGTTAAAGCGGGTC
AAAGAATCCAATATGATATCCCGATTGAAGCATCTCCAAAACCTACAGTAGAATGGCAAATTAACGGTAAACTTGTT
CATCCGTCTGAAAGGATCGACGTTCAAGTACTGCACAATAGGGTCATTTTAGACATTCCATTCTCTGTTCGATCTGA
TGGTGGAGTATATAAACTCACCCTGAAGAATGATTTGGGAATATGTTCAGCATCTGCTCAAGTTACCGTACTAGACA
AACCATCAAGGCCCGAAAAGCCTTTGGTAGTATCTGATATTACCAAAGATTCTTGCTCATTATCATGGAAGGTACCT
TTGGACGATGGTGGATCGCCTATTTTGCACTACGTTATTGAAAAGATGGATCTATCTCGAGGTACGTGGTCAGATGC
TGGTATGAGCACCTTCCTTTCGCATCAAGTGACAAGACTAATTCACATGAAGGAATACTTATTCAGAGTAAGTGCTG
TTAATGCTATTGGTCAGTCTGAGCCGCTAGAATTAGAGCGTGCTATAGTAGCTAAGAATGAATTTGATGAACCATCA
GCTCCAGGTAAACCAGAGGCTACGGACTGGAGTAAGAATCATGTCGACTTACAATGGGCTGCACCCAAATCTGACGG
TGGTGCACCAATCACAGGATACATTGTCCAGAAAAAAGAAAAGGGCAGTCCATATTGGGTCAATGCAGCGCATGTAC
CTTCTGGTAAGACGAAAGCTACCATTCCTGATTTAACAGAAGGTCAAGACTACGAGTTCCGTGTTATTGCTGTGAAC
CAAGCCGGACAATCTGAGCCTAGTGAGCCTTCAGATATCATTACAGCCAAAGACAGGTTCGTGGCACCAAAGATTCT
TACACCACTTACAGAAATTCGTATTAAGGCTGGTCTTATCTTCCATTGTGATGTTAACTTTATTGGTGAACCGATTC
CGGAAGTTAAATGGACTTGTGATGGCAAAGAAGTTCTTACTAATGCTAGAACCACTGTCACATCTGTTGGACATCAT
ACAATTGTTCATACTGTTAATTGTAAGAGATCAGACGCAGGTAAATACCATCTATCACTTAAAAACGATTCTGGTTC
TGATGAAGGTAGCTTTGAACTGATTGTATTGGATGTTCCTGGACCACCACAGGCTCCATTTGAATATGAGGAAATCA
CTGCTCAATCTGTTACACTATCATGGAAACCACCTAAAGATAATGGTGGTAGTGAACTCACAGCTTACGTCATCGAG
AAACGCGATTTGACTCATGGCGGTGGCTGGGTACCCGCAGTGACATACATCAATCCAAAATATAATCACGCAACTGT
TCCTAGATTAACTGAAGGCACAAAGTACGAATTTAGAGTTTTTGCTGAGAATTTACAAGGACGCTCTGAACCATTGG
TTACTGATAAACCAATTATTGCAAAGAATCAGTACACAGTGCCTGGAAAGCCTGGCAAACCAGAGCTTGTCTCTGCC
GATAAAGATCACATTAAGATTAGGTGGTCATCACCTATTTCAAACGGTGGATCTAATATTATTGGCTACGATATAGA

AAGAAGAGACGTAGCTACAGGTCGCTGGATTAAGTTGAACAGAGATCCAGTGAGATTCACTGAATATGATGACGATA
GAGTACAAGAAGGTCATCAATATGAATACAGGGTATCCGCTGTTAATGCTGCGGGTGCAGGTCAAGCTTCAGATTCG
TCTAACGTGTTCATTGCCAAACCAATGAAGGAGAAGCCGAAATTACATTTGGACCACCTTATTGGACGTAAGATCAA
GGTCAGGGCGGGTGAACCAATAAACATTAACATTCCAATT

>cne792.13.4:A310G | Nucleolar protein 11
AGTGATGCAAAAATGACTATGTTTGACCTTTTAAAGAAGACTTGGAAGACAATTGGTACTGTACCTTGGATTTCAAC
TACCAACAGTGTGTCAATTGCTTGGATGGGTAAAAACCATCTAATATTGTTTGGCAGCAACTGTGATCAAGATGGAG
CTATAATTGTAGCCTATAATGTCATCTTAGGAGTTGGGTCTTGCAGATATCCTATGAAATTGTACTCTGATAATGCT
CAGTTATGTTGCTTTAAAAATCATATAATTTTGGAAGCTTCTAACCATATTGGAATGCTACCATTTGTACTAGAAAG
TAACAGAAACTTGTCAAGCCTTTTAGGGTCTCATGATATAGTGGAAGATAACAGTACAGAGATAACTGATTGGGATA
CACCAGTTGAACCTAAATTTCAGGTTAGTAATGAACTGAAAGATCTTTTAAAATTAGGCCTGTCGGAGAGGAATATG
TGTGCTCAATTAATTTTGCCATTGCTGGAAAAAGGTGATATTTGTCGAATATTCAAAGCTATTAAAGAATTTAATGA
TGTACCAGAGCTAGTTTTAGTGGCTTTGCTGCATTACACTTTAAAATCGTTACCTTTAAAAAATGTTGATTTTACTA
ATGATGATGAATTTTTACAGTTCTGTAAAAGGGCTGATATATCTAAGAAAGAATCAGCTGTACTAAAAACTAACTTC
GAGTTATTAAATTATATATTTCAGTTAGACTTTAGTGATGCTGTTATGATACCACACTTAAGAACAACATTACATTT
GGATGATGCATTGTTTTTGTTACGATATATTTCATATTTACTTTGTGAATCTGATGAAATAATTGAAAATATTTATG
AAAGTAAATTATTTGATTGGTGCACTTTGCTGATGGATGCATTTTATCAGCAATATTTATTGACCAAGGACAAAAAA
GTTGGTAATGTTTTAGATCATATGCAAAAAGTTGTTGTAAACCTAGTTGATCAATTATTGGTTGTGAATGATATTTT
ACCAACATTGCAAAAGACAATTTCAGGAGATAAACCTAACGATAGTGAAGAAACCTTGTCTTATGGTATTGAATTAA
TTGAAATA

>cne4106.6.1:G277C | Protein unc-45 homolog B
ATGGGTCACGCTGAAGAAGCTGAAAACTTTAAAAACAAGGGAAATGAAGCTTTTAAATCTGATAAATATGAAAAAGC
CATATCTTTGTACAGTGAAGCTATAAAATTGGCCGAAAAAGGAACACGTGATATGGCCACTTACCTGAAAAATCGTG
CAGCTGCTTATCTCAAAGTAAGAAACTACGAGAATGTTATCAAAGATTGTAATGAAGCTCTGGAGGTTGTCCCCGAG
GATCCAAAAGCGCTCTTTCGTCGTGCTCAAGCTTTCGAGAATCTCGAAAAATTCGAAGAAGCGTATCGTGATGCTAA
AACGATCTTCACAGTAGATCCGACCAACAAAGCTGTGCAACCAATCCTGGCACGACTCTATACAATAGTACAGGAAA
GGGCTAAGAAAAAACTCACAGACAACTAGTAAAGTTGAACAGATGTTCAAAGTGGCATTTGATTTATCTGAAGATACA
GAGAAACGGTCTAAAGCAGTGTCAAATTTATTAGTTTTAGCTAAAGAAAATACTGGAGCTAATATTATGATTCAAAA
TGGTGTTATTGTCAAAATACAACAGCTTTTAAAAGTTGAGAAAAATGTAGATATTTATATTAATGCAATAAGAGTAG
TTGGTCAACTTTGTAAACAAGATATAGAAAGAACAAAAAATGTCATAAAAGTAGTTGGTATTCCTTGGTTTTTAGAC
ATAATTGACAGTGATAATGAAGAAAGAGTTAACGCTGCACAATATTGCATTCAGATTATTTAAATACATTTTCAGG
AATGGAAAATGGTAAGGAAAGTAAACCAGATGCAGCAATGTGTGAGCAACATAAAGGAGAAATTGATACTCTACTCA
CATGTTTAACATATTCTATCACAAACCGTGTCATCACTGGTCTTGCTAGAGATGCAATAATGGAATTGATTATGAGA
AACTGTCATTATAGTACTTTGAATTGGGCAGAGAGGTTTATTGAGATCAGAGGCTTATTAAGATTACTGGAGGTCTG
CAGTGAATTAGAAGAATACAAATATGAATCAGCTATGTCTATAACTCCATCATCAAGAACTATTGCTGCTGCATGCT
TGGCTAGGATTTATGAAAATATGTATTATGATCAAGCAAGAGAGAAATTTAATAGTCAGGTTGATGAATATGTCAAA
GATAAATTATTGACTCCAGATTTAGAATCTAAAGTTCGAGTAACTGTTACTATAACTTCTTTGTTACGAGGGCCCCT
TGATGTAGGTAATTATGTTATTTCAAAAGAAGGTATTATGGAAATGATTTTAGTTATGGCTCAGACGGAAGATCCTT
TGCAGCAAAAAGTTGCTTGTGAATGTTTAATTGCAGCTGCGTCCAAAAAAGATAAAGCTAGAGCTATTATCAATAAA
GGCGTTGATATATTAAAAAAATTATACGCTTCTAAAAATGATGCAGTAAGAGTTAGAGCTCTAGTTGGATTGTGTAA
AATTGGTAGCTTTGGTGGGGATGATGCTTCAATTCGCCCCTTTGCTGATGGTTCTACAACAAAACTAGCAGAAGCTT
GTCGGAAGTTTTTAGTCAATCCAGCTAAAGACAAAGATATGAGGAAATGGGCAGCTGAAGGTCTTTCGTATCTTACT
TTAGATGCAGATGTTAAAGAAAAGTTAGTAGATGATAAACCAGCTATTCAATCACTCATTGAGTTGGCTAAAACTGG
TGATCAATCATGCCTTTACGGTGTTGTTACAACACTGGTGAATCTTTGCAATGCTTATGAGAAACAGGAAGTTATGC
CTGAAATGTTAGAATTAGCAAAGTTTGCTAAGCATCATATACCTGAGCAACATGAGTTGGATGATCCTGATTTTGTG
AGCAAAAGGTTGACTATTCTTTGTAAAGCTGGGGTAACTACAGGTTTAGTTGCATTGTCAAAAACAGAGAGCCATAA
TTCAAGAGAACTGATTGCAAGGGTGTTTAATGCTTTATGTAGTCTTCAAGAACTTAGAGGTATAGTAGTTCAACAAG
GTGGTGCTAAAGTTTTAATACCAATGGCACTTGAAGGGACTAACAATGGGAAAAAGCAAGCAGCCCAAGCATTGGCT
AGGATTGGAATTACCATCAACCCTGAAGTAGCTTTCCCTGGTCAAAGGAATTTAGAAGTGGTTAGGCCTTTGGTTGC
TCTCCTTCATCCTGATTGTACAGCATTGGAGAATTTCGAAGCTTTGATGGCCCTATGTAACTTAGCTGGTATGAATG
AAACTACAAGAAATAGAATCCTGAAAGAAGGTGGATTAGCTAAGATTGAACATTATTTGTATGAAGATCACTTAATG
CTTGTGAGGGCCGCTACTCAGTGCATTTGTAATTTAGTTCAATCTGAAGAAGTTATAAAAATTTATGAAGGAAATAA
TGATAGGACAAAATATTTATATGTGCTTTGTCAAGAAGAGGATACTGATACTGTCATGGCTGCAGTAGGGGCATTAT
GCACTTTGACTTCTGGAAGTAAAATTTGCTGCAGGAAGATGATGGATGTCCAATCATGGCCTGAAACACTTAGGTGT
CTACTTGCTAATCCTAATTCCGAAATTCAATACCGTGGAACATACATGTTGTATAATATTATCAATGAAGACATGGA

TGCAGCTACAAAAATTTTTGAAACAGATGTGATGGAAATATTAATGGCCTTGACAAAATTAAGTGACCCACAGCAAC
AAAAAATTGTAGATTATTCCCAAAAATGCTTAGATGCTGCTGAAAAAATGGGAGTAATTCGAAAGCCAAATGAG

>cne3195.1.6:T355A | Receptor-type tyrosine-protein phosphatase N2
CCGTTGACTAAGGAAGAAGAAGTATTAGTAGCCGACGAAGAGACGCCTAGTGACAAATTACGAGATTTGTTAATGTT
GAGTTCAGTTGAAGATTCTCCCGTCATACCACAGTTTGAAGGATTCAGAGAACGCCTACAAGAAGAACAAAAAGCTA
GAATTCATAATATTAAGGATGTAATTCCTGATGAGTTTGCTGACAAAGAAAAGAAGTCATTAGCACAAACAAAACTA
GAACAAAATCCTAGAGAGGATAATGCTAAATACACTGATGAAGAGCGTTTAGGAGCTCATTATAGAAAATACAAAAT
AAAACCTCCACCGTTTACTGCTGAGTATTTAACAGCTAATAGATTTTCACCTCTAGATGCTGAAGTTCGTTCAAATG
CACTAGAAAAATACAAACTAAGTTTTGCAGAGAAAAATTTTCCATTTGAGTATGAAAATCCTGAAGATTTTCAAGAG
CGCAGAATTTACTTTAATGACGATGGTACTGAAGAACTAGCTTCTGACGCTGGCAGCGGTGAGTTGGATCCATATCA
TTTA

>cne2174.2.11:A93G | Transcriptional activator cubitus interruptus
TCTGAAATGGTCTCAAGCACAACTCATCCTCTGCAAGGAGGAGATAACATGGTTGTAAATGATCTGCAAACTTCTCT
ATCTTCATTTTACGAAGAAAATCAATATTTGCAAATGATACAA

>cne306.12.2:G89T | Peroxisome biogenesis factor 1
CCAGAATCCATACAAATATGTTATAAAGACAAACAGATACTGGCTTGGGCTATGTTCAATGCTGCAATACCAGATGG
TGAGATATCGTTCAATCCATTATTCTGCAAGCATGTTGGATTAGAAGAGGGTGCTGAAGTGTTTGTGTCTCCATATC
CTGATGTAAAAATTTTAAAAGAGATCTACATTGATGCAGACAGTGCCGATGATCAAGAAATATTA

>cne3772.18.1:C271A | Peroxisomal membrane protein PEX16
ATGTGGTTAACAATCATCCACTATTGTGAAGTATTTGTTGAATTAGCTGTAAAGAACAAATGGGGTGTGCAAGGAAA
ATGGACAGCTGCAGGATTAATACAGCTATTTAAATGTTCCTCCGCTTTCATTCTTTTATACCGATTCAAAGAATTGC
CCATTCAACATCCACCTATACCAGCTCTTCAAAGAAAGAAAATTATTGAAGGTAGAGATATAGATGAAAACTCTAAT
GCCTTTTTCACATTACGACGATCTGGTCGCATTATAAGGAGAGTAGATGGTGCTCCCCCTGTGGGTCTGCGAGATTG
GCAACCTATAAAAATTCAAGATGAAAAATCTGGATATGGTATTGAAGTTAAAGAATTATTGAGGGCTGAATTGTTGC
ATATTTTTAAGCCATTGATACACTTGGCTGCCATGAGAGTGTTCGGAACTAAAACCTGGAAACAATGGCTAGTTGCC
TTGGGTATTGATATAGCTAGCTTTAGACTTTATAATCGTCATATGAAGACATTGTCGTACGAACAAAGATTGGAAAT
AAGTCGTAGAAAACTAAGTTTAGTCCTATACTTGTTGAGAAGTCCAATGTACAATGGGTATTCTAAAAATGTTATAG
AGAACACTCTATTGACACTTTCAAAGAAAATTCCTTTGATGTCATTTATATGTGGCCCAATAATTCAGTACCTAAGT
CATTGGCAAGACATTTACTTTTACATGTGGACTTCA

>cne26635.1.11:A509C | TBC1 domain family member 1
TCGGCAATGGAAGCAATATCTTCATATCAAAAAACTCAAACAAAATTAGAAACTCAGAACAAAATCCTAGAACACCA
CGTGACTTTACTAAGTCGATACATATTAGCCAATCACAAGCAAGAGATACAACCTGAAATCAAAAAAATAGTACAAA
ATTATGGCAAAAAACTAACATTCAGTTCTAAAATATCATCTTTCACAAAATTCGCATCTAATGAAGAAGAGGAAGAT
AAAAAGATATTTAAAACAGGTGTCAGTACTCCAAATCTATTTGCTAAAACTACGAGAGAAGAAGTTTTAAATGCAGC
AAATAAAAATGAAGACAGAAGACAGTTTATGATGAAAAAATCTCAATCAATACATTCTGGATTAATAGCTAAAATTC
CTAAAGCACAAGATGAACACAGACCATCACCAAGAAAGCATAGTCTAAGAATCGAAGATTTGGATTCCTCAAATTTA
AAAAGCAAAAGCTATTCATCCCAAACTCAAGAACAAATTCGTCAAGCAAGACTATTCGAACAACAACTCAAACACGA
TTTTGATGAGAATCTCAAAAAACTTGACGAAAAACTGAACAAATCTTATTTAGATGATTTAACAATATTCCAGGGGA
AAAAAAGCATGTCGTTGAATTTCAAAGCCGAAAAAGAAACGAAATCAATAGACAGTGGTTTTGTAACACCGGCAAGT
CCTAGAGAAGATAAAAAAGAAGATGGGTCAGTGATTTCGCATCCTCTAAGTGATTGTGATGTTGATATCAAGTTTGA
TGGACAATTAACTAAGTTAAAACAAATAAGGCCATTAAAAATAAATAATATGAAT

>cne1086.2.12:G82G (not A) | Tetratricopeptide repeat protein 21B
ATATACGAGAAGGAACAAAATTACAAACTTGCTGCACATAATTTTGACAATGCTTGGGTGTACTCGTGTAAGAATGA
TTTGAGCGTCGGATACAAACTCGCCCAATCCTATCTAAAAATCAAGAAATATCCCGAGTGTATAGTCGTCTGCCGGC
ACATACTCAAAGTACACCCCGACTACCCGAAGCTGAAGAAAGAGATCTTTGAAAAGGCTAAGACAAATTTAAGGACT

>cne23980.1.3:G59G (not A) | Zinc finger protein 177
GCCCATGTAGCAAACATGCACAAACCGAGCACTCCTGTAGTATGCAGTCATTGTAAAAAAACCTTTAAGAACCAAGA
GAAACTAGATATTCACTGGAGAAAATTGAAACAAAATAAACCCTTTGAATGCAGTATATGTTTGAAGAAATACGCCC
TTGAATGTGAGTTGAAGAGCCATTTGTTTTGGCATACAGATGAAAGACCACATGTATGTCAGCTCTGTGGGTCGAAG
TACAAAGCTAAGGGGCAGTTAAAGGTTCATATGCAAAGTCATACAGGGGAAAAACCGTACAAATGTGACTTGTGCGA

TAAGAGTTTTTACACTGGGACGCAGGTTAGGAGGCACAGCAGTGTCCACACTGGAGTACGAGCCCATAAGTGCTTGT
ATTGTCATAGGTCTTTTCATAATCGGAAAGTGATGTTAGCTCATTCTCTACACCACAAAAGTGAACCGAAGTCTAAA
GCAATA

>cne4786.7.1:C158C (not A)
ATGCGTAAAATAATAGTATTTGCATATTTTGTTTACTACGTCCATTGTGCCAGCTATCAAAGTTATGAAAGCACACG
ATTTCCGGCATATTCAACGATTTCAAATTTATACAACCTAAACCAATTTCCATACAAAACCAACTATGGAAGCGATA
TCGAAACTTTGCCAGAAGCTACACGATATCCCAATTATAAACCTGGTTATTCCACTCTAAGCCCAGGTATCTATAGC
TCCAGTGATGCAGCAGGAGGTTCTGGTATTTATAGTTATACGACTCCGAGATATGGCCAAACCTATGGCACAGGACA
AAAATACAAAGGAGGATACAGTGGACAAACTTATGGTCAAGGATATAGTGGATCAAGTTATGGCCAGGGATTAGGGC
AAAGTTATGGCCAAGGCTACGGTGGACAAACTTATGGGCAAGGTTATGGCGGTCAGACATACGGTACAGGATATAGC
TCATATGAATTGCCGTTTATGAAGTACAACAAGGATTATTGTGTCAATAGGCTACCTCAGAAGGAGATATTTATAAG
TAGTCTGACTGGTATGTGGTATGGCGTGGAGTTTATCCAACATTTGGCTGGAGATTCAAGGGTAGATTATGCGAGAA
CATGCATCGTGATACACATTTCGGAGCCTTTTGATAGG

>COI barcode reference sequence, many positions are used as characters
AACTTTATATTTTATTTTTGGAATTTGAGCAGGATTAATTGGAACTTCATTAAGTTTACTTATTCGAACTGAATTAG
GAACTCCAGGATCTTTAATTGGAGATGATCAAATTTATAATACTATTGTTACAGCTCATGCTTTTATTATAATTTTT
TTTATAGTTATACCTATTATAATTGGGGGATTTGGAAATTGACTAGTACCCCTTATATTAGGAGCCCCAGATATAGC
TTTTCCTCGTATAAATAATATAAGATTTTGATTATTACCCCCATCTCTAACTCTTTTAATTTCAAGAAGTATTGTTG
AAAATGGAGCAGGTACTGGATGAACTGTTTATCCACCTTTATCTTCTAATATTGCCCATCAAGGAGCTTCAGTAGAC
TTAGCAATTTTTTCATTACATCTTGCAGGAATTTCATCTATTTTAGGAGCTATTAATTTTATTACAACTATTATTAA
TATACGAATTAATAATTTATCTTTTGATCAAATACCATTATTTATTTGAGCCGTTGGAATTACAGCTTTATTATTAT
TACTTTCATTACCTGTTTTAGCTGGAGCTATTACTATATTATTAACTGATCGAAATTTAAATACTTCATTTTTTGAT
CCTGCAGGTGGAGGAGATCCTATCTTATATCAACATTTATTT
